# Rational Design and Synthesis of D‐galactosyl Lysophospholipids as Selective Substrates and non‐ATP‐competitive Inhibitors of Phosphatidylinositol Phosphate Kinases

**DOI:** 10.1002/chem.202202083

**Published:** 2022-11-24

**Authors:** Mengxia Sun, Chi Zhang, Dexin Sui, Canchai Yang, Dohun Pyeon, Xuefei Huang, Jian Hu

**Affiliations:** ^1^ Department of Chemistry Michigan State University Michigan MI 48824 USA; ^2^ Department of Biochemistry and Molecular Biology Michigan State University Michigan MI 48824 USA; ^3^ Department of Biomedical Engineering Michigan State University Michigan MI 48824 USA; ^4^ Institute for Quantitative Health Science and Engineering Michigan State University Michigan MI 48824 USA; ^5^ Department of Microbiology & Molecular Genetics Michigan State University Michigan MI 48824 USA

**Keywords:** drug design, inhibitors, non-ATP-competitive inhibition, phosphatidylinositol phosphate kinase

## Abstract

Phosphatidylinositol phosphate kinases (PIPKs) produce lipid signaling molecules and have been attracting increasing attention as drug targets for cancer, neurodegenerative diseases, and viral infection. Given the potential cross‐inhibition of kinases and other ATP‐utilizing enzymes by ATP‐competitive inhibitors, targeting the unique lipid substrate binding site represents a superior strategy for PIPK inhibition. Here, by taking advantage of the nearly identical stereochemistry between *myo*‐inositol and D‐galactose, we designed and synthesized a panel of D‐galactosyl lysophospholipids, one of which was found to be a selective substrate of phosphatidylinositol 4‐phosphate 5‐kinase. Derivatization of this compound led to the discovery of a human PIKfyve inhibitor with an apparent IC_50_ of 6.2 μM, which significantly potentiated the inhibitory effect of Apilimod, an ATP‐competitive PIKfyve inhibitor under clinical trials against SARS‐CoV‐2 infection and amyotrophic lateral sclerosis. Our results provide the proof of concept that D‐galactose‐based phosphoinositide mimetics can be developed into artificial substrates and new inhibitors of PIPKs.

## Graphical abstract

**New‐to‐Nature D‐galactosyl** lysophospholipids were rationally designed and synthesized to act as artificial substrates and non‐ATP‐competitive inhibitors of phosphatidylinositol phosphate kinases, which are validated drug targets for deadly human diseases including cancers, amyotrophic lateral sclerosis, and SARS‐COVID‐2 infection. Rational Design and Synthesis of D‐galactosyl Lysophospholipids as Selective Substrates and non‐ATP‐competitive Inhibitors of Phosphatidylinositol Phosphate Kinases (X. Huang, J. Hu et al.)

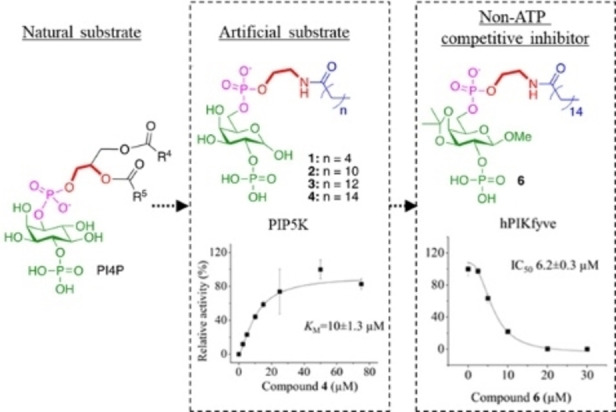

## Introduction

**Scheme 1:**
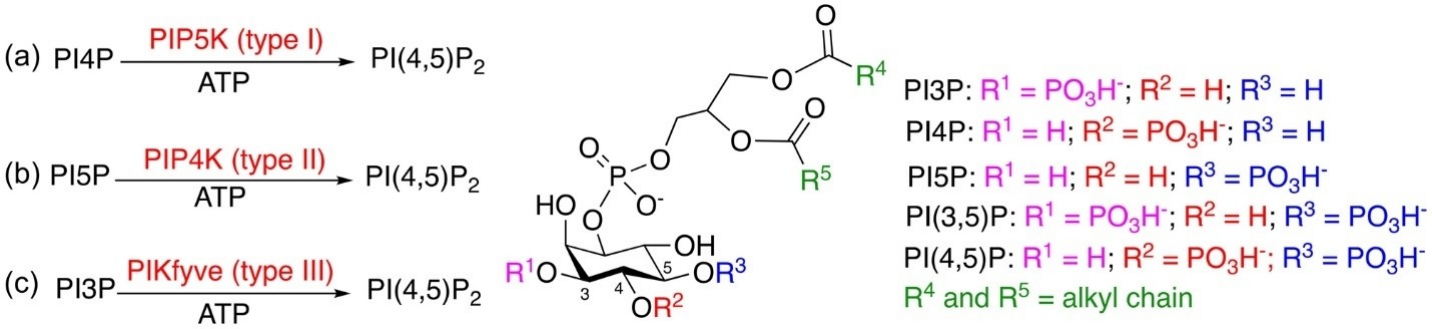
Schematic representation of PIP (PI3P, PI4P and PI5P) phosphorylation by PIPKs. (a) Phosphorylation of PI4P by PIP5K (type I); (b) Phosphorylation of PI5P by PIP4K (type II); and (c) Phosphorylation of PI3P by PIKfyve (type III).

Phosphoinositides are phosphorylated derivatives of phosphatidylinositol, constituting a group of minority phospholipids in the cell membranes. A total of seven naturally occurring phosphoinositides play crucial roles in cell signaling and participate in numerous physiological and pathological processes.^[1]^ Three lipid kinase families, including phosphoinositide 3‐kinase (PI3K), phosphatidylinositol 4‐kinase, and phosphatidylinositol phosphate kinase (PIPK), are responsible for biosynthesis of the phosphoinositides.^[1b]^ The PIPK family members synthesize all the three types of phosphatidylinositol bisphosphates (Scheme 1).^[2]^ Phosphatidylinositol 4‐phosphate 5‐kinase (PIP5K, type I) produces the majority of PI(4,5)P_2_ using phosphatidylinositol 4‐phosphate (PI4P) as the substrate^[3]^ (Scheme 1a); phosphatidylinositol 5‐phosphate 4‐kinase (PIP4K, type II) generates PI(4,5)P_2_ using phosphatidylinositol 5‐phosphate (PI5P) as the substrate^[4]^ (Scheme 1b), possibly contributing to vesicular production of PI(4,5)P_2_;^[5]^ the third family member FYVE finger‐containing phosphoinositide kinase (PIKfyve, type III) phosphorylates PI3P to produce PI(3,5)P_2_ (Scheme 1c), a phosphoinositide with very low abundance but critically involved in endosomal trafficking.^[6]^ Accumulated evidences have shown that PIPKs are potential drug targets for cancers,^[7]^ amyotrophic lateral sclerosis (ALS),^[8]^ chronic pain,^[9]^ and viral infectious diseases,^[10]^ including the SARS‐CoV‐2 infection.[^10b^, ^11^] As such, it is urgent to develop selective PIPK inhibitors for clinical applications and also as research tools to study these functionally important kinases.

A common strategy of developing kinase inhibitor is to target the ATP binding site, which is a deep cleft sandwiched by the *N*‐terminal and the *C*‐terminal lobes conserved in all eukaryotic kinases. Inhibitors targeting the ATP binding site have been reported for different PIPK family members.[^7c^, ^9^, ^12^] However, the fact that more than 500 human kinases share a similar ATP binding pocket imposes a challenge to develop highly selective ATP‐competitive inhibitors. Other ATP‐utilizing enzymes may also be affected by kinase inhibitors.^[13]^ As the intracellular ATP concentration is in millimolar range^[14]^ and the *K*_M_ values for ATP of a large majority of kinases fall within the range of low to medium micromolar,^[15]^ an effective ATP‐competitive inhibitor must have a high affinity with a dissociation constant of low nanomolar or better to effectively compete with intracellular ATP. To circumvent the potential pitfalls associated with ATP competition, an alternative strategy is to target the substrate binding site, which can be unique in geometric and electrostatic properties among the kinases as they phosphorylate distinct substrates. Targeting the substrate binding site has been demonstrated to be an effective approach to develop kinase inhibitors with high selectivity.^[16]^ In addition, additive or synergistic effects among inhibitors with distinct working mechanisms would lead to better therapeutic effectiveness and lower adverse effects when they are applied together.

**Scheme 2:**
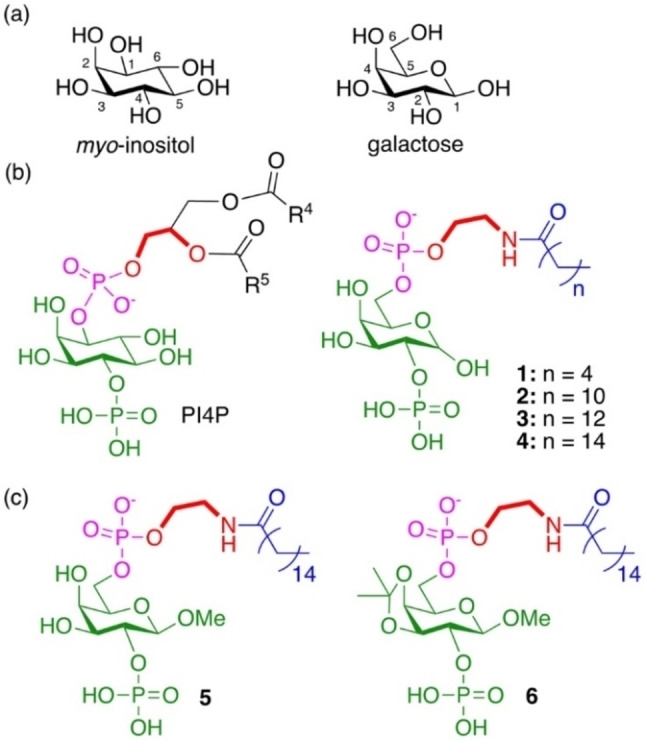
PI4P mimetics synthesized in this work. (a) Comparison of stereochemistry between *myo*‐inositol and D‐galactose. (b) Comparison of PI4P with designed 2‐phosphate D‐galactosyl lysophospholipids (**1**–**4**). (c) Derivatives of **4** (i.e., compounds **5** and **6**) designed as potential lipid substrate competitive inhibitors. The equivalent components are shown in the same color and the ethanolamine linker is bolded.

To date, there have been no prior studies aimed at the substrate binding sites of lipid kinases for inhibitor development. In this work, we designed and synthesized novel lipid substrate mimetics to target PIPKs’ substrate binding sites by taking advantage of the nearly identical stereochemistry between *myo*‐inositol and β‐D‐galactose (Scheme 2a). Specifically, we produced a panel of D‐galactosyl lysophospholipids **1**–**6** (Schemes 2b,c) to mimic PI4P, the natural substrate of PIP5K, as a new strategy to develop PIPK inhibitors. Our data showed that (1) one of the PI4P mimetics was efficiently phosphorylated by PIP5Ks in the same manner as PI4P, but it could not be phosphorylated by PIP4Ks or PIKfyve, indicative of an artificial substrate with a higher selectivity than PI4P; and (2) derivatization of the PI4P mimetic led to the discovery of a non‐ATP‐competitive pan‐PIPK inhibitor with a preference towards PIKfyve, which additively reduced the activity of PIKfyve with an ATP‐competitive inhibitor, Apilimod. These findings open the door toward developing D‐galactose‐based phosphoinositide mimetics as selective inhibitors of PIPKs and other phosphoinositide‐processing enzymes, many of which have been validated as drug targets.^[17]^

## Results

### Design and synthesis of D‐galactosyl lysophospholipids 1–4

**Scheme 3:**
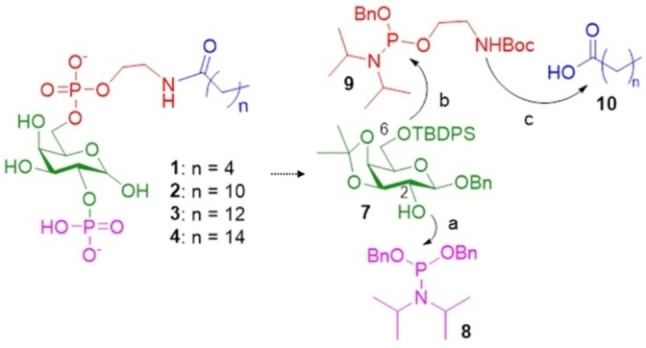
Retrosynthetic design of D‐galactosyl lysophospholipids **1**–**4**.

Our first designs were a series of the PI4P mimetics **1**–**4** (Scheme 2b) with varied length of the alkyl chain. To enhance the overall synthetic efficiency, we envisioned that the 2‐*O* and 6‐*O* positions of galactoside **7** can be functionalized sequentially with phosphoramidites **8** and **9** first (Scheme 3). Phosphoramidite **9** bears a t‐butoxycarbonyl (BOC) protected ethanolamine linker. Upon formation of the phosphate esters, the Boc group could be removed to generate the free amine, which could be amidated to divergently produce PI4P mimetics **1**–**4** with varying chain length.

**Scheme 4:**
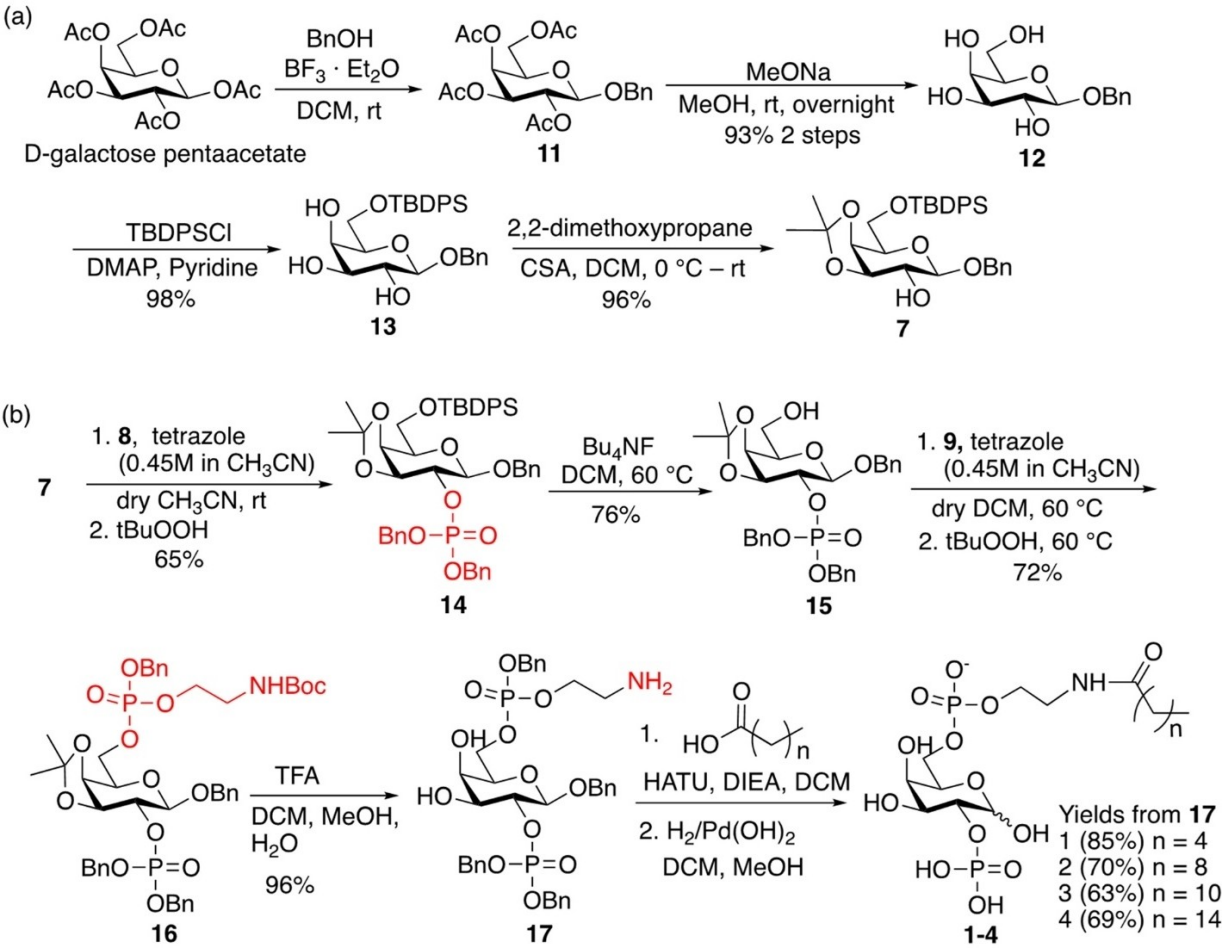
(a) Synthesis of the D‐galactoside building block **7**; (b) Synthesis of the D‐galactosyl lysophospholipids **1**–**4**.

To synthesize the galactoside building block **7**, we started with the commercially available D‐galactose pentaacetate (Scheme 4a). Glycosylation of D‐galactose pentaacetate with benzyl alcohol promoted by boron trifluoride‐diethyl ether complex (BF_3_ ⋅ OEt_2_) provided the corresponding β‐benzyl glycoside **11** (^3^J_H1‐H2_=7.9 Hz).^[18]^ The reaction should be quenched in 4 h upon complete consumption of the starting material to obtain the β‐glycoside as the major product, as prolonged treatment led to the formation of the corresponding α‐glycoside as the major product presumably due to *in situ* anomerization of **11**. Global deacetylation of **11** under the Zemplén condition followed by selective protection of the primary OH with *tert*‐butyldiphenylsilyl moiety (TBDPS), and protection of 3,4‐diols with 2,2‐dimethoxypropane gave the desired galactoside building block **7**.

The phosphorylation of 2‐OH of galactoside **7** was performed *via* a one‐pot protocol, using dibenzyl phosphoramidite **8** and *t*BuO_2_H as the phosphorylating and oxidizing reagents (Scheme 4b), respectively, to furnish the mono phosphorylated compound **14**. After removal of the 6‐*O*‐TBDPS from **14**, the second phosphate group was installed with the freshly made phosphoramidite **9** (Scheme S1), followed by *in situ* oxidation to afford the diphosphorylated compound **16**. The BOC and isopropylidene groups of **16** were then deprotected by treatment with trifluoroacetic acid (TFA), and the resulting free amine was coupled with alkanoic acids followed by catalytic hydrogenolysis with Pd(OH)_2_ under H_2_ producing the final compounds **1**–**4** with varied lengths of the fatty acid acyl chain.

### Identification of a D‐galactosyl PI4P mimetic as the selective substrate of PIP5Ks

**Figure 1:**
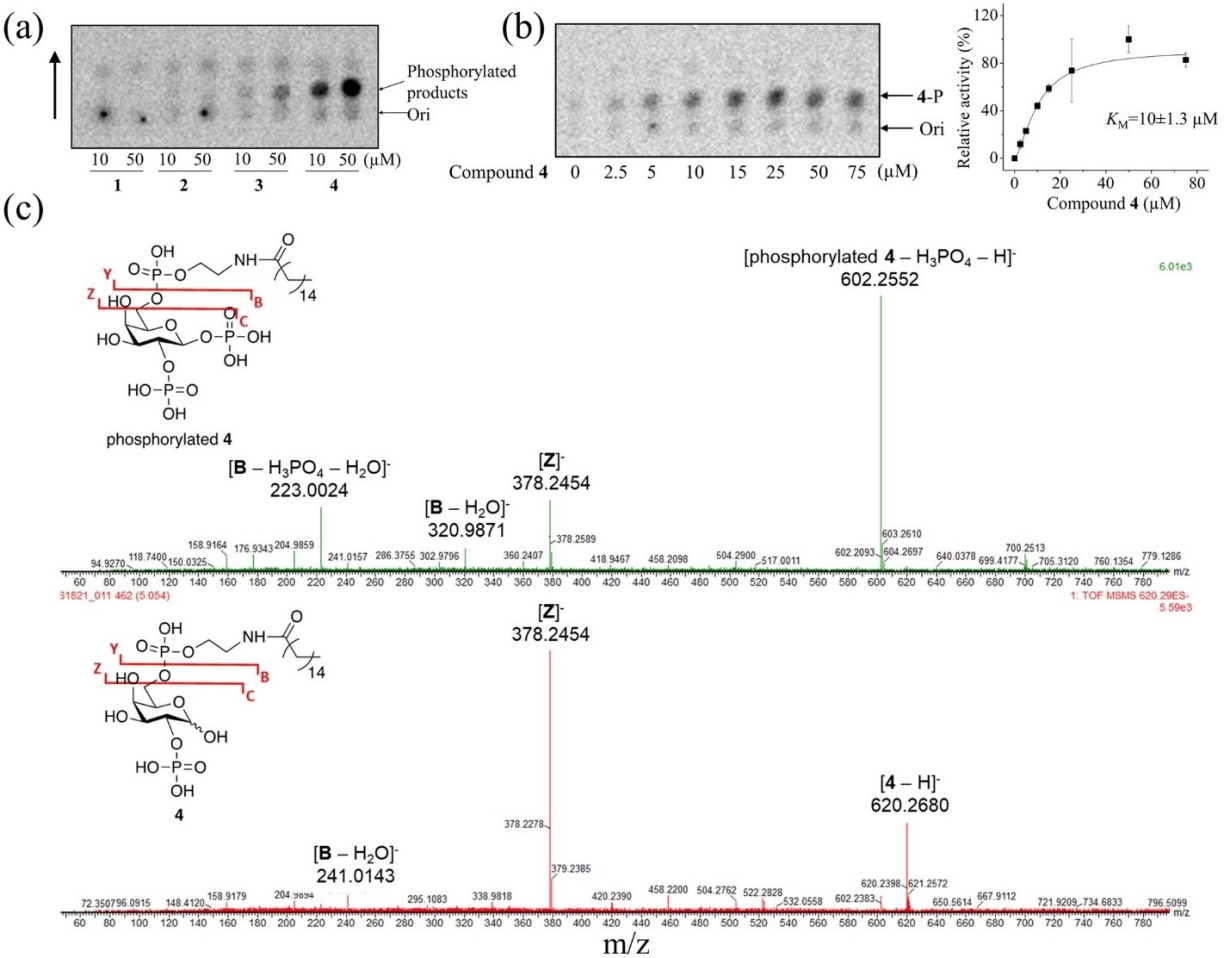
Phosphorylation of 2‐phosphate D‐galactosyl lysophospholids by zPIP5Kα. (a) TLC of the phosphorylation reactions of the PI4P mimetics (**1**–**4**) with varied length of fatty acyl chain as detected via radioactive ^32^P. Ori stands for the origin of the reaction mixture spotted on the TLC. The direction of TLC is indicated by the arrow. (b) Dose‐dependent phosphorylation of **4** by zPIP5Kα. **4**‐P stands for phosphorylated compound **4**. The *K*_M_ was estimated by curve fitting using the Hill model based on the intensities of ^32^P radioactivities of the product on the TLC plate. The error bars indicate the S.D. (n=2). (c) MS/MS fragmentation of phosphorylated **4** (*upper*) and **4** (*lower*) after separation by ion‐pair liquid chromatography (Figure S1). The proposed fragmentation patterns of compound 4 and its phosphorylated product are depicted.

With compounds **1**–**4** in hand, their interactions with PIP5K were tested, as PIP5K utilizes PI4P as the natural substrate. The compounds were incubated with the purified PIP5Kα from zebrafish (zPIP5Kα) in the presence of radioactive γ‐^32^P‐ATP and Mg^2+^. The lipophilic substances were then extracted by organic solvent and subjected to thin‐layer chromatography (TLC) analysis, followed by exposure to a storage phosphor screen and quantification by a phosphoimager. As shown in Figure 1a, significant amounts of phosphorylated species were detected only for **3** and **4**, with the phosphorylation of **4** much more prominent than that for **3**. The dose‐dependent study indicated that **4** is phosphorylated by zPIP5Kα with a *K*_M_ of 10±1.3 μM (Figure 1b).

In order to characterize the product, mass spectrometry analysis was carried out. With compound **4** as the substrate (621.26 Da), a newly formed double‐charged species with +80 Da molecular weight in the negative ion mode (349.61 Da) was detected in the reaction mixture after the treatment with zPIP5Kα, indicative of mono‐phosphorylation on a hydroxyl group (Figure S1A). No double or multiple phosphorylated products were detected. MS/MS fragmentation of the phosphorylated product separated in an ion‐pair liquid chromatography (Figure S1B), together with MS/MS fragmentation of **4** conducted under the same condition, supported a fragmentation pattern where the glycosyl phosphate bond at the anomeric position and the phosphodiester bond at C6 are broken, whereas the phosphoester bond at C2 is preserved in both **4** and its phosphorylated product (Figure 1c). These results indicate that the anomeric OH of **4** likely adopts the equatorial configuration in the enzyme binding site similar to the *myo*‐inositol 4‐phosphate of PI4P so that **4** is phosphorylated by PIP5K in the same manner as PI4P. Given that these D‐galactosyl lysophospholipids likely form micelles with different sizes, the dependence of reactivity of the PI4P mimetics on fatty acyl chain length, i.e., **4** has the highest reactivity compared to **1**–**3**, supports the notion that PIP5K's activity is membrane surface dependent and highly sensitive to membrane properties.^[19]^

**Figure 2:**
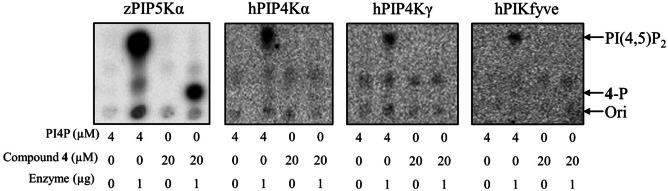
Comparison of PI4P and compound **4** phosphorylation by PIPKs. Phosphorylation of **4** by mPIP5Kγ is shown in Figure S4. zPIP5Kα exhibited a higher enzymatic activity towards PI4P than other PIPKs tested.

Next, we tested whether **4** could be phosphorylated by other members in the PIPK family, including mouse PIP5Kγ (mPIP5Kγ), zPIP5Kα, human PIP4Kα (hPIP4Kα), human PIP4Kγ (hPIP4Kγ), and human PIKfyve (hPIKfyve). Except for hPIKfyve which was expressed in mammalian cells with an *N*‐terminal FLAG tag, the other PIPKs were expressed in *E.coli* with an N‐terminal His‐tag (Figure S2). As shown in Figure 2, under the optimized experimental condition (Figure S3), although PI4P was phosphorylated by all the tested PIPKs, **4** was only robustly processed by PIP5Ks, including the mPIP5Kγ from mouse and zPIP5Kα from zebrafish, but not by PIP4Ks or PIKfyve under the same conditions, indicating that **4** is a selective substrate exhibiting a greater preference for PIP5K over the other two types of PIPKs than PI4P.

### Discovery of a D‐galactosyl lysophospholipid‐based PIPK inhibitor

**Figure 3:**
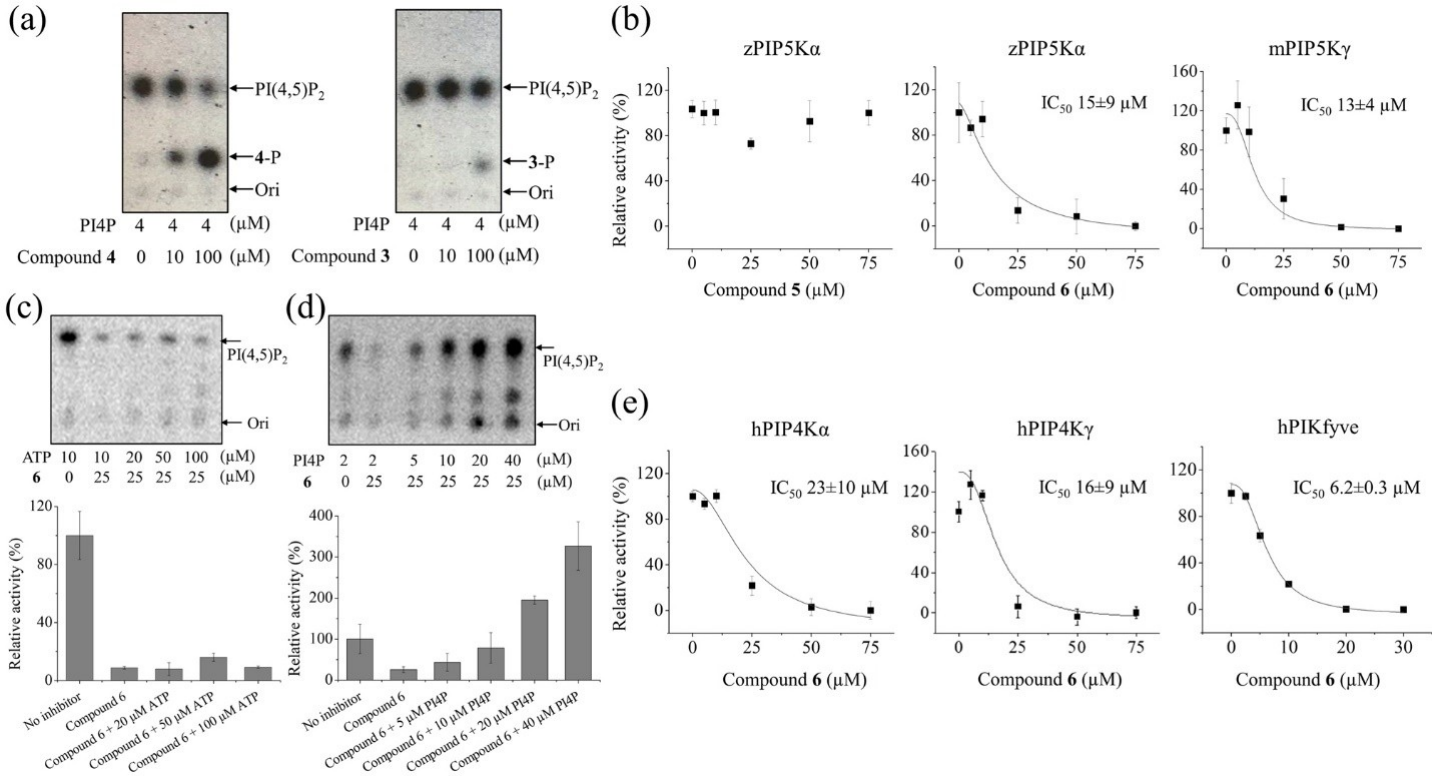
Discovery of a non‐ATP‐competitive pan‐PIPK inhibitor with preference toward PIKfyve. (a) Inhibition of zPIP5Kα by **3** and **4**. (b) Comparison of compound **4** derivatives (compounds **5** and **6**) on inhibition of PIP5Ks. IC_50_ values were estimated by curve fitting using the Hill model. (c) Inability of ATP to relief inhibition of zPIP5Kα by **6**. (d) Restoration of zPIP5Kα’s activity by addition of excessive amount of PI4P. (e) Inhibition of PIP4Ks and PIKfyve by **6**. The shown representative results are from one out of 3–4 independent experiments with three biological replicates for each data point. The error bars indicate the S.D. The representative TLC results of PIPK inhibition is shown in Figure S6.

As compounds **3** and **4** can be phosphorylated by zPIP5Kα, we expected that they can compete for the enzyme's active site with the natural substrate PI4P. Indeed, as shown in Figure 3a, **4** exhibited a dose‐dependent inhibition against PI4P phosphorylation by zPIP5Kα with concomitantly increased phosphorylation of **4**. In contrast, inhibition of zPIP5Kα by **3** was fairly weak even at the high concentration and phosphorylation of **3** was also much less than that of **4**. The positive correlation between the compound's reactivity and inhibition potency is consistent with the notion that the PI4P mimetics **3** and **4** compete with PI4P for the kinase active site. These results also suggest that the observed inhibition of PIP5K is unlikely due to the membrane disruption caused by the lysolipid or detergent effect, because compound **3** would otherwise be a more potent inhibitor than **4** as a lysophospholipid with a shorter fatty acyl chain perturbs membrane more than the one with a longer aliphatic chain.^[20]^

**Scheme 5:**
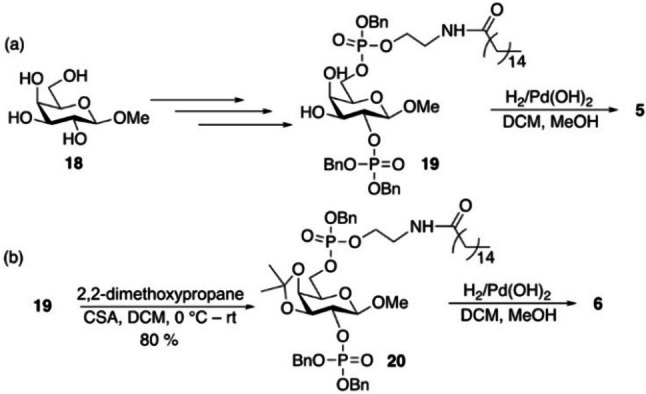
Syntheses of (a) methyl galactoside **5** and (b) **6**.

Next, we aimed to transform compound **4** into a competitive inhibitor of PIPKs. We first designed compound **5**, which was synthesized starting from methyl‐β‐D‐galactopyranoside **18** following a similar synthetic strategy as that for the preparation of **4** leading to methyl‐galactoside **19** (Scheme 5a and Scheme S2). Hydrogenolysis of the benzyl groups in **19** produced **5**, which contained a methoxy group at the anomeric position of galactoside, thus could not be phosphorylated at the anomeric center. Incubation of **5** with zPIP5Kα and γ‐^32^P‐ATP and Mg^2+^ under the same condition as in phosphorylation of **4** did not lead to any ^32^P labeled products. The lack of phosphorylation of **5** supports the notion that 1‐*O* of **4** is phosphorylated by zPIP5Kα. However, **5** did not exhibit significant inhibitory effects toward zPIP5Kα either (Figure 3b).

By examining the structural model of PI4P‐bound zPIP5Kα (Figure S5),[^1e^, ^21^] we noticed that there is space between the loop containing the PIPK signature “DLKGS” motif near the active site and 2‐OH/3‐OH of *myo*‐inositol of PI4P. We then designed compound **6**, which contains an isopropyl group linking 3‐OH and 4‐OH of D‐galactose (equivalent to 2‐OH and 3‐OH of *myo*‐inositol, Scheme 2a) potentially filling up this space for a better interaction with the enzyme. For synthesis of **6**, the 3,4‐diols of compound **19** were functionalized by the isopropylidene group (Scheme 5b), which was then followed by the hydrogenolysis of the benzyl groups to produce **6**.

**Figure 4:**
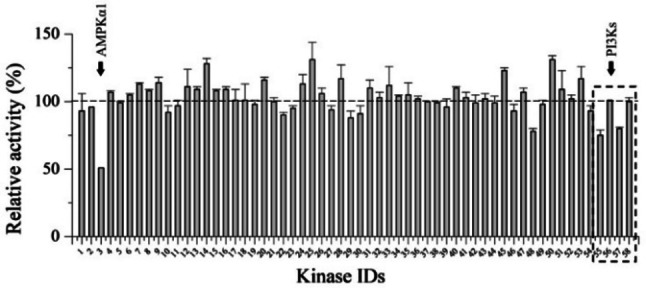
Activity‐based screening of 58 human kinases. The activity of each kinase in the presence of 20 μM of compound **6** is expressed as the percentage of the activity of the control group (DMSO). AMPKα1 and PI3Ks are indicated by arrows. Four and two biological replicates were included in the control group and the inhibitor group, respectively. The kinase ID list and the activity data are shown in Figure S7.

Remarkably, compound **6** was found to be a zPIP5Kα inhibitor with the apparent IC_50_ of ∼15 μM (Figures 3b&S6). mPIP5Kγ was also similarly inhibited by **6** (Figure 3b). As the inhibition by **6** can only be relieved by adding the excessive amount of PI4P, but not by adding ATP (Figures 3c&d), we concluded that **6** is a non‐ATP‐competitive inhibitor of zPIP5Kα. We next examined the activities of **6** against other PIPKs and it turned out that **6** inhibited all the tested PIPKs, including hPIP4Kα, hPIP4Kγ, and hPIKfyve (Figure 3e). hPIKfyve appeared to be most sensitive to **6** and the apparent IC_50_ is more than two times smaller than that for zPIP5Kα (6.2 vs. 15 μM). Based on these results, we concluded that **6** is a pan‐PIPK inhibitor with a preference toward PIKfyve. To examine whether compound **6** inhibits kinases other than PIPKs, we conducted an activity‐based kinase screening (KinaseProfiler^TM^ provided by Eurofins Inc.), which includes 58 representative human kinases from major kinase families. As shown in Figure 4 and Figure S7, compound **6** at 20 μM only reduced the activity of 5′ AMP‐activated protein kinase α1 (AMPKα1) by 50 % whereas exhibiting no or very modest inhibition against other tested kinases, demonstrating a high selectivity. As **6** does not compete with ATP, inhibition of AMPK may be due to an allosteric effect. Interestingly, **6** modestly inhibited (by 20–25 %) two but not all the four tested PI3K isoforms, suggesting that the current strategy for PIPK inhibition might be applied to develop isoform‐specific inhibitor of PI3Ks, which hold great potential to treat cancers.^[22]^

**Figure 5:**
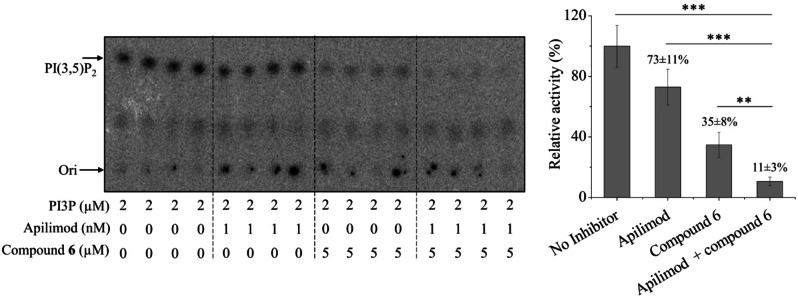
Inhibition of hPIKfyve by Apilimod and compound **6**. The shown representative results are from one out of two independent experiments with four replicates for each data point shown. The error bars indicate the S.D. Statistical analysis was conducted using Student's *t*‐test. **: P<0.01; ***: P<0.001.

PIKfyve is a drug target for a variety of diseases, in particular SARS‐CoV‐2 infection.[^10b^, ^11^] Apilimod is a selective PIKfyve inhibitor under clinical evaluation against SARS‐CoV‐2 and ALS, but this compound has a fairly low bioavailability which may account for the low efficacy in prior clinical trials.^[23]^ As **6** targets the lipid substrate binding site, we next examined whether its inhibitory effect can be combined with Apilimod which is known to target the ATP binding site (Figure S8). As shown in Figure 5, when Apilimod at 1 nM was combined with **6** at 5 μM, an additive inhibitory effect was observed, indicating that **6** can significantly potentiate the effectiveness of Apilimod as a PIKfyve inhibitor.

### Inhibition of transferrin endocytosis by compound 6

**Figure 6:**
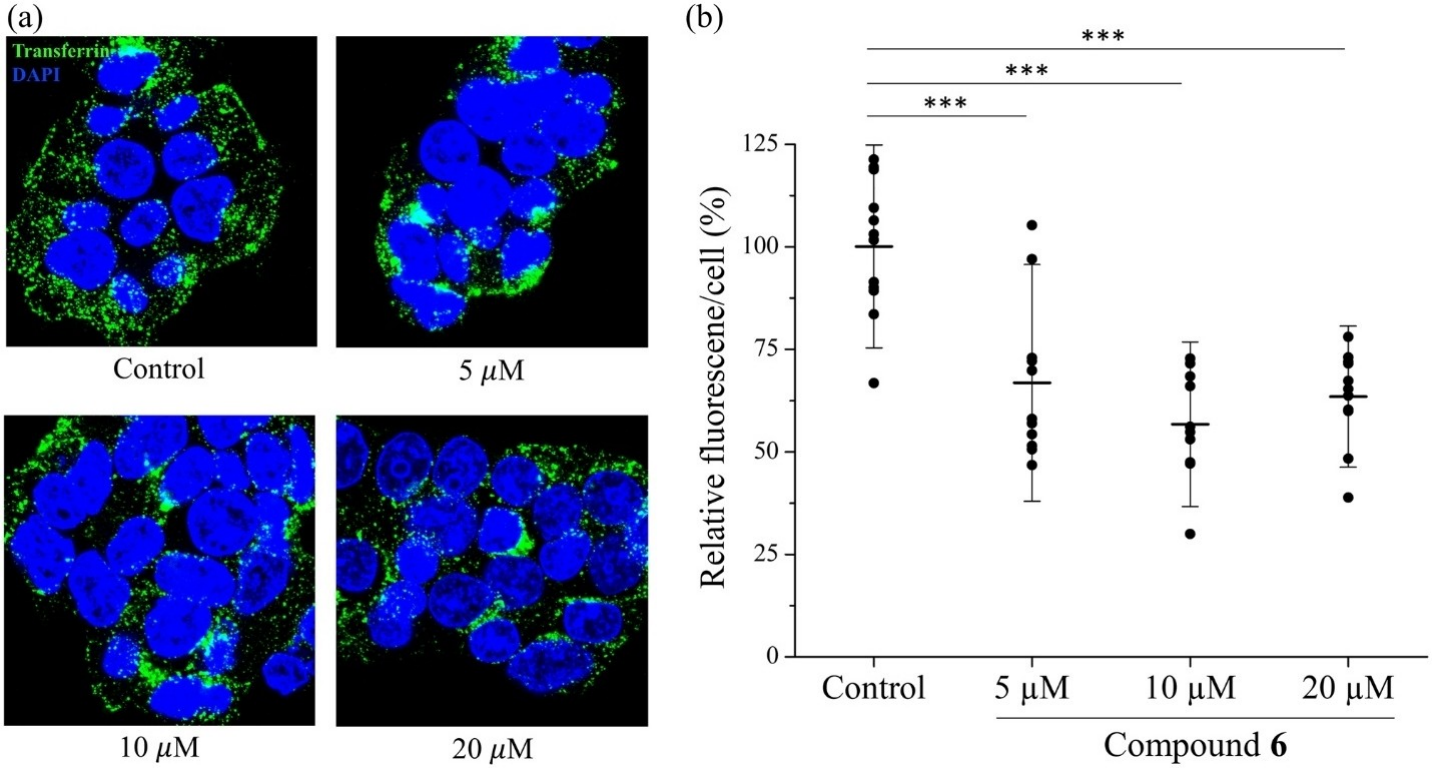
Inhibition of transferrin endocytosis by compound **6**. (a) Representative confocal images of HEK293T cells after incubation with Alexa 488 conjugated transferrin (green) in the absence or presence of indicated concentrations of compound **6**. The nuclei were stained with DAPI (blue). (b) Quantification of transferrin endocytosis. The green fluorescence intensity per cell is expressed as the percentage of that of the cells in the control group. Each data point represents the average fluorescence intensity per cell in one image and 10–12 images (a total of ∼200 cells) were analyzed for each condition. Statistical analysis was conducted using Student's *t*‐test. ***: P<0.001.

Lastly, we tested the biological activity of **6** in a transferrin endocytosis assay. Transferrin is an iron binding protein circulating in blood stream as the major carrier of ferric ions. It enters cells to deliver iron through receptor‐mediated endocytosis which requires the activity of PIP5K to generate PI(4,5)P_2_. PIP4Ks and PIKfyve are also involved in endocytosis by regulating intracellular vesicle trafficking.^[24]^ To examine the effects of **6** on endocytosis, HEK293T cells were incubated with **6** for two hours and then Alexa 488 conjugated transferrin (with green fluorescence) was added to cells to initiate endocytosis. As shown in Figure 6, **6** did not cause significant changes in morphology or cell membrane integrity but significantly reduced the intensity of the green fluorescence in cells in a dose‐dependent manner. At 10 μM, **6** reduced transferrin internalization by nearly 50 %. The small uptick of endocytosis rate at 20 μM may be attributed to the detergent nature of **6**, as it has been reported that detergents may increase endocytosis.^[25]^ The strong inhibition of transferrin endocytosis by **6** suggests that it may exert its PIPK inhibition activity in cells and additional studies to clarify the exact inhibition mechanism is warranted in future work.

## Discussion

PIPKs are promising drug targets against a variety of human diseases, but most of the reported PIPK inhibitors fall into the category of ATP competitors.[^7c^, ^9^, ^12^] Targeting the non‐ATP binding sites using substrate analogs as non‐ATP‐competitive inhibitors is an attractive strategy to circumvent the concerns associated with ATP‐competitive inhibitors. Two compounds identified in large scale screenings were reported to be the lipid substrate competitive inhibitors for PIP5Kα and PIP4Kγ, respectively,^[26]^ but there is no similarity in chemistry between the headgroups of the natural phospholipid substrates and the reported compounds which contain three or four aromatic rings with extremely low solubility. To the best of our knowledge, the compounds discovered in this work are the first to target the lipid substrate binding sites of PIPKs through rational design.

Using D‐galactose derivatives to mimic the *myo*‐inositol moiety of phosphoinositides has two advantages over directly derivatizing *myo*‐inositol. Firstly, as the phosphoinositides are critically involved in cell signaling, the cells are highly sensitive to the concentration changes of these low‐abundance lipids. It is conceivable that a synthetic *myo*‐inositol‐based phosphoinositide analog and/or its metabolic products may interfere with cell signaling processes, causing unexpected adverse effects. As galactose is one of the major intracellular metabolites, the galactose derivative derived from the synthesized compound is less likely to severely disrupt cellular functions when applied at a low micromolar concentration. Secondly, from the perspective of synthesis, differentiating the almost identical hydroxyl groups of *myo*‐inositol is much more challenging than galactose functionalization. Indeed, synthesis of *myo*‐inositol derivatives often takes more than 20 steps,^[27]^ whereas producing D‐galactosyl lysophospholipids in this work only took 10 or fewer steps from the commercially available compounds. D‐Galactose based phosphonate analogs were reported to suppress phosphatidylinositol biosynthesis in mycobacteria, but those compounds were shown not to be the substrates of the target enzymes even at millimolar concentration.^[28]^ In contrast, in this work we show that the D‐galactose‐based PI4P mimetic **4** can be robustly phosphorylated by PIP5Ks with a *K*_M_ of 10 μM for zPIP5Kα (Figure 1). It is for the first time that a D‐galactose derivative is shown to enter the active site and be processed by a phosphoinositide‐processing enzyme.

It is interesting that, although PI4P was processed by all the PIPK family members in the *in vitro* kinase assay (Figure 3), which can be attributed to substrate promiscuity of PIPKs,^[29]^ compound **4** exhibited a strict selectivity toward PIP5K. The small but substantial structural differences between **4** and PI4P in their headgroups likely account for the high selectivity of the former, allowing it to better distinguish the substrate binding pockets of different PIPK family members. While **4** is a selective substrate of PIP5K, it is unexpected that **6**, which is a derivative of **4**, turned out to be a pan‐PIPK inhibitor (Figures 3b&e). A non‐specific detergent effect on kinase activity can be excluded because the structurally similar compound **5** showed no effect on zPIP5Kα’s kinase activity at concentrations up to 75 μM (Figure 3b). The ATP and PI4P competition assays further indicated that **6** is a non‐ATP‐competitive inhibitor, which targets the lipid substrate binding site (Figures 3c&d). Given that the lipid‐facing substrate binding pocket of PIPKs is highly dynamic, probably due to the lipid sensing function of the activation loop,[^19b^, ^19c^] we postulate that the substrate binding pockets of different PIPKs may be flexible enough to accommodate **6**, but only when bound with PIP5Ks can **4** adopt a productive orientation to allow phosphorylation to occur. Nevertheless, **6** is a highly selective PIPK inhibitor, which is likely associated with its activity as an endocytosis blocker (Figures 4&6).

## Conclusion

In this work, we demonstrate that the rationally designed D‐galactosyl lysophospholipids are promising candidates for developing potent and selective non‐ATP‐competitive inhibitors of PIPKs. Generating type‐specific PIPK inhibitor and eliminating the detergent‐like properties will make these compounds more effective as research tools and potential therapeutic reagents. The strategy of design and synthesis of D‐galactose‐based phosphoinositide mimetics presented in this work is likely applicable to the study of other phosphoinositide‐processing enzymes, including PI3Ks. For future applications in biological systems, we envision that these lipidic compounds will be incorporated into liposomes for delivery *via* membrane fusion and/or endocytosis with the target cells. The compounds will then diffuse within the endomembrane system through the highly dynamic lipid transport mechanism to reach the targets at the lipid‐water interface. Using liposomes to deliver hydrophobic compounds has been applied to multiple drugs, including Amphotericin B,^[30]^ paclitaxel,^[31]^ and verteporfin.^[32]^

## Experimental Section

**Materials and General Methods**: Chemicals were reagent grade as supplied. Analytical thin‐layer chromatography was performed using silica gel 60 F254 glass plates. Compound spots were visualized by UV light (254 nm) and by staining with a yellow solution containing Ce(NH_4_)_2_(NO_3_)_6_ and (NH_4_)_6_Mo_7_O_24_ ⋅ 4H_2_O in 6 % H_2_SO_4_. Flash column chromatography was performed on silica gel 60 (230‐400 Mesh). NMR spectra were recorded on Agilent 500 MHz DDR2 NMR spectrometer and referenced using Me_4_Si (0 ppm), residual CHCl_3_ (d ^1^H NMR 7.26 ppm, ^13^C NMR 77.0 ppm). All optical rotations were measured at 25 °C using the sodium D line. High resolution mass spectra were recorded on a Waters Xevo G2‐XS QTof quadrupole mass spectrometer.

**Mass spectrometry**: The phosphorylation reactions were carried out in a 1.5 mL microcentrifuge tube with 50 μM **4**, 50 μM ATP, 0.1 μM zPIP5Kα in the reaction buffer (100 mM Tris‐HCl pH 8.0, 5 mM EGTA, 10 mM MgCl_2_). After incubated at room temperature for 4 h or overnight, the protein was precipitated by adding acetonitrile. Then, acetonitrile was evaporated under N_2_ stream and water was removed by a lyophilizer. The reversed phase ion‐pairing UPLC/ESI/MS/MS analysis of the reaction products was performed on a waters Xevo G2‐XS QTof quadrupole mass spectrometer with an ESI probe. Mass spectrometry was operated in the negative ion mode with a capillary voltage of 4000 V and at a source temperature of 600 °C. The UHPLC system consisted of an Agilent 1290 binary pump equipped with an autosampler at 10 °C. The injection volume was 5 μL. The reaction products were chromatographically resolved in an ACQUITY UPLC BEH C18 column, 1.7 μM, 2.1×150 mm (Waters, part# 186002353). Mobile phase A was 8.0 mM DMHA (dimethylhexylamine) and 2.2 mM Acetic acid in water, pH 9.0. Mobile phase B was methanol. The gradient gradually increased from 0 % to 40 % of mobile phase B for 10 min and then decreased to 0 % of mobile phase B for additional 5 min. The solvent flow rate was 0.3 mL/min and column temperature were kept at 40 °C.

**Genes and constructs**: The synthesized gene of zebrafish PIP5Kα with optimized codon was inserted in a pET41b vector with a C‐terminal His‐tag. The cDNAs for human PIP4Kα, human PIP4Kγ, mouse PIP5Kγ isoform 1, and human PIKfyve were purchased from Horizon Discovery with the catalog numbers of MHS6278‐202806784 (GenBank: BC018034.1), MHS1010‐202807181 (GenBank: BC028596.2), MMM1013‐202768039 (GenBank: BC019138.1), and OHS5893‐202503964 (GenBank: BC172527.1), respectively. The genes encoding hPIP4Kα, hPIP4Kγ, and mPIP5Kγ were cloned in a modified pET17 vector with an *N*‐terminal His‐tag for expression in *E. coli*. The gene of hPIKfyve was cloned in the pcDNA3.1 vector with an *N*‐terminal FLAG tag for expression in mammalian cells.

**Protein expression and purification**: hPIP4Kα and mPIP4Kγ were expressed in Rosetta‐2 (DE3) cells (Millipore). After the OD_600_ reached around 0.5, the cells growing in Luria‐Bertani (LB) medium were induced with 0.1 mM isopropyl‐β‐D‐thiogalactoside (IPTG). The cells were left to grow overnight at room temperature, harvested and re‐suspended in the lysis buffer containing 20 mM Tris pH 8.0, 300 mM NaCl, 5 % glycerol, and EDTA‐free protease inhibitor cocktail. After lysis by sonication, the cell lysate was applied to centrifugation (20,000×*g* at 4 °C) for 40 minutes. The supernatant was collected and loaded on a Ni‐NTA column (Qiagen) for binding at 4 °C for 4 h. After washing with the washing buffer (20 mM Tris pH 8.0, 300 mM NaCl, 5 % glycerol, and 10 mM imidazole) for four times, the protein was eluted by the elution buffer (washing buffer plus 200 mM imidazole). The protein was further purified using a Superdex 200 Increase 10/300 GL column (GE healthcare) equilibrated with the running buffer containing 10 mM HEPES pH 7.3, 300 mM NaCl, and 5 % glycerol. zPIP5K**α** was overexpressed in BL21‐CodonPlus®(DE3)‐RIL cells (Stratagene) in LB medium. After the OD_600_ reached 0.3, the cells were induced with 0.1 mM IPTG and left to grow overnight at room temperature. The purification procedure was the same as that for PIP4Ks, except that 0.5 % Triton X‐100 (American Bioanalytical) was added to the cell lysate and Talon metal affinity column (Clontech) was used for purification. 0.01 % Triton X‐100 was included in the running buffer for size‐exclusion chromatography.

hPIKfyve was expressed in Expi293F cells (Thermo Fisher) transiently transfected with the pcDNA3.1 plasmid harboring the DNA encoding the full length human PIKfyve with an *N*‐terminal FLAG tag. The cells were cultured in EXPi293 Expression Medium at 8 % CO_2_ and 37 °C with shaking at 130 rpm. When the cells reached a density of 3–5×10^6^ cells/mL (100 mL culture in 500 mL flask), the cells were split and diluted to 1.5×10^6^ cells/mL and then cultured overnight. 100 μg of PIKfyve plasmid was added to 6 mL of Opti‐MEM I reduced serum medium and mixed by swirling, while 320 μL of EXPifectamine 293 was mixed with 5.6 mL of Opti‐MEM I reduced serum medium. After incubated at room temperature for 5 min, the two solutions were combined, incubated at room temperature for 20 min, and slowly added to cell culture. One day after the transfection (18–22 h), 0.6 mL of ExpiFectamine 293 transfection enhancer 1 and 6 mL of ExpiFectamine 293 transfection enhancer 2 were added to the cell culture flask. The cells were harvested four days after transfection and re‐suspended in lysis buffer containing 50 mM Tris, 300 mM NaCl, pH 7.4, and 5 % glycerol. Then, the cell suspension was applied to sonication for 15 min on ice. The anti‐FLAG M2 affinity gel (MilliporeSigma, Cat#A2220) was balanced with the TBS buffer (50 mM Tris, 150 mM NaCl, pH 7.4) after washing with 0.1 M glycine buffer, pH 3.5 for three times. The cell lysate after sonication was applied to centrifugation (11,000 rpm at 4 °C) for 15 min and the resulting supernatant was mixed with the prepared anti‐flag M2 beads. After incubated at 4 °C for 2 h with gentle shaking, the beads were washed with the purification buffer (50 mM Tris, 300 mM NaCl, pH 7.4, 5 % glycerol) for five times. Then, the protein was slowly eluted with three column volumes of elution buffer containing the purification buffer supplemented with 100 μg/ml FLAG peptide (MedChemExpress, Cat#HY−P0223).

**Kinase assay**: The following components were included in one reaction (50 μL): 0.25‐1 μg of purified kinase, 100 mM Tris‐HCl (pH 8.0), 5 mM EGTA, 10 mM MgCl_2_, 50 μM ATP with 1 μCi [γ‐^32^P] ATP (PerkinElmer), and 2 μM diC16‐PIP (diC16‐PI4P for PIP5K, diC16‐PI5P for PIP4K, and diC16‐PI3P for PIKfyve) (Echelon Biosciences Inc.). The reaction was performed at room temperature for 1 h and then quenched by the addition of lipid extraction solution containing chloroform, methanol, and HCl with a volume ratio of 3.3 : 3.7:0.1, as well as bovine follicular fluid (10 mg/ml). After vertexing for 20 s, the sample was centrifuged at 6000 rpm for 2 min. The aqueous layer was removed, and the lower organic layer was spotted on TLC plate (6 μL) and separated by a developing solvent (water, acetic acid, methanol, acetone, chloroform with a volume ratio of 1 : 1.3 : 1.7 : 2.1 : 4.5). The reaction products were quantified by a Storm 820 PhosphorImager (GE). For the inhibition assay, the inhibitors were added to the reaction mixture at the indicated concentrations. To optimize reaction time for each kinase, time course experiments were conducted under the same experimental conditions and the reactions were terminated at the indicated time. The results are shown in Figure S3.

**Immunoblotting**: All the samples were heated in the SDS sample loading buffer at 96 °C for 6 min, and then applied to SDS‐PAGE and Western blot. hPIP4Kα, hPIP4Kγ, zPIP5Kα and mPIP5Kγ proteins were detected by using an anti‐His Tag antibody at 1 : 6000 dilution (Invitrogen, Cat#37‐2900) and horseradish peroxidase (HRP)‐conjugated anti‐mouse IgG at 1 : 7000 dilution (Cell Signaling Technology, Cat#7076S). FLAG‐hPIKfyve was detected by using rat anti‐FLAG antibody at 1 : 4000 dilution (Stratagene, Cat#200474‐21) and HRP‐conjugated anti‐rat IgG at 1 : 7000 dilution (Cell Signaling Technology, Cat#7077S). After reacting with the detection reagent (Cytiva, RPN2232), the images were taken using a Bio‐Rad ChemiDoc Imaging System.

**Kinase activity screening**: The kinase screening was conducted using the activity‐based KinaseProfiler^TM^ technology provided by Eurofins Inc. Compound **6** dissolved in DMSO was added to the samples at the final concentration of 20 μM. A total of 58 human kinases (Diversity Panel) were tested. For each kinase, four replicates were included in the control group and two in the inhibitor group.

**Transferrin endocytosis assay**: HEK293T cells grown on sterile glass coverslips in Dulbecco's modified eagle medium (DMEM, Invitrogen, Cat#11965092) supplemented with 10 % (v/v) fetal bovine serum (Invitrogen, Cat#10082147) were pretreated with compound **6** at indicated concentrations for 2 h, and then Alexa 488 conjugated transferrin (25 μg/ml, final concentration, Thermo Fisher Scientific, Cat#T13342) was added to initiate endocytosis at 37 °C for 12 min. The cells were washed five times with ice‐cold Dulbecco's phosphate‐buffered saline (DPBS) and fixed for 10 min at room temperature using 4 % formaldehyde. Coverslips were then mounted on slides with fluoroshield mounting medium with 4’,6‐diamidino‐2‐phenylindole (DAPI, Abcam, Cat# ab104139). Samples were analyzed using a Zeiss Axio fluorescence microscope.

**Statistical analysis**: Statistical analysis was conducted using the Student's t‐test.

## Author Contribution

J.H. and X.H. formulated the project. M.S., C.Z., D.S., and C.Y. conducted experiments. M.S., C.Y., D.P., J.H., and X.H. analyzed data. M.S., J.H., and X.H. wrote the manuscript.

## Funding

This work was supported by NIH grants GM140931 (to J.H.) and GM72667 (to X.H.).

## Conflict of interest

The authors declare no competing interests associated with the manuscript.

1

## Supporting information

As a service to our authors and readers, this journal provides supporting information supplied by the authors. Such materials are peer reviewed and may be re‐organized for online delivery, but are not copy‐edited or typeset. Technical support issues arising from supporting information (other than missing files) should be addressed to the authors.

Supporting Information

## Data Availability

The data that support the findings of this study are available in the supplementary material of this article.
