# Supplementary material for: Rational Design and Synthesis of D‐galactosyl Lysophospholipids as Selective Substrates and non‐ATP‐competitive Inhibitors of Phosphatidylinositol Phosphate Kinases
Source: Chemistry. 2022 Nov 24;29(2):e202202083. doi: 10.1002/chem.202202083 (PMC10099810; doi:10.1002/chem.202202083)
Supplement: Supplementary file 1 — Supporting Information [file CHEM-29-0-s001.pdf]

Chemistry–A European Journal

Supporting Information

Rational Design and Synthesis of D-galactosyl Lysophospholipids as Selective Substrates and non-ATP-competitive Inhibitors of Phosphatidylinositol Phosphate Kinases

Mengxia Sun, Chi Zhang, Dexin Sui, Canchai Yang, Dohun Pyeon, Xuefei Huang,* and Jian Hu*

Table of Contents

PART I. Supplementary Figures **S3**

Figure S1 | Phosphorylation of compound **4** by zPIP5Kα S3

Figure S2 | Purification of PIPKs S4

Figure S3 | The time courses of the phosphorylation reactions catalyzed by the PIPKs S5

Figure S4 | Phosphorylation of **4** by mPIP5Kγ S6

Figure S5 | Structural model of zPIP5Kα with bound ATP and PI4P S7

Figure S6 | TLC results of PIPK inhibition by compound **5** and compound **6** S8

Figure S7 | Kinase screening results using the KinaseProfiler™ technology provided by Eurofins Inc S9

Figure S8 | Inhibition of hPIKfyve by Apilimod S11

PART II. General Procedures **S12**

PART II. Product Preparation and Characterization Data **S14**

PART III. NMR Spectra **S25**

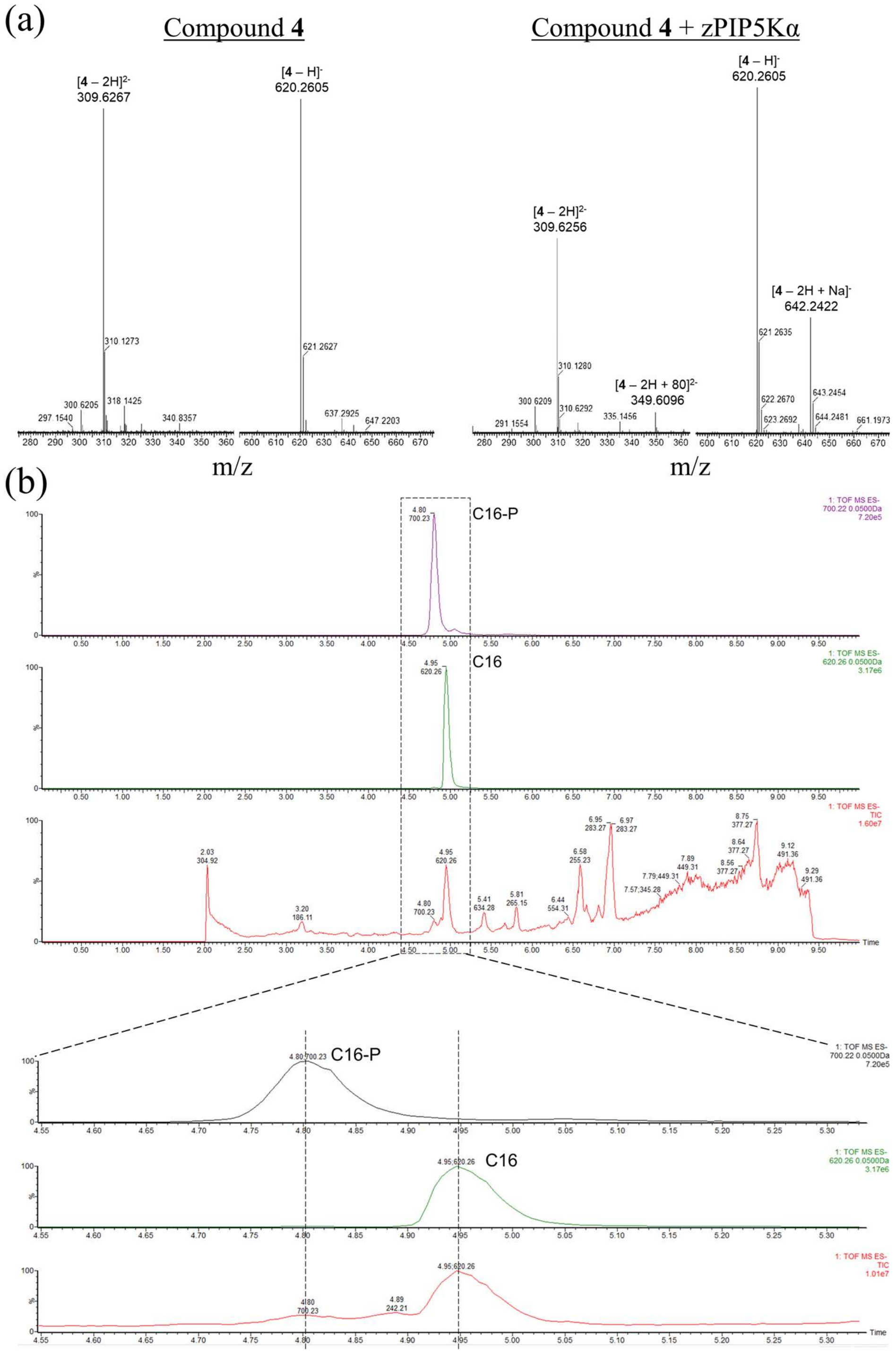

Figure S1. Phosphorylation of compound **4** by zPIP5Kα. **(a)** Identification of phosphorylated compound 4 in reaction mixture. The MS spectra of **4** before (*left*) and after (*right*) reaction with zPIP5Kα. The double-charged species (349.6096 Da) corresponds to the phosphorylated compound **4**. **(b)** Separation of **4** phosphorylation product using ion-pair liquid chromatography. Upper panel: Chromatography profiles of reaction mixture (*bottom*), the compound **4** with the MW of 620.26 Da (*middle*), and the mono-phosphorylated **4** with the MW of 700.23 Da *(top)*. Lower panel: Zoomed-in view of the highlighted regions.

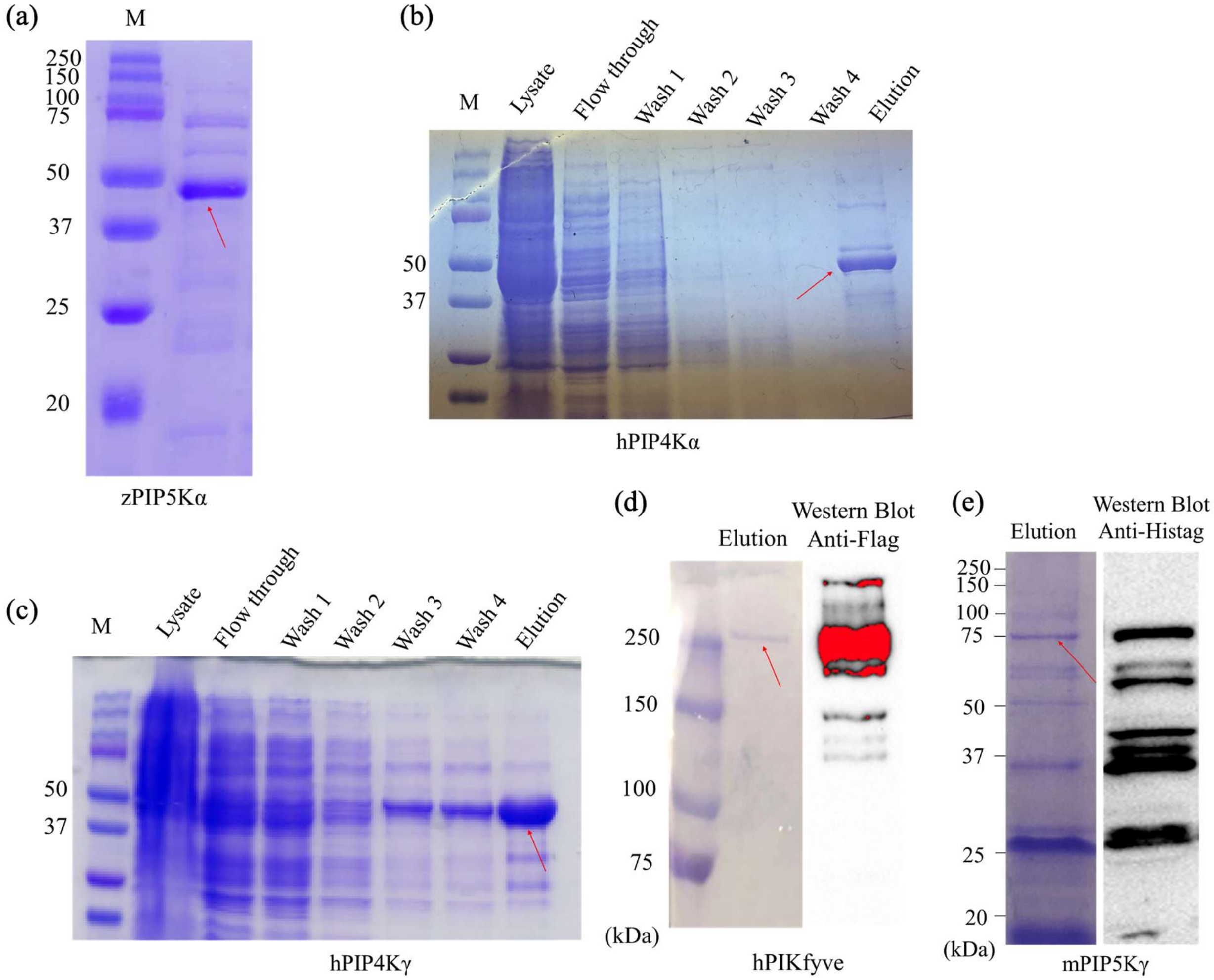

Figure S2. Purification of PIPKs. (a) zPIP5Kα. (b) hPIP4Kα. (c) hPIPK4Kγ. (d) hPIKfyve. (e) mPIP5Kγ. Except for N-flag-hPIKfyve expressed in HEK293F cells, the other histagged PIPKs were expressed in *E. coli*. hPIKfyve is confirmed by Western blot using an anti-Flag antibody. mPIP5Kγ is confirmed by Western blot using an anti-histag antibody with severe degradation due to instability of the purified protein.

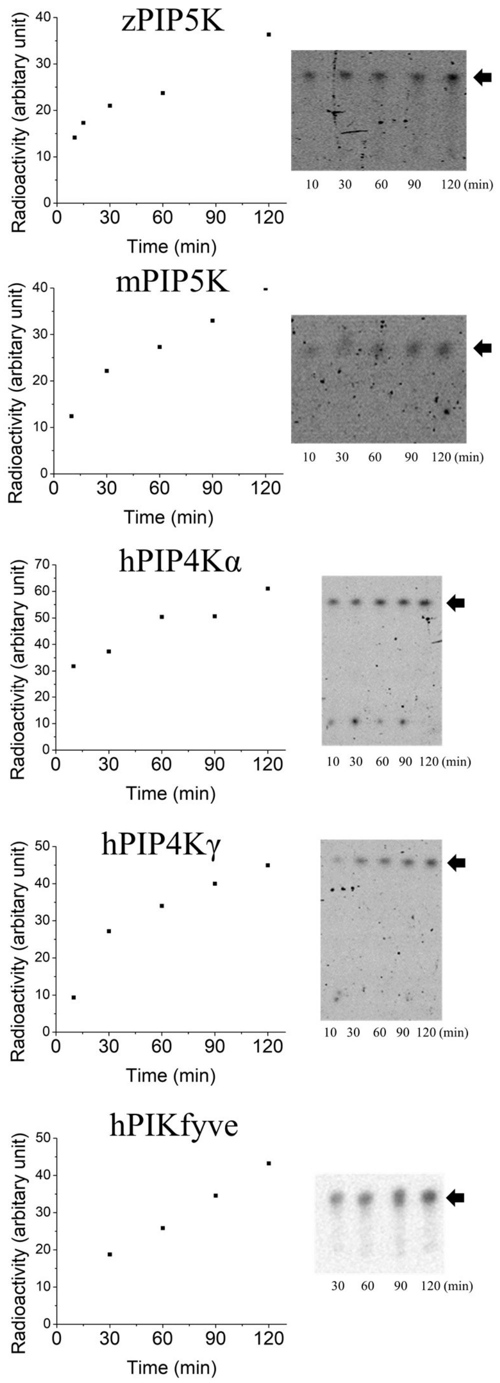

Figure S3. The time courses of the phosphorylation reactions catalyzed by the PIPKs. The arrows indicate the phosphorylated products, which were quantified by a Storm 820 PhosphorImager**.**

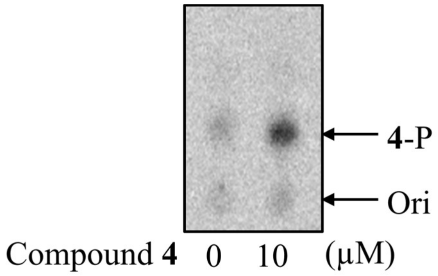

Figure S4. Phosphorylation of **4** by mPIP5Kγ. Purified mouse PIP5Kγ and compound **4** at the indicated concentration were mixed in the reaction buffer (100 mM Tris-HCl, pH 8.0, 5 mM EGTA, 10 mM $MgCl_2$, and 2 μM diC16-PI4P). The reaction was initiated by adding 50 μM ATP plus 1 μCi [γ-^{32}P] ATP (prepared in the reaction buffer). See more details in Experimental Section.

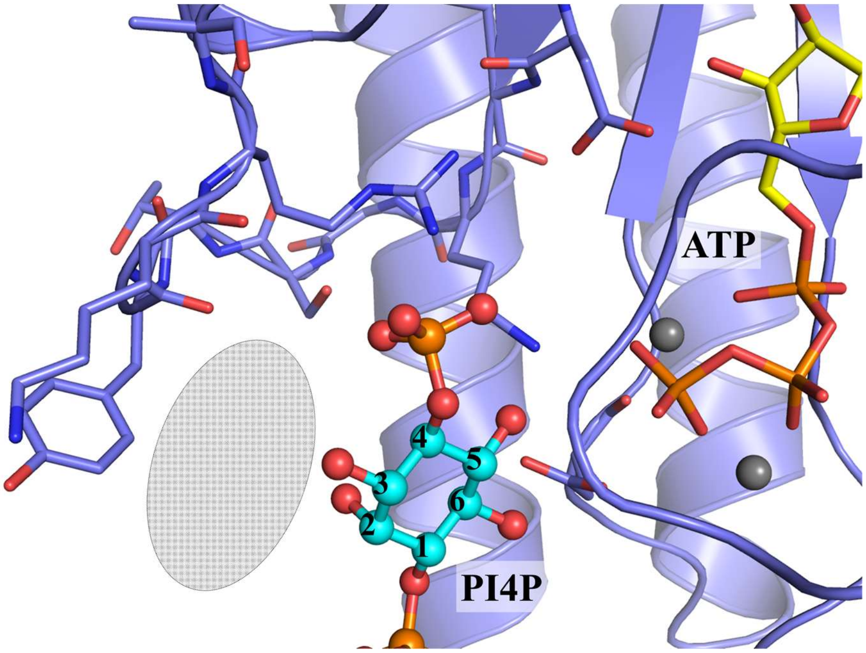

Figure S5. Structural model of zPIP5Kα with bound ATP and PI4P. The shaded area indicates a plausible cavity between PI4P and the loop containing the "DLKGS" motif conserved in the PIPK family. The model was built based on the crystal structure of the ATP-bound zPIP5Kα (PDB entry: 6CMW). PI4P was modeled based on previous mutagenesis studies and structural comparison with other eukaryotic kinases.

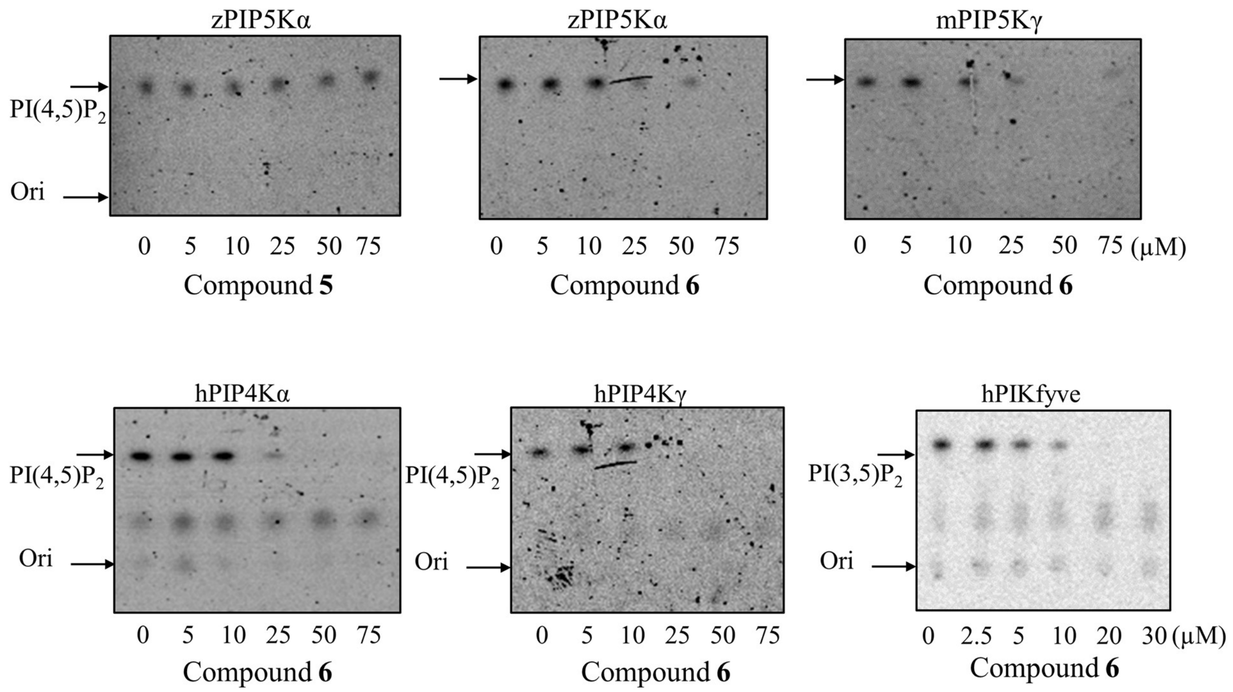

Figure S6. Representative TLC results of PIPK inhibition by compound **5** and compound **6**.

IDs	Kinases	Relative Activity (%)	S.D. (%)
1	Abl(h)	93	13
2	ALK(h)	96	0
3	AMPKα1(h)	51	0
4	ASK1(h)	107	1
5	Aurora-A(h)	99	1
6	CaMKI(h)	105	1
7	CDK1/cyclinB(h)	113	1
8	CDK2/cyclinA(h)	108	1
9	CDK6/cyclinD3(h)	114	4
10	CDK7/cyclinH/MAT1(h)	92	5
11	CDK9/cyclin T1(h)	97	4
12	CHK1(h)	111	13
13	CK1γ1(h)	109	2
14	CK2α2(h)	128	4
15	c-RAF(h)	108	1
16	DRAK1(h)	109	2
17	eEF-2K(h)	101	8
18	EGFR(h)	101	12
19	EphA5(h)	98	3
20	EphB4(h)	116	2
21	Fyn(h)	100	3
22	GSK3β(h)	90	2
23	IGF-1R(h)	95	2
24	IKKα(h)	113	7
25	IRAK4(h)	131	13
26	JAK2(h)	106	4
27	KDR(h)	94	3
28	LOK(h)	117	10
29	Lyn(h)	88	5
30	MAPKAP-K2(h)	91	6
31	MEK1(h)	110	6
32	MLK1(h)	103	4
33	Mnk2(h)	112	14
34	MSK2(h)	104	1
35	MST1(h)	105	9
36	mTOR(h)	102	2
37	NEK2(h)	100	0
38	p70S6K(h)	99	1
39	PAK2(h)	96	6
40	PDGFRβ(h)	110	1
41	Pim-1(h)	103	4
42	PKA(h)	99	6

43	PKBα(h)	102	4
44	PKCα(h)	99	5
45	PKCθ(h)	123	2
46	PKG1α(h)	93	5
47	Plk3(h)	107	3
48	PRAK(h)	78	2
49	ROCK-I(h)	98	3
50	Rse(h)	131	3
51	Rsk1(h)	109	14
52	SAPK2a(h)	102	3
53	SRPK1(h)	117	9
54	TAK1(h)	93	4
55	PI3 Kinase (p110b/p85a)(h)	75	4
56	PI3 Kinase (p120g)(h)	101	0
57	PI3 Kinase (p110d/p85a)(h)	80	1
58	PI3 Kinase (p110a/p85a)(h)	100	3

Figure S7. Kinase screening results using the KinaseProfiler^{TM} technology provided by Eurofins Inc. The summary of this experiment is shown in Figure 4.

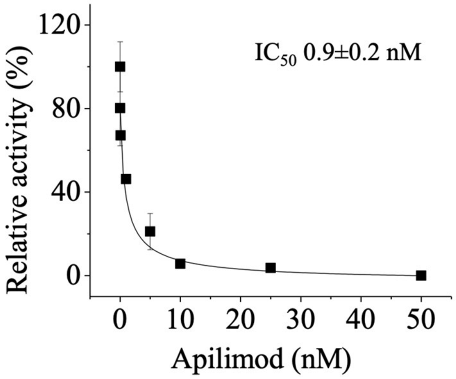

Figure S8. Inhibition of hPIKfyve by Apilimod. Purified hPIKfyve and Apilimod at the indicated concentrations were mixed in the reaction buffer (100 mM Tris-HCl, pH 8.0, 5 mM EGTA, 10 mM $MgCl_2$, and 2 μM diC16-PI4P). The reaction was initiated by adding 50 μM ATP plus 1 μCi [γ-^{32}P] ATP (prepared in the reaction buffer). See more details in Experimental Section.

Part II. General procedures

General procedure for global deacetylation

To a solution of acetyl (Ac)-containing compound (1.0 equiv) in MeOH/DCM (2:1), 3.0 equivalents of NaOMe (25 wt. % in methanol) were added. The reaction mixture was stirred at room temperature for 6 hours, after which amberlite H^+ resin was added to obtain pH~7. Then, the resin was filtered, and the filtrate was evaporated under vacuum. The obtained residue was purified by silica gel chromatography to give the deacylated compound.

General procedure for the one-pot phosphorylation

The solution of starting material (1.0 equiv) and tetrazole (0.45 M in acetonitrile) (1.5-2.0 equiv) in dry acetonitrile was cooled to 0 °C, followed by dropwise addition of phosphoramidite (1.2 equiv) in CH_3CN. The reaction was warmed to room temperature or further heated to reflux. After the consumption of the starting material (indicated by TLC), 3.0 equivalents of *t*-BuOOH (5M in decane) were added. The reaction was stirred for another 6 hours and monitored by TLC. After the completion of reaction, the reaction mixture was poured to aqueous $NaHCO_3$ solution and extracted with DMC 3 times. The organic layer was collected and dried over anhydrous Na_2SO_4. After concentrated under vacuum, the crude product was purified on silica gel column to afford the designed product.

General procedure for TBDPS protection

The starting material (1.0 equiv) was dissolved in anhydrous pyridine and the mixture was cooled to 0 °C. After the addition of 1.2 equivalents of TBDPSCl and 1.0 equivalents of DMAP, the reaction mixture was warmed to room temperature and stirred for overnight. Then, pyridine was evaporated on a high vacuum pump and the residue was dissolved in ethyl acetate. The mixture was washed with 10% aqueous HCl solution to remove the remaining pyridine and the organic layer was dried over anhydrous Na_2SO_4. The volatiles were evaporated, and the residue was purified on a silica column to afford desired compound.

General Procedure for TBDPS removal

To the solution of starting material (1.0 equiv) in THF, 3.0 equivalents of tetrabutylammonium fluoride (1.0 M in THF) was added. The reaction was heated to 60 °C and stirred for 6 hours. After completion, the reaction was diluted with DCM and washed with saturated aqueous $NaHCO_3$ solution and water successively. After dried over anhydrous Na_2SO_4 and concentrated under vacuum, the organic layer was applied to silica gel chromatography for purification and afforded the desired product.

General procedure for Boc removal

TFA was added to the solution of starting material in DCM: MeOH: H_2O (2:1:1). The reaction was stirred at room temperature for overnight. After completion, the solvent was removed under vacuum and the crude product were applied to silica gel chromatography.

General procedure for amidation reaction

The crude product from Boc removal reaction was redissolved in dry DCM. Then, 1.2 equivalent of 1-[bis(dimethylamino)methylene]-1H-1,2,3-triazolo[4,5-b] pyridinium 3-oxid hexafluorophosphate (HATU) and 1.5 equivalent of alkanoic acid was added. After stirred at room temperature for 10 min, 1.5 equivalent of *N*,*N*-diisopropylethylamine (DIEA) was added. The reaction was stirred for another 3 hours and monitored by TLC. The reaction was poured into HCl solution after completion and extracted with DCM 3 times. The combined organic layer was washed with aqueous $NaHCO_3$ solution and brine, respectively. After dried over anhydrous Na_2SO_4, the organic layer was concentrated under vacuum, and further purified by silica gel chromatography to afford desired compound.

General procedure for global debenzylation

To a solution of starting material (50 mg) in DCM/MeOH (1/1), $Pd(OH)_2/C$ (100 mg) was added. The resulting mixture was stirred under H_2 atmosphere and monitored by TLC. After the completion of the reaction, the mixture was filtered and concentrated under vacuum, and the residue was further purified by a LH-20 column to afford quantitative yield.

Safety statement

No unexpected or unusually high safety hazards were encountered during this work.

Part III. Product Preparation and Characterization Data

To prepare phosphoramidite **9**, the commercially available bis(diisopropylamino)chlorophosphine was treated with benzyl alcohol first (**Scheme S1**), which was followed by the coupling with 2-(Boc-amino) ethanol, promoted by tetrazolide diisopropylamino, to afford the phosphoramidite **9**.

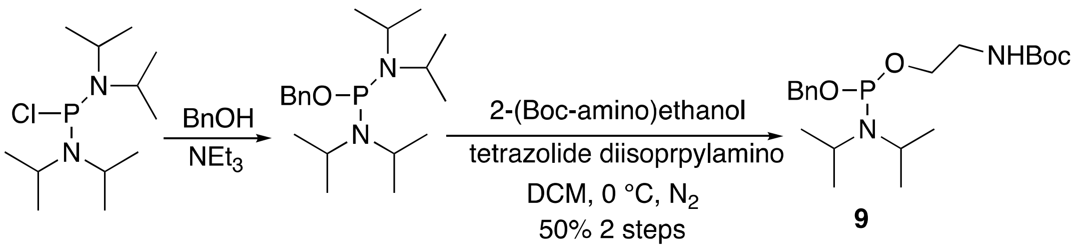

Scheme S1. Synthesis of phosphoramidite **9**

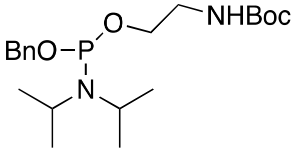

Benzyl-(2-(*tert*-butoxycarbonylamido)ethyl)-N,N-diisopropylphosphoramidite (9)

The mixture of benzyl alcohol (168 uL, 1.6 mmol) and triethylamine (700 uL ,4.8 mmol) in Et_2O was added dropwise to the solution of bis(dimethylamino)chlorophosphine (250 mg, 1.6 mmol) in Et_2O under N_2 at 0 °C. After stirred for 2 hours, the reaction mixture was filtered, and the filtration was concentrated under vacuum to obtain bis(dimethylamino)benzyloxyl phosphine. To a solution of 2-(Boc-amino)ethanol (541.56 mg, 1.6 mmol) and diisopropylammonium tetrazolide (172.55 mg, 1.92 mmol) in DCM/CH_3CN (2:1), 1.0 equivalents of bis(dimethylamino)benzyloxyl phosphine (257.77 mg, 1.6 mmol) was added. The reaction was stirred at room temperature under N_2 for 4 hours, after which the reaction mixture was poured into saturated aqueous $NaHCO_3$ solution, and the aqueous layer was extracted by DCM 3 times. The organic layer was combined and dried over Na_2SO_4. DCM was removed under vacuum and the resulting residue was purified by silica gel chromatography (prewashed with 3% triethylamine in hexanes to give the phosphoramidite **9** in 50% yield.

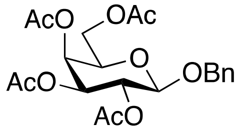

Benzyl 2,3,4,6-tetra-O-acetyl-β-D-galactopyranoside (11)

Benzyl alcohol (3.0 ml, 30.8 mmol) and boron trifluoride etherate (4.0 ml, 31.0 mmol) were mixed in dry DCM and cooled to 0 °C. After stirred at 0 °C for 1 hour, β-D-galactose pentaacetate (10.0 g, 25.60 mmol) was added slowly to the reaction mixture. The reaction was maintained at 0 °C and stirred for another 4 hours as monitored by TLC. After completion, the reaction was quenched by adding triethylamine. Then, the reaction mixture was diluted with DCM, and washed with saturated aqueous $NaHCO_3$ solution and water successively. The organic layer was collected and dried over anhydrous Na_2SO_4. The volatiles were evaporated, and the resulting residue was purified by column chromatography to afford compound **11**. $[\alpha]^{25}_{D}$ 27.96 (c = 1, CH_2Cl_2); ^1H NMR (500 MHz, $CDCl_3$) δ 7.39 – 7.24 (m, 5H), 5.38 (dd, J = 3.5, 1.2 Hz, 1H, H-4), 5.28 (dd, J = 10.4, 7.9 Hz, 1H, H-2), 4.98 (dd, J = 10.4, 3.5 Hz, 1H, H-3), 4.91 (d, J = 12.3 Hz, 1H, Bn), 4.63 (d, J = 12.3 Hz, 1H, Bn), 4.51 (d, J = 7.9 Hz, 1H, H-1), 4.21 (dd, J = 11.3, 6.5 Hz, 1H, H-6), 4.18 (dd, J = 11.3, 6.5 Hz, 1H, H-6), 3.88 (td, J = 6.8, 1.2 Hz, 1H, H-5), 2.16 (s, 3H), 2.07 (s, 3H), 2.01 (s, 3H),

1.97 (s, 3H). ^{13}C NMR (126 MHz, $CDCl_3$) δ 170.57, 170.44, 170.31, 169.58, 136.83, 128.71, 128.60, 128.15, 127.89, 127.12, 99.91, 89.84, 71.05, 70.87, 70.82, 68.97, 67.18, 61.45, 20.89, 20.85, 20.83, 20.73. HRMS: $[M+H]^+$ $C_{21}H_{26}O_{10}$ calcd 439.1526, obsd 439.1552.

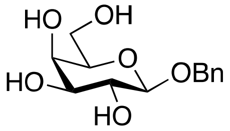

Benzyl 2,3,4,6-*tetra*-hydroxyl-*β*-D-galactopyranoside (12)

Compound **12** was prepared from compound **11** (4.5 g, 10.27mmol) by following the general procedure for global deacetylation, providing the product in 93% yield. $[\alpha]^{25}_{D}$ 153.75 (c = 0.1, CH_2Cl_2); ^{1}H NMR (500 MHz, CD_3OD) δ 7.44 – 7.39 (m, 2H), 7.34 – 7.30 (m, 2H), 7.29 – 7.22 (m, 1H), 4.93 (d, *J* = 11.9 Hz, 1H, Bn), 4.66 (d, *J* = 11.9 Hz, 1H, Bn), 4.31 (d, *J* = 7.7 Hz, 1H, H-1), 3.84 (d, *J* = 3.42 Hz, 1H, H-4), 3.79 (dd, *J* = 11.4, 6.8 Hz, 1H, H-6), 3.74 (dd, *J* = 11.4, 6.8 Hz, 1H, H-6), 3.58 (dd, *J* = 9.7, 7.7 Hz, 1H, H-2), 3.50 (ddd, *J* = 6.6, 5.3, 1.2 Hz, 1H, H-5), 3.46 (dd, *J* = 9.7, 3.42 Hz, 1H, H-3). ^{13}C NMR (126 MHz, CD_3OD) δ 137.75, 127.92, 127.84, 127.79, 127.28, 127.24, 102.48, 75.31, 73.55, 71.15, 70.26, 68.95, 61.14. HRMS: $[M+Na]^+$ $C_{13}H_{18}NaO_6$ calcd 293.1001, obsd 293.1009.

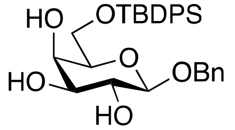

Benzyl 6-*O*-(*tert*-butyldiphenylsilyl)-2,3,4-*tri*-hydroxyl-*β*-D-galactopyranoside (13)

Compound **13** was prepared from compound **12** (2.58 g, 9.55 mmol) by following the general procedure for TBDPS protection, providing the product in 98% yield. $[\alpha]^{25}_{D}$ -30.53 (c = 2, CH_2Cl_2); ^{1}H NMR (500 MHz, $CDCl_3$) δ 7.75 – 7.67 (m, 6H), 7.48 – 7.29 (m, 9H), 4.92 (d, *J* = 11.6 Hz, 1H, Bn), 4.59 (d, *J* = 11.6 Hz, 1H, Bn), 4.32 (d, *J* = 7.7 Hz, 1H, H-1), 4.09 (dd, *J* = 3.5, 1.1 Hz, 1H, H-4), 4.02 – 3.92 (m, 2H, H-6), 3.72 (dd, *J* = 9.5, 7.7 Hz, 1H, H-2), 3.58 (dd, *J* = 9.5, 3.5 Hz, 1H, H-5), 3.56 – 3.53 (1H, H-3), 1.08 (s, 9H). ^{13}C m, NMR (126 MHz, $CDCl_3$) δ 149.67, 136.97, 136.15, 135.66, 135.56, 134.81, 133.07, 132.86, 129.92, 129.91, 129.64, 128.51, 128.23, 128.03, 127.84, 127.82, 127.72, 123.85, 101.67, 77.23, 74.45, 73.62, 72.25, 70.72, 68.87, 63.11, 26.80, 26.56, 19.20. HRMS: $[M+Na]^+$ $C_{29}H_{36}NaO_6Si$ calcd 531.2179, obsd 531.2189.

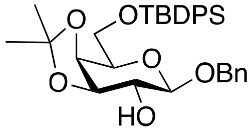

Benzyl 6-*O*-(*tert*-butyldiphenylsilyl)-3,4-*O*-isopropylidene-*β*-D-galactopyranoside (7)

Compound **13** (4.75 g, 9.36 mmol) was dissolved in acetone, then 2,2-dimethoxy propane (1.72 mL, 14.05 mmol) and camphorsulfonic acid (0.42 g, 1.87 mmol) were added. After stirred at room temperature overnight, Et_3N was added to neutralize the excess CSA and bring the pH around 7. The reaction mixture was concentrated under vacuum, and the residue was diluted with DCM and washed with aqueous $NaHCO_3$ solution and saturated NaCl solution successively. The organic layer was collected and dried over anhydrous Na_2SO_4. The volatiles were removed under vacuum and the resulted residue was purified by silica gel column chromatography to afford the designed product **7** in 96% yield. $[\alpha]^{25}_{D}$ 0.72 (c = 0.7, CH_2Cl_2); ^{1}H NMR (500 MHz, $CDCl_3$) δ 7.75 – 7.68 (m, 6H), 7.47 – 7.28 (m, 9H), 4.90 (d, *J* = 11.6 Hz, 1H, Bn), 4.58 (d, *J* = 11.6 Hz, 1H, Bn), 4.26 – 4.24 (m, 1H, H-4), 4.23 (d, *J* = 8.4 Hz, 1H, H-1), 4.05 (dd, *J* = 7.4, 5.4 Hz, 1H, H-3), 4.02 – 3.95 (m, 2H, H-6), 3.88 – 3.84 (m, 1H, H-5), 3.64 – 3.59 (m, 1H, H-2), 1.51 (s, 3H), 1.34 (s, 3H),

1.07 (s, 9H). ^{13}C NMR (126 MHz, $CDCl_3$) δ 137.00, 135.65, 135.63, 133.49, 133.41, 129.68, 128.52, 128.09, 128.03, 127.67, 127.62, 109.45, 96.46, 76.18, 72.85, 69.70, 69.38, 68.95, 63.02, 27.76, 26.78, 25.91, 19.22, 14.20, 1.02. HRMS: $[M+Na]^+$ $C_{32}H_{40}NaO_6Si$ calcd 571.2492, obsd 571.2498.

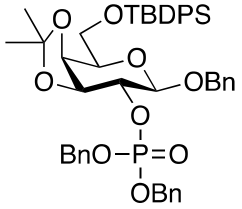

Benzyl 2-*O*-(dibenzylphosphono)-6-*O*-(*tert*-butyldiphenylsilyl)-3,4-*O*-isopropylidene-*β*-D-galactopyranoside (14)

Compound **14** was prepared from compound **7** (414.5 mg, 0.755 mmol) and phosphoramidite **8** by following the general procedure for one-pot phosphorylation, providing the product in 65% yield. $[\alpha]^{25}_D$ -5.09 (c = 0.6, CH_2Cl_2); ^1H NMR (500 MHz, $CDCl_3$) δ 7.74 – 7.66 (m, 5H), 7.46 – 7.21 (m, 20H), 5.06 – 4.99 (m, 3H, Bn), 4.96 (dd, *J* = 11.9, 7.5 Hz, 1H, Bn), 4.88 (d, *J* = 11.7 Hz, 1H, Bn), 4.58 (d, *J* = 11.7 Hz, 1H, Bn), 4.46 – 4.38 (m, 2H, H-1, H-2), 4.26 (dd, *J* = 5.3, 2.0 Hz, 1H, H-4), 4.21 (t, *J* = 6.0 Hz, 1H, H-3), 3.97 (dd, *J* = 6.7, 1.5 Hz, 2H, H-6), 3.87 (dt, *J* = 8.0, 4.0 Hz, 1H, H-5), 1.49 (s, 3H), 1.32 (s, 3H), 1.07 (s, 9H). ^{13}C NMR (126 MHz, $CDCl_3$) δ 136.74, 135.62, 135.58, 133.33, 133.25, 129.76, 128.57, 128.41, 128.36, 128.34, 128.28, 128.17, 128.14, 127.81, 127.75, 127.69, 127.68, 127.43, 110.49, 99.02, 98.97, 80.83, 79.15, 79.09, 78.07, 78.05, 73.68, 73.52, 70.24, 69.09, 69.05, 68.99, 68.95, 62.64, 27.86, 26.98, 26.75, 26.39, 25.76, 19.22, 14.21. HRMS: $[M+Na]^+$ $C_{46}H_{53}NaO_9PSi$ calcd 831.3094, obsd 831.3122.

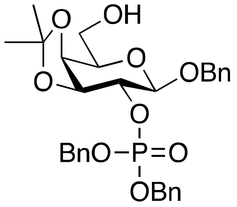

Benzyl 2-*O*-(dibenzylphosphono)-3,4-*O*-isopropylidene-*β*-D-galactopyranoside (15)

Compound **15** was prepared from compound **14** (505 mg, 0.62 mmol) by following the general procedure for TBDPS removal reaction, providing the product in 76% yield. $[\alpha]^{25}_D$ -6.4 (c = 1, CH_2Cl_2); ^1H NMR (500 MHz, $CDCl_3$) δ 7.38 – 7.22 (m, 15H), 5.07 – 4.95 (m, 4H, Bn), 4.88 (d, *J* = 11.8 Hz, 1H, Bn), 4.66 (d, *J* = 11.8 Hz, 1H, Bn), 4.50 (d, *J* = 8.1 Hz, 1H, H-1), 4.43 (ddd, *J* = 10.2, 8.1, 7.1 Hz, 1H, H-2), 4.25 (dd, *J* = 7.1, 5.5 Hz, 1H, H-3), 4.17 – 4.16 (m, 1H, H-4), 4.01 – 3.95 (m, 1H, H-5), 3.83 (qt, *J* = 7.1, 2.8 Hz, 2H, H-6), 1.51 (s, 3H), 1.33 (s, 3H). ^{13}C NMR (126 MHz, $CDCl_3$) δ 171.19, 136.84, 136.09, 128.40, 128.38, 128.24, 128.21, 128.20, 127.94, 127.82, 127.71, 110.91, 99.53, 99.49, 78.80, 78.75, 78.19, 78.16, 74.22, 73.32, 71.03, 69.15, 69.11, 69.06, 69.02, 62.34, 60.42, 27.77, 26.42, 21.08, 14.21. HRMS: $[M+Na]^+$ $C_{30}H_{35}NaO_9P$ calcd 593.1917 obsd 593.1935.

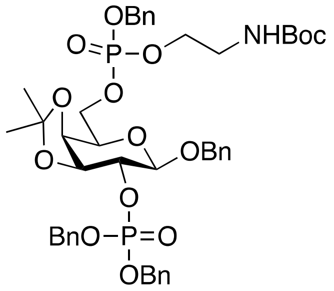

Benzyl 2-*O*-(dibenzylphosphono)-6-*O*-((benzyl)-(2-(*tert*-butoxycarbonylamido)-ethyl)-phospono)-3,4-*O*-isopropylidene-*β*-D-galactopyranoside (16)

Compound **16** was prepared from compound **15** (243mg, 0.43 mmol) and the freshly prepared phosphoramidite **9** (206mg, 0.52 mmol) by following the general procedure for the one-pot phosphorylation reaction, which providing the product in 72% yield. $[\alpha]^{25}_{D}$ -2.86 (c = 0.7, CH_2Cl_2); ^1H NMR (500 MHz, $CDCl_3$) δ 7.43 – 7.20 (m, 20H, Bn), 5.12 (dd, *J* = 8.9, 2.4 Hz, 2H, Bn), 5.06 – 4.92 (m, 4H, Bn), 4.87 (dd, *J* = 11.8, 3.0 Hz, 1H, Bn), 4.56 (dd, *J* = 11.8, 6.6 Hz, 1H, Bn), 4.43 (d, *J* = 6.9 Hz, 1H, H-1), 4.41 – 4.36 (m, 1H, H-2), 4.31 – 4.23 (m, 2H, H-6), 4.20 (q, *J* = 5.8 Hz, 1H, H-3), 4.13 – 4.09 (m, 1H, H-4), 4.08 – 4.03 (m, 2H, OCH_2), 3.93 (ddd, *J* = 7.2, 5.1, 2.1 Hz, 1H, H-5), 3.36 (q, *J* = 4.9 Hz, 2H, CH_2N),1.46 (s, 3H), 1.42 (s, 9H), 1.29 (s, 3H). ^{13}C NMR (126 MHz, $CDCl_3$) δ 136.43, 135.83, 135.76, 135.49, 128.88, 128.74, 128.44, 128.42, 128.35, 128.33, 128.15, 127.98, 127.82, 127.70, 110.99, 110.97, 98.97, 98.92, 78.74, 78.69, 77.84, 73.30, 71.56, 71.50, 70.63, 69.78, 69.32, 69.23, 67.27, 66.26, 40.85, 28.36, 27.68, 26.35, 14.20. HRMS: $[M+Na]^+$ $C_{44}H_{55}NNaO_{14}P_2$ calcd 906.2996, obsd 906.3080.

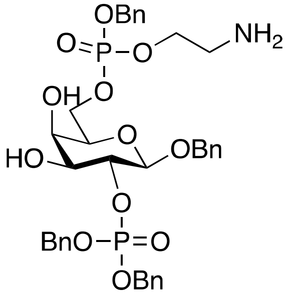

Benzyl 2-*O*-(dibenzylphosphono)-6-*O*-((benzyl)-(2-aminoethyl)-phospono)-β-D-galactopyranoside (17)

Compound **17** was prepared from compound **16** (216 mg, 0.25 mmol) by following the general procedure for Boc deprotection. $[\alpha]^{25}_{D}$ -25.0 (c = 0.1, CH_2Cl_2); ^1H NMR (500 MHz, CD_3OD) δ 7.52 – 7.05 (m, 20H, 4Bn), 5.19 (t, *J* = 8.2 Hz, 2H, CH_2Ph), 5.08 (d, *J* = 7.4 Hz, 2H, CH_2Ph), 4.94 (m, 2H, CH_2Ph), 4.91 – 4.83 (m, 1H, CH_2Ph), 4.62 (dd, *J* = 7.9, 1.8 Hz, 1H, H-1), 4.59 – 4.50 (m, 1H, CH_2Ph), 4.46 (tdd, *J* = 10.1, 8.0, 2.6 Hz, 1H, H-2), 4.40 – 4.17 (m, 4H, H-6, $POCH_2$, H-4), 3.88 (dt, *J* = 8.6, 3.9 Hz, 1H, H-6), 3.86 – 3.72 (m, 2H, H-3, H-5), 3.25 – 3.17 (m, 2H, CH_2N). ^{13}C NMR (126 MHz, CD_3OD) δ 137.04, 135.56, 128.63, 128.61, 128.44, 128.16, 128.14, 128.11, 128.10, 128.07, 128.03, 128.01, 127.76, 127.72, 127.59, 127.58, 127.47, 127.40, 100.24, 78.54, 78.49, 73.24, 73.18, 71.97, 70.59, 69.98, 69.93, 69.38, 69.34, 69.16, 69.11, 67.16, 64.01, 39.44, 39.38. HRMS: $[M+H]^+$ $C_{36}H_{44}NO_{12}P_2$ calcd 744.2339, obsd 744.2365.

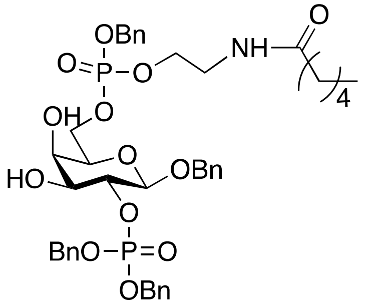

Benzyl 2-*O*-(dibenzylphosphono)-6-*O*-((benzyl)-(2-hexanamidoethyl)-phospono)- *β* -D-galactopyranoside (17-1)

Compound **17-1** was prepared from the crud product **17** and hexanoic acid (19.74mg, 0.17mmol) by following the general procedure for amidation reaction, affording the compound **in** 85% yield in two steps. $[\alpha]^{25}_{D}$ -6.80 (c = 0.25, CH_2Cl_2); ^{1}H NMR (500 MHz, $CDCl_3$) δ 7.42 – 7.14 (m, 20H, 4Bn), 5.11 (dd, *J* = 9.0, 4.0 Hz, 2H, CH_2Ph), 5.04 (ddd, *J* = 23.3, 8.1, 1.4 Hz, 2H, CH_2Ph), 4.97 (d, *J* = 81.9 Hz, 2H, CH_2Ph), 4.91 (d, *J* = 15.9 Hz, 1H, CHPh), 4.58 (dd, *J* = 11.7, 8.0 Hz, 1H, CHPh), 4.46 (dd, *J* = 7.7, 3.9 Hz, 1H, H-1), 4.40 – 4.32 (m, 1H, H-2), 4.32 – 4.23 (m, 2H, H-6), 4.10 (dq, *J* = 11.4, 5.7 Hz, 2H, $POCH_2$), 3.92 (dd, *J* = 18.0, 3.5 Hz, 1H, H-4), 3.71 (ddd, *J* = 8.9, 5.2, 3.5 Hz, 1H, H-3), 3.66 (q, *J* = 6.1 Hz, 1H, H-5), 3.57 – 3.42 (m, 2H, CH_2N), 2.18 – 2.09 (m, 2H, $COCH_2$), 1.60 (qd, *J* = 8.0, 3.7 Hz, 2H, $COCH_2CH_2$), 1.36 – 1.21 (m, 4H), 0.88 (td, *J* = 7.0, 2.4 Hz, 3H). ^{13}C NMR (126 MHz, $CDCl_3$) δ174.80, 128.91, 128.76, 128.69, 128.59, 128.49, 128.42, 128.19, 128.16, 128.07, 127.97, 127.78, 99.69, 78.40, 78.36, 77.29, 77.03, 76.78, 72.71, 71.05, 69.98, 69.87, 69.82, 67.87, 67.20, 39.72, 36.53, 31.43, 29.71, 25.28, 22.40, 13.96. HRMS: $[M+H]^+$ $C_{42}H_{53}NO_{13}P_2$ calcd 842.3070, obsd 842.3062.

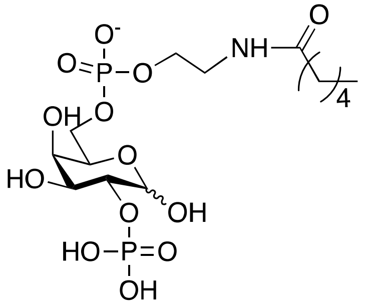

2-*O*-Phosphono-6-*O*-((2-hexanamidoethyl)-phospono)-*α*/*β*-D-galactopyranoside (1)

Compound **1** was prepared from compound **17-1** (68.65 mg, 0.08 mmol) by following the general procedure for global debenzylation, leading to the formation of product in a quantitative yield. $[\alpha]^{25}_{D}$ -3.58 (c = 0.32, CH_2Cl_2); ^{1}H NMR (500 MHz, CD_3OD : $CDCl_3$: D_2O = 4:2:1) δ 5.35 (dd, *J* = 7.0, 2.6 Hz, 1H, H-1a), 4.35 (td, *J* = 9.0, 3.6 Hz, 1H, H-2a), 4.25 (dd, *J* = 7.3, 4.9 Hz, 1H, H-6a), 4.21 – 4.05 (m, 4H, H-6a, H-4a, $POCH_2$), 3.97 (d, *J* = 2.36 Hz, 1H, H-3a), 3.86 – 3.79 (m, 1H, H-5a), 3.53 – 3.46 (m, 2H, CH_2N), 2.30 (td, *J* = 7.7, 3.8 Hz, 2H, $COCH_2$), 1.64 (p, *J* = 7.5 Hz, 2H, $COCH_2CH_2$), 1.42 – 1.27 (m, 4H), 0.93 (t, *J* = 6.9 Hz, 3H). ^{13}C NMR (126 MHz, CD_3OD : $CDCl_3$: D_2O = 4:2:1) δ 176.78, 118.43, 92.27, 75.70, 70.47, 69.71, 66.29, 55.11, 49.51, 49.34, 49.17, 49.00, 48.83, 48.66, 48.49, 36.42, 32.15, 26.44, 23.16, 18.33, 14.21. HRMS: $[M-H]^-$ $C_{14}H_{28}NO_{13}P_2$ calcd 480.1036, obsd 480.1038.

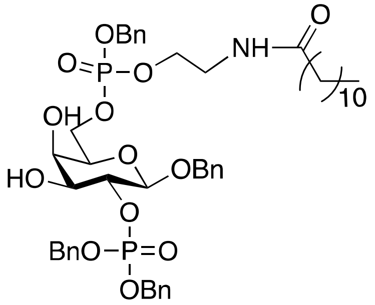

Benzyl 2-*O*-(dibenzylphosphono)-6-*O*-((benzyl)-(2-dodecanamidoethyl)-phospono)-3,4-*di*-hydroxyl-β-D-galactopyranoside (17-2)

Compound **17-2** was synthesized from compound **17** and lauric acid by following the general procedure for amidation reaction, affording the compound in 70% yield in two steps. $[\alpha]^{25}_{D}$ -6.0 (c = 0.3, CH_2Cl_2); ^1H NMR (500 MHz, $CDCl_3$) δ 7.51 – 7.09 (m, 20H), 5.11 (ddd, *J* = 9.2, 3.8, 1.7 Hz, 2H, CH_2Ph), 5.05 (d, *J* = 7.9 Hz, 2H, CH_2Ph), 5.02 – 4.93 (m, 2H, CH_2Ph), 4.90 (dt, *J* = 11.6, 2.4 Hz, 1H, C-1CH_2Ph), 4.58 (ddd, *J* = 11.8, 7.7, 1.7 Hz, 1H, C-1CH_2Ph), 4.46 (ddd, *J* = 8.0, 3.7, 1.8 Hz, 1H, H-1), 4.38 (tdt, *J* = 8.9, 4.8, 2.1 Hz, 1H, H-2), 4.27 (tt, *J* = 7.8, 3.5 Hz, 2H, H-6), 4.10 (dqd, *J* = 12.4, 6.1, 3.3 Hz, 2H, H-7), 3.93 (dt, *J* = 17.4, 2.4 Hz, 1H, H-4), 3.71 (dt, *J* = 8.9, 3.5 Hz, 1H, H-3), 3.65 (q, *J* = 6.3 Hz, 1H, H-5), 3.49 (q, *J* = 4.8 Hz, 2H, H-8), 2.13 (t, *J* = 7.7 Hz, 2H, $COCH_2$), 1.60 (dt, *J* = 15.4, 7.8 Hz, 2H, $COCH_2CH_2$), 1.33 – 1.23 (m, 16H), 0.88 (td, *J* = 6.9, 2.0 Hz, 3H). ^{13}C NMR (126 MHz, $CDCl_3$) δ 173.79, 136.74, 129.01, 128.86, 128.76, 128.69, 128.59, 128.57, 128.53, 128.30, 128.27, 128.18, 128.09, 128.07, 127.88, 99.75, 78.50, 78.46, 77.42, 77.16, 76.91, 72.84, 71.12, 71.03, 70.02, 70.02, 68.11, 67.30, 66.01, 39.80, 36.69, 32.03, 29.76, 29.74, 29.64, 29.61, 29.52, 29.47, 29.45, 25.75, 22.81, 14.26. HRMS: $[M+H]^+$ $C_{48}H_{65}NO_{13}P_2$ calcd 926.4009, obsd 926.3961.

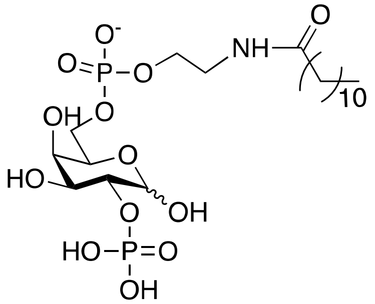

2-*O*-Phosphono-6-*O*-((2-dodecanamidoethyl)-phospono)-α/β-D-galactopyranoside (2)

Compound **2** was synthesized from **17-2** in quantitative yield following the general procedure of global debenzylation reaction. $[\alpha]^{25}_{D}$ -0.8 (c = 0.88, CH_2Cl_2); ^1H NMR (500 MHz, CD_3OD : $CDCl_3$: D_2O = 4:2:1) δ 5.36 (d, *J* = 3.5 Hz, 2H, H-1a), 4.64 (d, *J* = 7.6 Hz, 1H, H-1b), 4.39 – 4.33 (m, 2H, H-2a), 4.29 – 4.22 (m, 1H, H-5), 4.20 – 4.10 (m, 2H, H-6), 4.06 (dt, *J* = 7.1, 5.4 Hz, 3H, $POCH_2$), 3.96 (t, *J* = 3.6 Hz, 1H, H-3), 3.88 (d, *J* = 3.2 Hz, 1H, H-4), 3.47 (q, *J* = 5.3 Hz, 3H, CH_2NCO), 2.01 (p, *J* = 2.5 Hz, 3H, $COCH_2$), 1.61 (dd, *J* = 9.7, 4.9 Hz, 3H,$COCH_2CH_2$), 1.31 (d, *J* = 6.8 Hz, 24H), 0.91 (t, *J* = 6.9 Hz, 5H). ^{13}C NMR (126 MHz, CD_3OD : $CDCl_3$: D_2O = 4:2:1) δ 176.70, 92.40, 75.67, 70.57, 69.79, 69.17, 66.52, 40.71, 36.86, 34.70, 32.90, 30.59, 30.57, 30.46, 30.31, 30.29, 30.14, 26.79, 23.59, 14.40. HRMS: $[M-H]^-$ $C_{20}H_{40}NO_{13}P_2$ calcd 564.1975, obsd 564,1978.

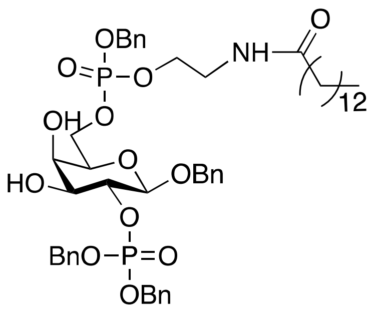

Benzyl 2-*O*-(dibenzylphosphono)-6-*O*-((benzyl)-(2-tetradecanamidoethyl)-phospono) -*β*-D-galactopyranoside (17-3)

Compound **17-3** was synthesized from compound **17** and myristic acid by following the general procedure for amidation reaction, affording the compound in 63% yield in two steps. $[\alpha]^{25}_{D}$ -6.67 (c = 0.03, CH_2Cl_2); ^1H NMR (500 MHz, $CDCl_3$) δ 7.45 – 7.11 (m, 20H), 6.15 (dd, *J* = 14.2, 8.5 Hz, 1H, NH), 5.12 (dd, *J* = 9.0, 4.1 Hz, 2H, CH_2Ph), 5.07 – 5.02 (m, 2H, CH_2Ph), 5.01 – 4.94 (m, 2H, CH_2Ph), 4.91 (dd, *J* = 11.9, 3.3 Hz, 1H, C-1CH_2Ph), 4.58 (dd, *J* = 11.7, 8.1 Hz, 1H, C-1CH_2Ph), 4.46 (dd, *J* = 7.7, 3.7 Hz, 1H, H-1), 4.38 – 4.31 (m, 1H, H-2), 4.27 (dddd, *J* = 13.9, 10.8, 6.1, 3.4 Hz, 2H, H-6), 4.11 (dq, *J* = 9.0, 4.6 Hz, 2H, $POCH_2$), 3.96 – 3.88 (m, 1H, H-4), 3.72 (ddd, *J* = 9.2, 5.9, 3.5 Hz, 1H, H-3), 3.70 – 3.63 (m, 1H, H-5), 3.50 (h, *J* = 4.7 Hz, 2H, CH_2N), 2.16 – 2.10 (m, 2H, $COCH_2$), 1.61 (tq, *J* = 14.3, 7.2 Hz, 2H), 1.25 (d, *J* = 6.53 Hz, 20H), 0.89 (td, *J* = 7.0, 1.7 Hz, 3H). ^{13}C NMR (126 MHz, $CDCl_3$) δ172.98, 129.86, 129.78, 129.51, 129.48, 129.44, 129.37, 129.30, 129.29, 129.26, 128.98, 128.81, 101.54, 80.03, 73.53, 73.50, 71.88, 70.48, 61.53, 49.51, 49.34, 49.29, 49.17, 49.00, 48.83, 48.66, 48.49, 38.88, 37.06, 34.95, 33.08, 30.81, 30.79, 30.77, 30.65, 30.62, 30.49, 30.44, 30.33, 30.25, 26.97, 26.10, 23.75, 20.86, 14.47. HRMS: $[M+Na]^+$ $C_{50}H_{69}NNaO_{13}P_2$ calcd 976.4136, obsd 976.4095.

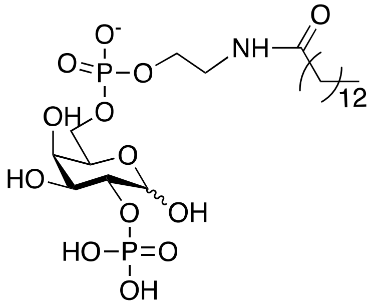

2-*O*-Phosphono-6-*O*-((2-tetradecanamidoethyl)-phospono)-*α*/*β*-D-galactopyranoside (3)

Compound **3** was synthesized from **17-3** in quantitative yield following the general procedure of global debenzylation reaction. $[\alpha]^{25}_{D}$ -5.2 (c = 0.15, CH_2Cl_2); ^1H NMR (500 MHz, CD_3OD : $CDCl_3$: D_2O = 4:2:1) δ 5.43-5.29 (m, 1H, H-1a), 4.31 (m, 1H, H-2), 4.19 (d, *J* = 6.7 Hz, 1H, H-5), 4.01-3.91 (m, 5H, H-6, H-4, $POCH_2$), 3.66 (d, *J* = 3.7 Hz, 1H, H-3), 3.40 (t, *J* = 5.1 Hz, 2H, CH_2N), 2.23-2.15 (m, 2H, $COCH_2$), 1.58 (q, *J* = 7.2 Hz, 2H $COCH_2CH_2$), 1.26 (t, *J* = 7.39 Hz, 20H), 0.85 (t, *J* = 6.69 Hz, 3H). ^{13}C NMR (126 MHz, CD_3OD : $CDCl_3$: D_2O = 4:2:1) δ 176.85, 92.55, 75.82, 70.72, 69.94, 69.32, 66.67, 40.86, 37.01, 34.85, 33.05, 30.74, 30.72, 30.61, 30.46, 30.44, 30.29, 30.14, 26.94, 26.03, 23.74, 14.55. HRMS: $[M-H]^-$ $C_{22}H_{44}NO_{13}P_2$ calcd 592.2288, obsd 592.2292.

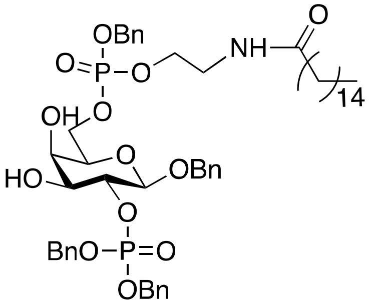

Benzyl 2-*O*-(dibenzylphosphono)-6-*O*-((benzyl)-(2-palmitamidoethyl)-phospono)-β-D-galactopyranoside (17-4)

Compound **17-4** was synthesized from compound **17** and palmitic acid by following the general procedure for amidation reaction, affording the compound in 69% yield in two steps. $[\alpha]^{25}_{D}$ -6.67 (c = 0.15, CH_2Cl_2); ^{1}H NMR (500 MHz, $CDCl_3$) δ 7.49 – 7.06 (m, 20H), 5.10 (dd, *J* = 9.0, 3.9 Hz, 2H), 5.03 (dd, *J* = 8.0, 1.5 Hz, 2H), 4.99 – 4.92 (m, 2H), 4.89 (dd, *J* = 12.0, 3.5 Hz, 1H, CHPh), 4.57 (dd, *J* = 11.7, 7.7 Hz, 1H, CHPh), 4.45 (dd, *J* = 7.7, 3.9 Hz, 1H, H-1), 4.37 (dtdt, *J* = 13.3, 7.8, 5.7, 2.3 Hz, 1H, H-2), 4.32 – 4.20 (m, 2H, H-6), 4.09 (dq, *J* = 10.0, 5.1 Hz, 2H, $POCH_2$), 3.92 (dd, *J* = 17.4, 3.5 Hz, 1H, H-4), 3.70 (dt, *J* = 8.8, 4.3 Hz, 1H, H-3), 3.64 (q, *J* = 6.1 Hz, 1H, H-5), 3.47 (hept, *J* = 4.6 Hz, 2H, CH_2N), 2.12 (t, *J* = 7.7 Hz, 2H, $COCH_2$), 1.57 (p, *J* = 6.9 Hz, 2H), 1.32 – 1.18 (m, 24H), 0.88 (t, *J* = 6.8 Hz, 3H). ^{13}C NMR (126 MHz, $CDCl_3$) δ 173.76, 136.76, 135.59, 129.01, 128.86, 128.77, 128.69, 128.59, 128.57, 128.53, 128.52, 128.30, 128.27, 128.18, 128.09, 128.07, 127.88, 99.75, 78.52, 78.48, 72.85, 71.12, 71.03, 70.05, 69.97, 69.92, 68.08, 67.30, 39.80, 36.69, 32.05, 29.83, 29.80, 29.78, 29.65, 29.52, 29.49, 29.46, 25.75, 22.82, 14.26. HRMS: $[M+H]^+$ $C_{52}H_{73}NO_{13}P_2$ calcd 982.4635, obsd 982.4592.

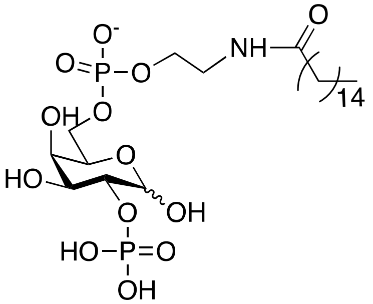

2-*O*-Phosphono-6-*O*-((2-palmitamidoethyl)-phospono)-α/β-D-galactopyranoside (4)

Compound **4** was synthesized from **17-4** in quantitative yield following the general procedure of global debenzylation reaction. $[\alpha]^{25}_{D}$ -3.2 (c = 0.8, CH_2Cl_2); ^{1}H NMR (500 MHz, CD_3OD : $CDCl_3$: D_2O = 4:2:1) δ 5.33 (dd, *J* = 6.7, 2.5 Hz, 1H, H-1a), 4.63 (d, *J* = 7.5 Hz, 0.5H, H-1b), 4.39 – 4.33 (m, 1H, H-2a), 4.24 (t, *J* = 6.2 Hz, 1H, H-5a), 4.19 – 4.12 (m, 2H, H-6a),), 4.12 – 4.09 (m, 1H, H-6b), 4.06 (q, *J* = 6.1 Hz, 4H, POCH2), 4.00 – 3.94 (m, 1H, H-3a), 3.88 (d, *J* = 3.2 Hz, 1H, H-4a), 3.46 (t, *J* = 5.4 Hz, 2H, CH_2N), 2.24 (td, *J* = 7.6, 2.6 Hz, 2H, $COCH_2$), 1.60 (p, *J* = 7.0 Hz, 2H), 1.39 – 1.23 (m, 24H), 0.91 (t, *J* = 6.7 Hz, 3H). ^{13}C NMR (126 MHz, CD_3OD : $CDCl_3$: D_2O = 4:2:1) δ 177.25, 92.47, 75.76, 74.63, 70.63, 69.85, 66.65, 49.66, 49.49, 49.32, 49.15, 48.98, 48.81, 48.64, 40.78, 36.92, 32.99, 30.73, 30.71, 30.69, 30.56, 30.40, 30.39, 30.23, 26.89, 23.69, 14.54. HRMS: $[M-H]^-$ $C_{24}H_{48}NO_{13}P_2$ calcd 620.2601, obsd 620.2609.

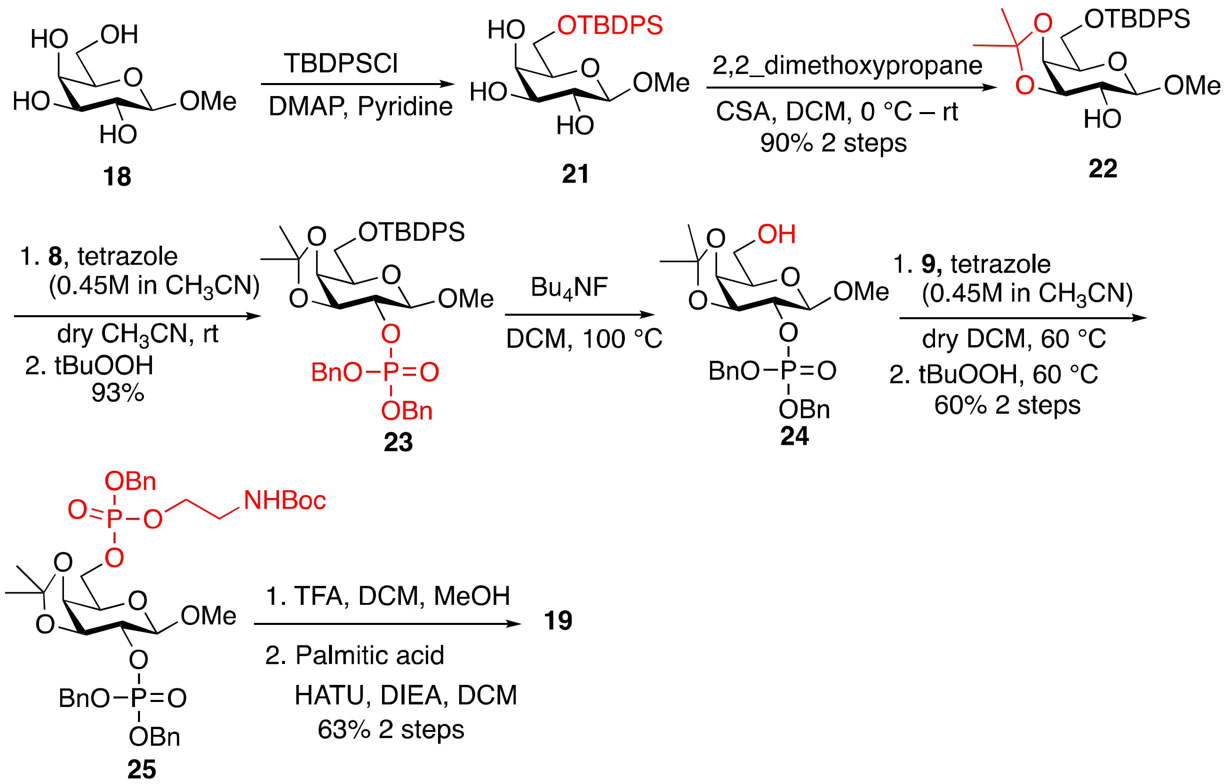

Scheme S2. Synthesis of methyl-galactoside **19**

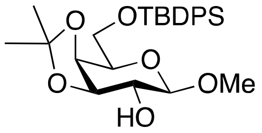

Methyl 6-*O*-(*tert*-butyldiphenylsilyl)-2-hydroxyl-3,4-*O*-isopropylidene-β-D-galactopyranoside (22)

The 6-OH of methyl-β-D-galactopyranoside (2 g, 10.3 mmol) was protected by TBDPSCl with the method of the general procedure for TBDPS protection. Then, the crude compound was dissolved in acetone, and 2,2-dimethoxy propane (1.8 mL, 12.36 mmol) and camphorsulfonic acid (0.12 g, 0.52 mmol) was added. After stirred at room temperature overnight, Et_3N was added to neutralize the excess CSA and bring the pH around 7. The reaction mixture was concentrated under vacuum, and the residue was diluted with DCM and washed with aqueous $NaHCO_3$ solution and saturated NaCl solution successively. The organic layer was collected and dried over anhydrous Na_2SO_4. The volatiles were removed under vacuum and the resulted residue was purified by silica gel column chromatography to afford the designed product **22** in 90% yield in two steps. $[\alpha]^{25}_{D}$ -2.9 (c = 3, CH_2Cl_2); ^{1}H NMR (500 MHz, $CDCl_3$) δ 7.74 – 7.69 (m, 4H), 7.47 – 7.35 (m, 6H), 4.29 (dd, J = 5.4, 2.2 Hz, 1H, H-4), 4.10 – 4.07 (m, 2H, H-1, H-3), 4.02 – 3.92 (m, 2H, H-6), 3.88 (ddd, J = 7.3, 6.1, 2.2 Hz, 1H, H-5), 3.55 (d, J = 7.9 Hz, 1H, H-2), 3.53 (s, 3H, Me), 1.52 (s, 3H), 1.36 (s, 3H), 1.07 (s, 9H). ^{13}C NMR (126 MHz, $CDCl_3$) δ 135.63, 135.58, 133.42, 133.31, 129.73, 127.71, 127.64, 110.06, 103.26, 78.62, 74.00, 73.59, 73.20, 62.60, 60.42, 56.90, 28.22, 26.73, 26.31, 19.22, 14.21. HRMS: $[M+Na]^+$ $C_{26}H_{36}NaO_6Si$ calcd 495.2179, obsd 495.2197.

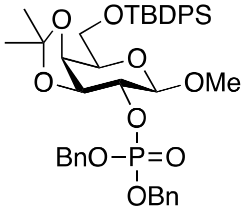

Methyl 6-*O*-(*tert*-butyldiphenylsilyl)-2-*O*-(dibenzylphosphono)-3,4-*O*-isopropylidene-β-D-galactopyranoside (23)

Compound **23** was prepared from compound **22** (4.86 g, 10.2 mmol) and phosphoramidite **8** (3.6 ml, 10.3 mmol) by following the general procedure for one-pot phosphorylation, providing the product in 93% yield. $[\alpha]^{25}_{D}$ 7.71 (c = 1, CH_2Cl_2); ^{1}H NMR (500 MHz, $CDCl_3$) δ 7.74 – 7.66 (m, 4H), 7.48 – 7.24 (m, 16H), 5.11 (dd, *J* = 9.7, 7.2 Hz, 4H, Bn), 4.37 – 4.21 (m, 4H, H-1, H-2, H-3, H-4), 3.99 – 3.91 (m, 2H, H-6), 3.88 (ddd, *J* = 7.2, 6.0, 2.0 Hz, 1H, H-5), 3.44 (s, 3H, Me), 1.49 (s, 3H), 1.33 (s, 3H), 1.06 (s, 9H). ^{13}C NMR (126 MHz, $CDCl_3$) δ 136.20, 136.14, 135.61, 135.57, 133.33, 133.26, 129.74, 128.56, 128.43, 128.41, 128.27, 128.23, 127.95, 127.92, 127.78, 127.72, 127.65, 110.43, 101.54, 101.50, 79.22, 79.17, 77.94, 77.91, 73.56, 73.34, 69.09, 69.05, 69.03, 68.99, 62.52, 60.41, 56.52, 27.85, 26.71, 26.37, 25.76, 21.08, 19.20, 14.21. HRMS: $[M+Na]^+$ $C_{40}H_{49}NaO_9PSi$ calcd 755.2781, obsd 755.2812.

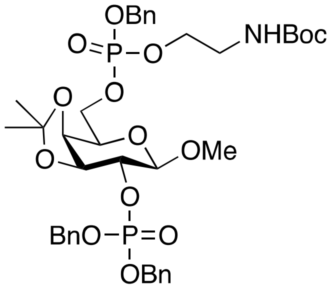

Methyl 2-*O*-(dibenzylphosphono)-6-*O*-((benzyl)-(2-(*tert*-butoxycarbonylamido)ethyl)-phospono)-3,4-*O*-isopropylidene-*β*-D-galactopyranoside (25)

Compound **23** (7 g, 9.55 mmol) was firstly applied to the general reaction for TBDPS removal. The formed crude product was directedly used as the starting material for the synthesis of compound **25** by following the general procedure of the one-pot phosphorylation reaction, in the presence of freshly made phosphoramidite **9**, provide the desired compound in 60% yield in 2 steps. $[\alpha]^{25}_{D}$ 14.95 (c = 1, CH_2Cl_2); ^{1}H NMR (500 MHz, $CDCl_3$) δ 7.46 – 7.29 (m, 15H), 5.14 – 5.07 (m, 6H), 4.96 (s, 1H, NH), 4.36 – 4.18 (m, 5H, H-1, H-2, H-3, H-6), 4.08 (dt, *J* = 7.5, 5.2 Hz, 3H, H-4, OCH_2), 3.95 (ddt, *J* = 6.3, 4.6, 2.8 Hz, 1H, H-5), 3.44 (dd, *J* = 4.1, 0.8 Hz, 3H, Me), 3.37 (q, *J* = 5.5 Hz, 2H, CH_2N), 1.48 (s, 3H), 1.44 (s, 9H), 1.30 (d, *J* = 2.5 Hz, 3H). ^{13}C NMR (126 MHz, $CDCl_3$) δ 136.02, 128.83, 128.71, 128.44, 128.33, 128.31, 128.14, 127.91, 127.78, 110.89, 101.35, 78.48, 77.69, 73.21, 71.43, 69.65, 69.15, 69.11, 66.21, 56.73, 56.70, 28.36, 27.65, 26.35. HRMS: $[M+Na]^+$ $C_{38}H_{51}NNaO_{14}P_2$ calcd 830.2785, obsd 830.2796.

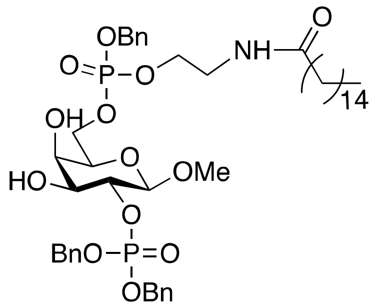

Methyl 2-*O*-(dibenzylphosphono)-6-*O*-((benzyl)-(2-palmitamidoethyl)-phospono)-β-D-galactopyranoside (19)

Compound **25** (4.6 g, 5.73 mmol) was applied to the general procedure of Boc removal, followed by the general procedure of amidation reaction with palmitic acid (1.6 g, 6.3 mmol), affording the desired compound in 63% yield in two steps. $[\alpha]^{25}_{D}$ -19.09 (c = 0.1, CH_2Cl_2); ^1H NMR (500 MHz, CD_3OD) δ 7.54 – 7.29 (m, 15H), 5.17 – 5.08 (m, 6H, Bn), 4.40 – 4.31 (m, 1H, H-1), 4.26 – 4.16 (m, 2H, H-6), 4.10 (q, *J* = 7.2 Hz, 2H, OCH_2), 3.83 (t, *J* = 4.1 Hz, 1H, H-5), 3.81 – 3.68 (m, 3H, H-4, H-3, H-2), 3.47 – 3.40 (m, 5H, CH_2N, Me), 2.18 (td, *J* = 7.6, 2.1 Hz, 2H), 1.63 – 1.55 (m, 2H), 1.34 – 1.25 (m, 24H), 0.96 – 0.80 (m, 3H). ^{13}C NMR (126 MHz, CD_3OD) δ 175.27, 136.05, 128.46, 128.36, 128.16, 128.14, 128.07, 128.05, 127.89, 127.61, 127.43, 102.09, 78.65, 73.17, 71.99, 69.54, 69.25, 69.13, 69.08, 68.95, 66.30, 55.80, 39.19, 37.46, 35.64, 31.67, 29.39, 29.37, 29.36, 29.24, 29.08, 28.92, 27.33, 25.56, 22.34, 13.05. HRMS: $[M+H]^+$ $C_{46}H_{70}NO_{13}P_2$ calcd 906.4322, obsd 906.4331.

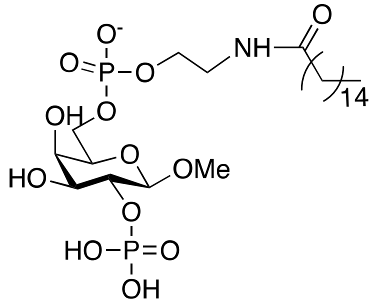

Methyl 2-*O*-phosphono-6-*O*-((2-palmitamidoethyl)-phospono)-*β*-D-galactopyranoside (5)

Compound **5** was synthesized from **19** in quantitative yield following the general procedure of global debenzylation reaction. $[\alpha]^{25}_{D}$ -2.35 (c = 1, CH_2Cl_2); ^1H NMR (500 MHz, CD_3OD : $CDCl_3$: D_2O = 4:2:1) δ 4.35 (d, *J* = 7.6 Hz, 1H, H-1), 4.17 – 4.02 (m, 3H, H-2, H-3, H-4), 3.97 (q, *J* = 4.3 Hz, 3H, H-5, $POCH_2$), 3.80 – 3.63 (m, 2H, H-6),), 3.52 (s, 3H, OCH_3), 3.42 (t, *J* = 5.4 Hz, 2H, CH_2N), 2.24 – 2.18 (m, 2H, $COCH_2$), 1.58 (p, *J* = 7.2 Hz, 2H, $COCH_2CH_2$), 1.26 (d, *J* = 5.1 Hz, 24H), 0.87 (t, *J* = 6.9 Hz, 3H). ^{13}C NMR (126 MHz, CD_3OD : $CDCl_3$: D_2O = 4:2:1) δ 176.66, 103.57, 103.54, 77.83, 74.28, 73.23, 69.19, 65.79, 65.48, 63.72, 57.82, 49.51, 49.34, 49.17, 49.00, 48.83, 48.66, 48.49, 40.67, 39.04, 39.02, 37.00, 32.76, 30.54, 30.53, 30.48, 30.45, 30.32, 30.19, 28.76, 26.72, 23.49, 14.69, 14.67. HRMS: $[M-H]^-$ $C_{25}H_{50}NO_{13}P_2$ calcd 634.2757, obsd 634.2767.

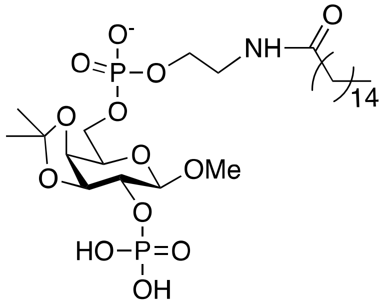

Methyl 2-*O*-phosphono-6-*O*-((2-palmitamidoethyl)-phospono)-3,4-*O*-isopropylidene-β-D-galactopyranoside (6)

Compound **19** (500 mg, 0.56 mmol) was dissolved in acetone, then 2,2-dimethoxy propane (0.08 mL, 0.67 mmol) and camphorsulfonic acid (20 mg, 0.12 mmol) was added. After stirred at room temperature overnight, Et_3N was added to neutralize the pH around 7. The reaction mixture was concentrated under vacuum, and the residue was diluted with DCM and washed with aqueous $NaHCO_3$ solution and saturated NaCl solution successively. The organic layer was collected and dried over anhydrous Na_2SO_4. The volatiles were removed under vacuum and the resulted residue was purified by silica gel column chromatography to afford isopropylidene protected intermediate, which was applied to the general procedure of global debenzylation, and provided the desired product in 58% yield in two steps. $[\alpha]^{25}_D$ 0.19 (c = 3, CH_2Cl_2); ^{1}H NMR (500 MHz, CD_3OD : $CDCl_3$: D_2O = 4:2:1) δ4.74 (m, 1H, H-4) 4.58 (dd, *J* = 7.2, 4.0 Hz, 1H, H-2), 4.35 (dd, *J* = 7.1, 1.9 Hz, 1H, H-1), 4.13 (td, *J* = 6.6, 2.9 Hz, 2H, H-3, H-5), 4.06 – 3.96 (m, 2H, H-6), 3.92 (dt, *J* = 7.0, 5.3 Hz, 2H, $POCH_2$), 3.46 (s, 3H, Me), 3.41 (t, *J* = 5.3 Hz, 2H, CH_2N), 2.23 – 2.18 (m, 2H, $COCH_2$), 1.58 (d, *J* = 8.9 Hz, 2H), 1.25 (d, *J* = 3.6 Hz, 24H), 0.87 (t, *J* = 6.9 Hz, 3H). ^{13}C NMR (126 MHz, CD_3OD : $CDCl_3$: D_2O = 4:2:1) δ 173.07, 105.93, 103.25, 68.82, 68.10, 59.29, 49.51, 49.34, 49.17, 49.00, 48.83, 48.79, 48.66, 48.49, 47.73, 43.47, 43.42, 32.63, 30.36, 30.32, 27.49, 26.64, 25.24, 23.35, 20.15, 20.09, 19.18, 14.52, 5.57. HRMS: $[M-H]^-$ $C_{28}H_{54}NO_{13}P_2$ calcd 674.3070, obsd 674.3087.

^{1}H-NMR of **7** ($CDCl_3$, 500 MHz)

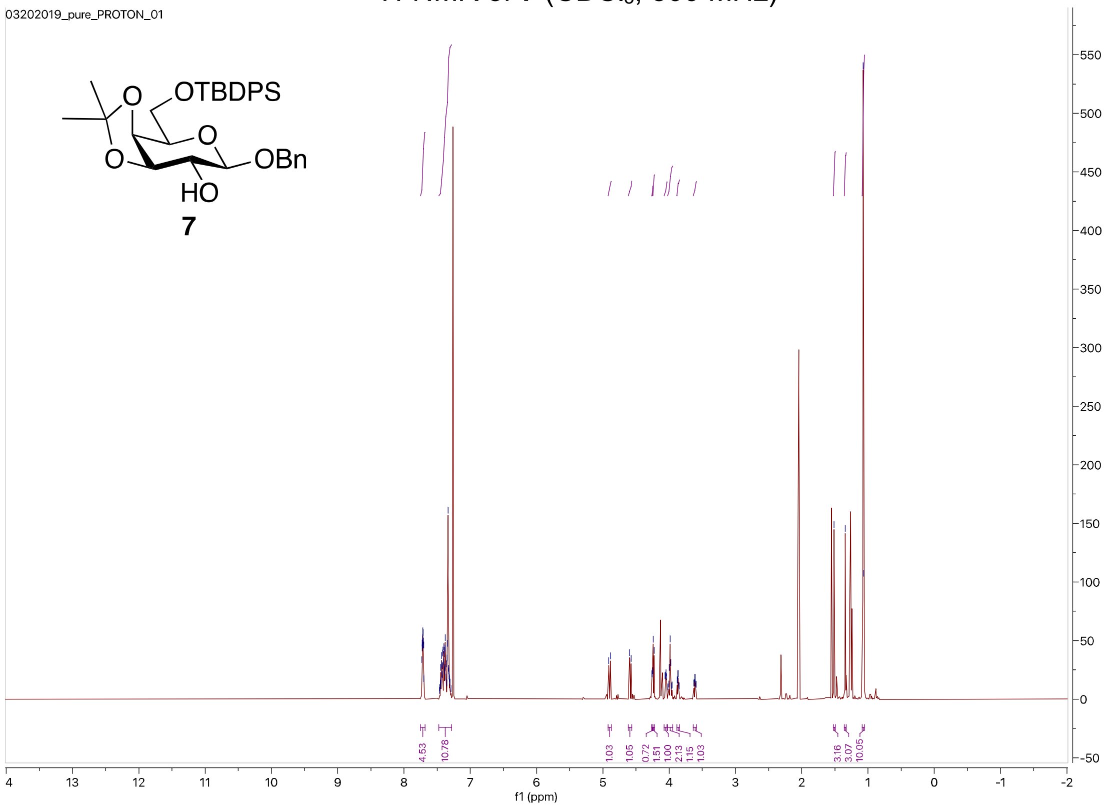

^{13}C-NMR of **7** ($CDCl_3$, 125 MHz)

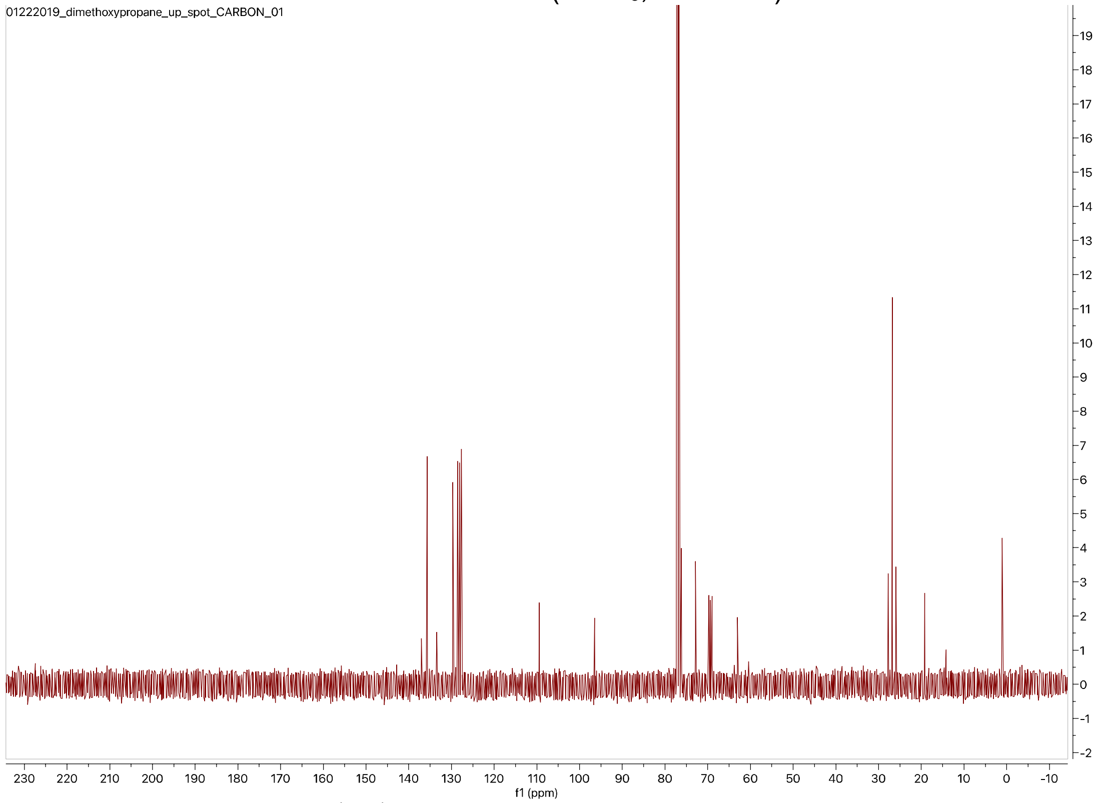

^{1}H-^{1}H gCOSY of **7** ($CDCl_3$, 500 MHz)

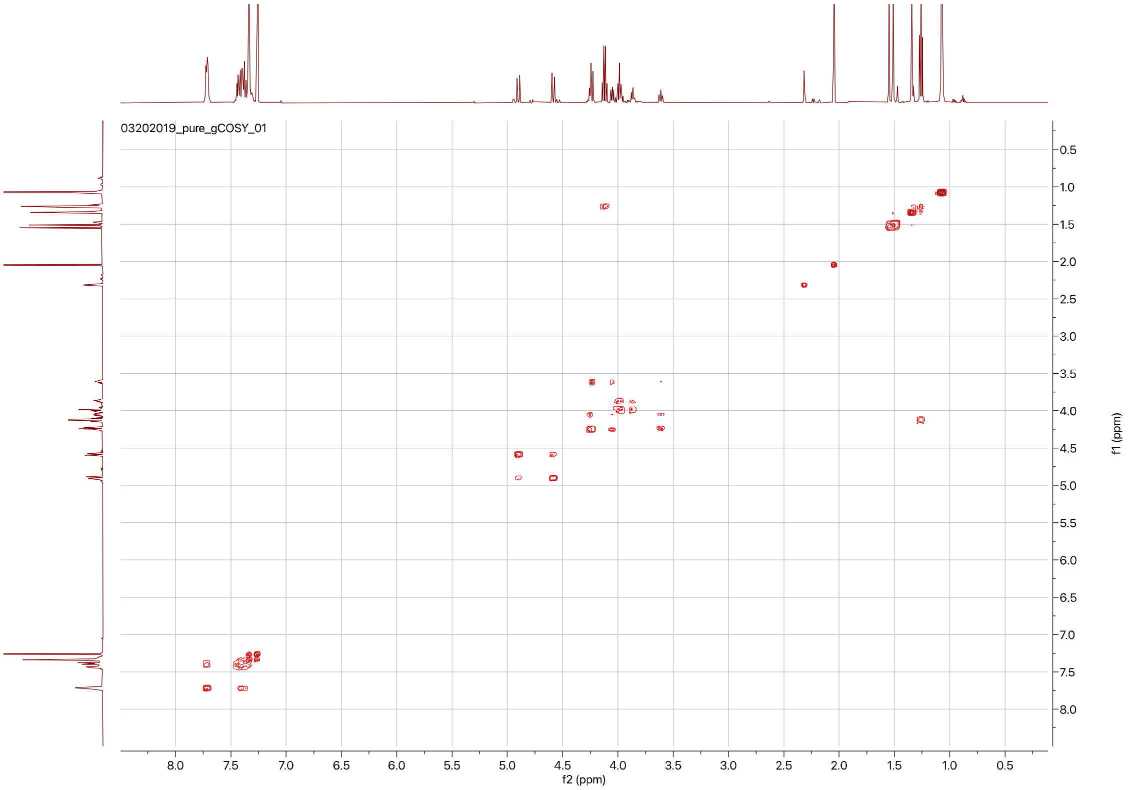

^{1}H-^{13}C gHSQCAD of **7** ($CDCl_3$, 500 MHz)

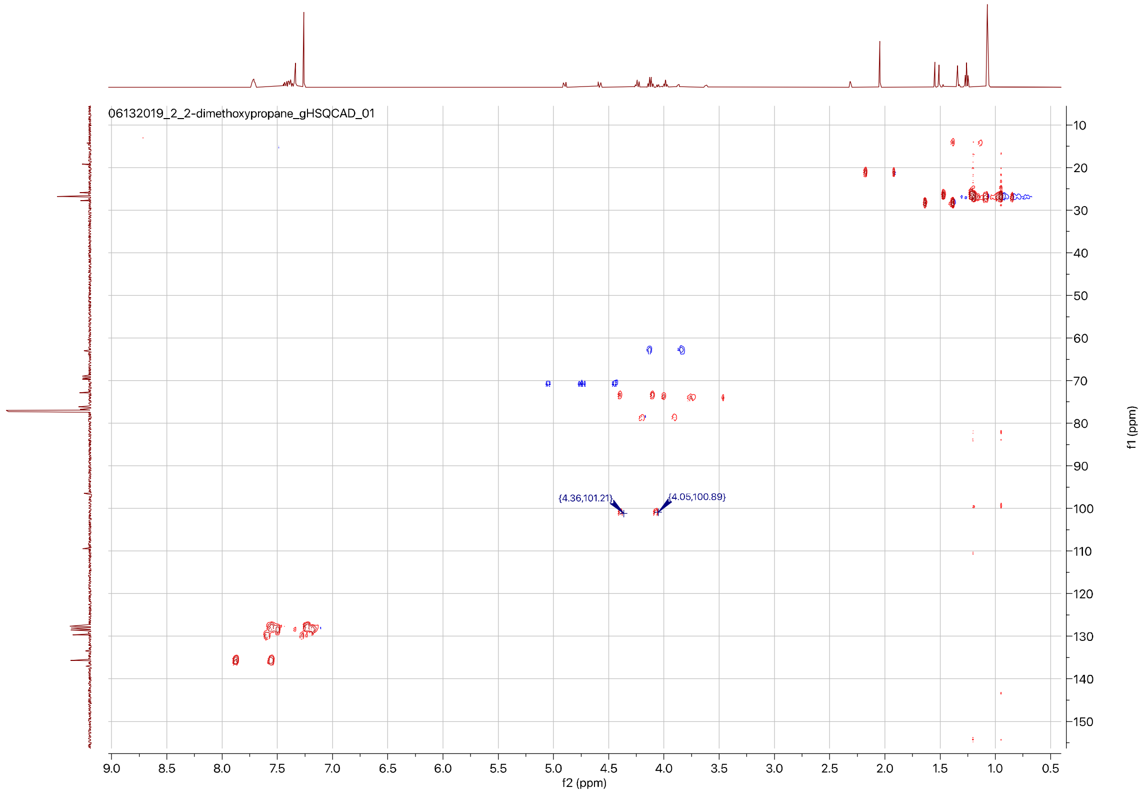

^{1}H-NMR of **14** ($CDCl_3$, 500 MHz)

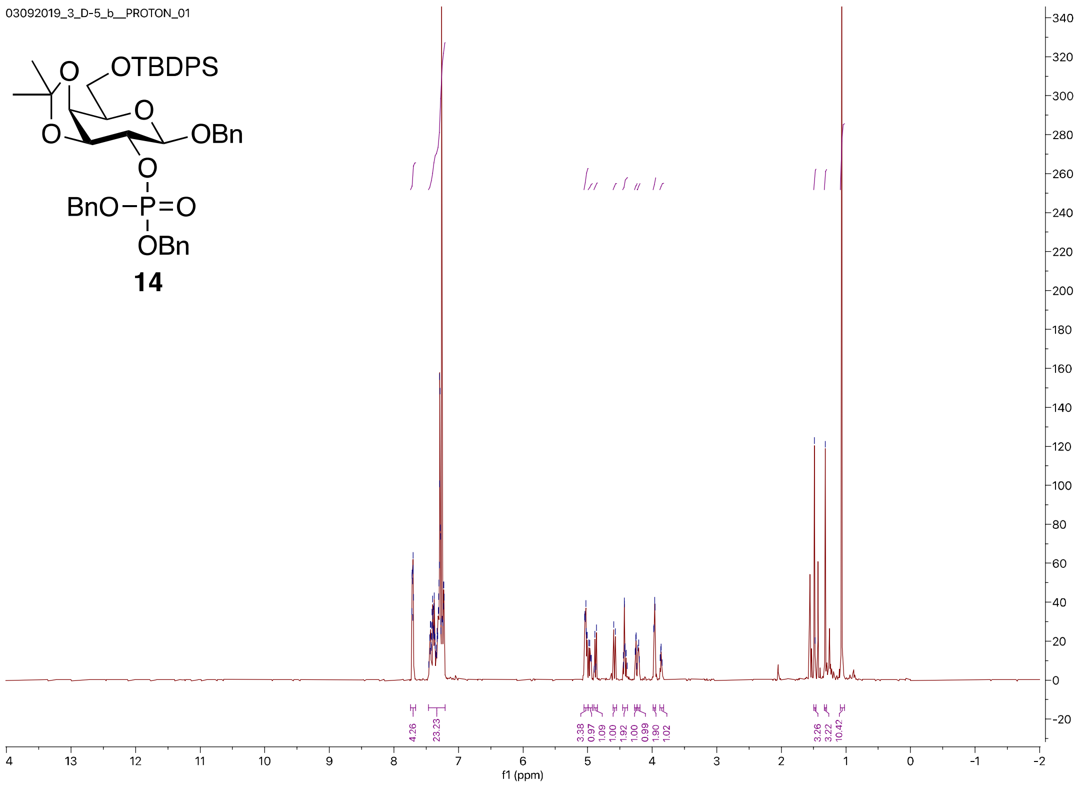

^{13}C-NMR of **14** ($CDCl_3$, 125 MHz)

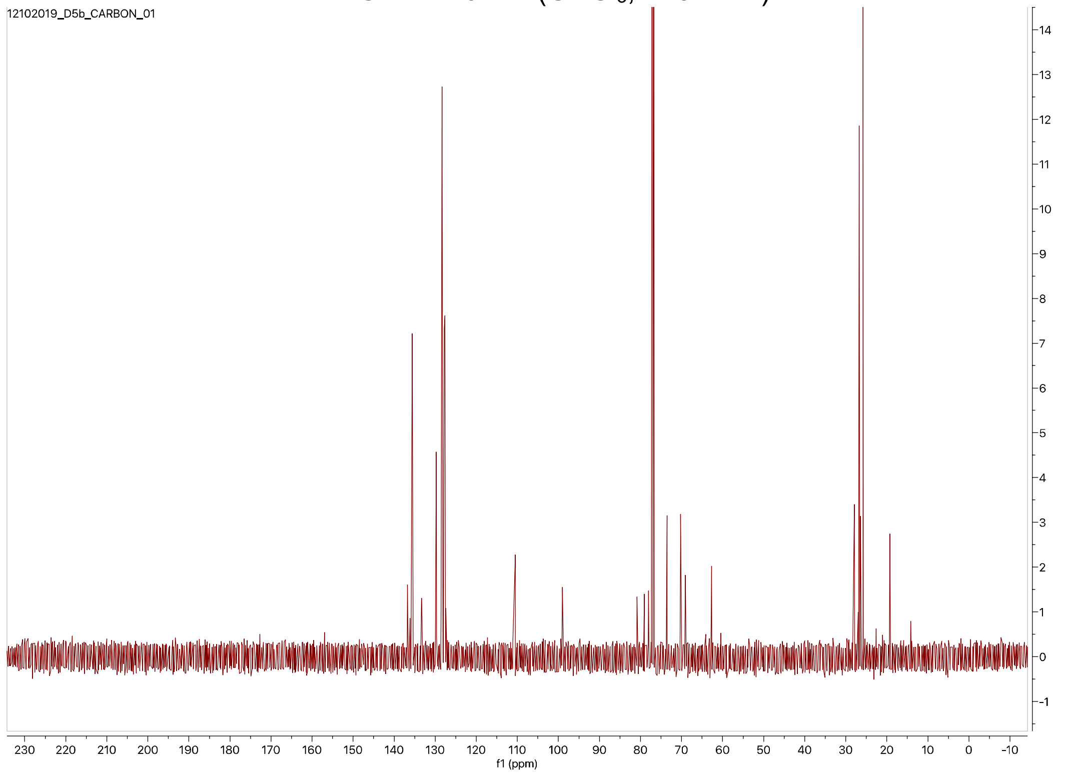

^{1}H-^{1}H gCOSY of **14** ($CDCl_3$, 500 MHz)

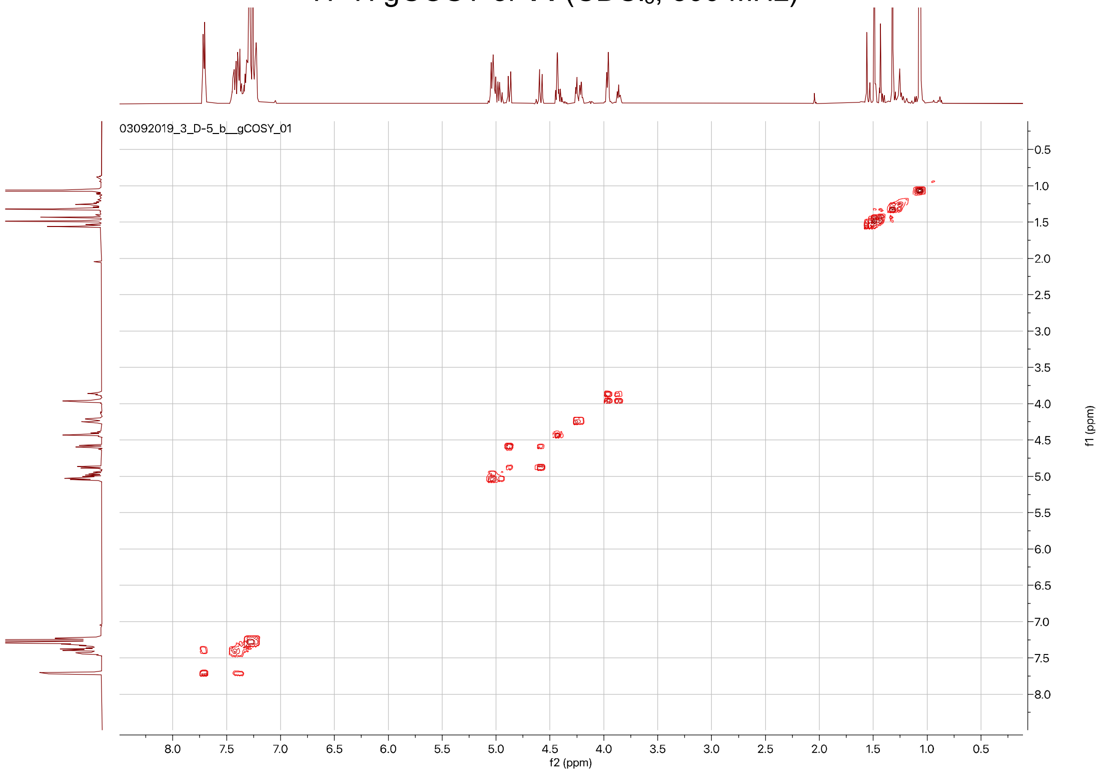

^{1}H-^{13}C gHSQCAD of **14** ($CDCl_3$, 500 MHz)

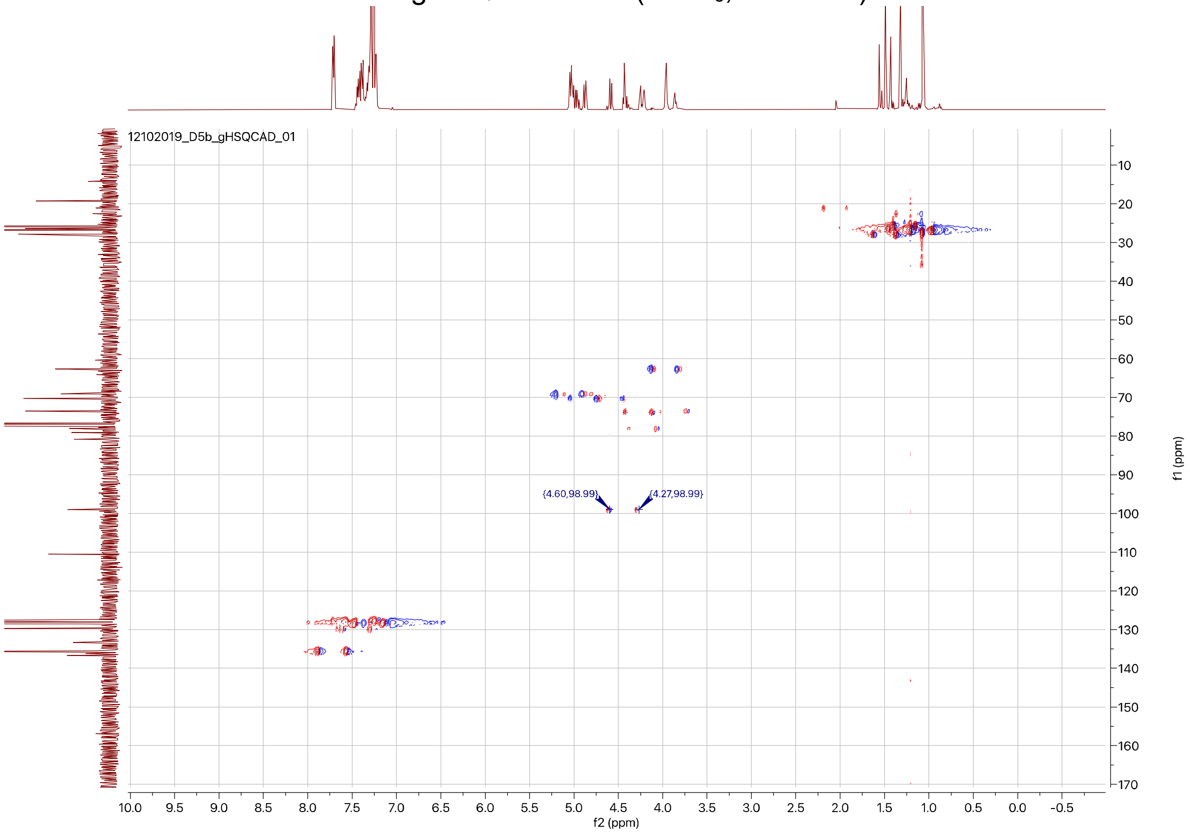

^{1}H-NMR of **16** ($CDCl_3$, 500 MHz)

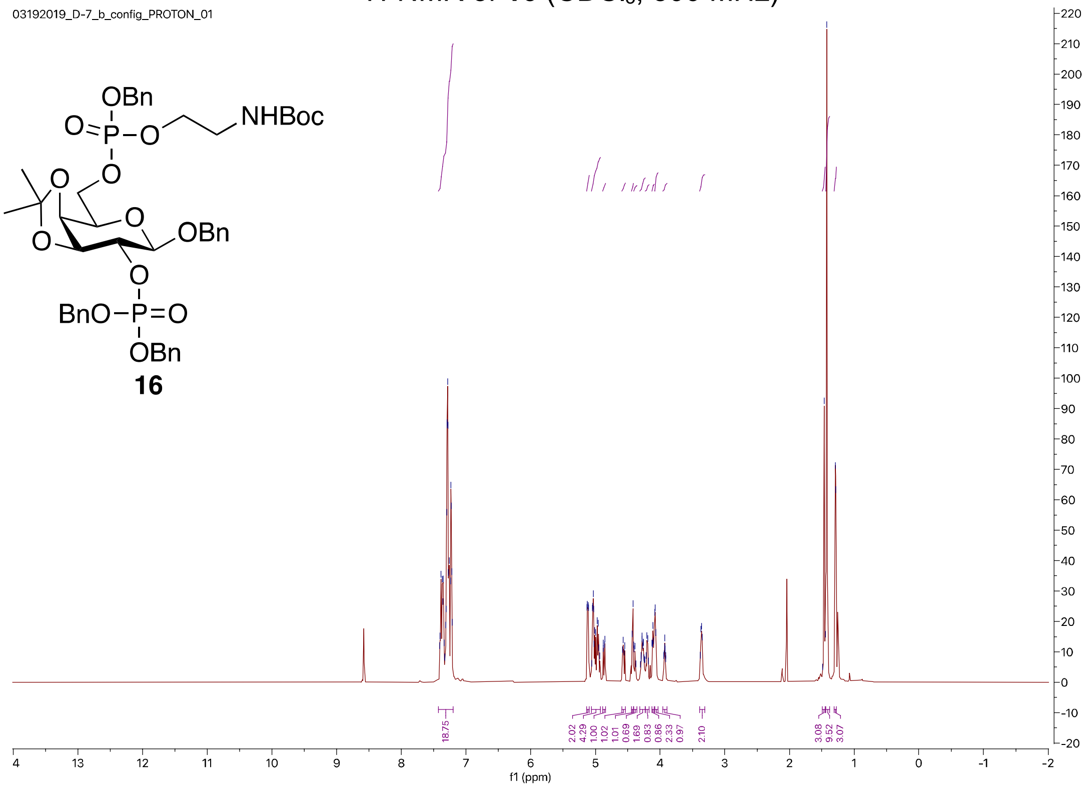

^{13}C-NMR of **16** ($CDCl_3$, 125 MHz)

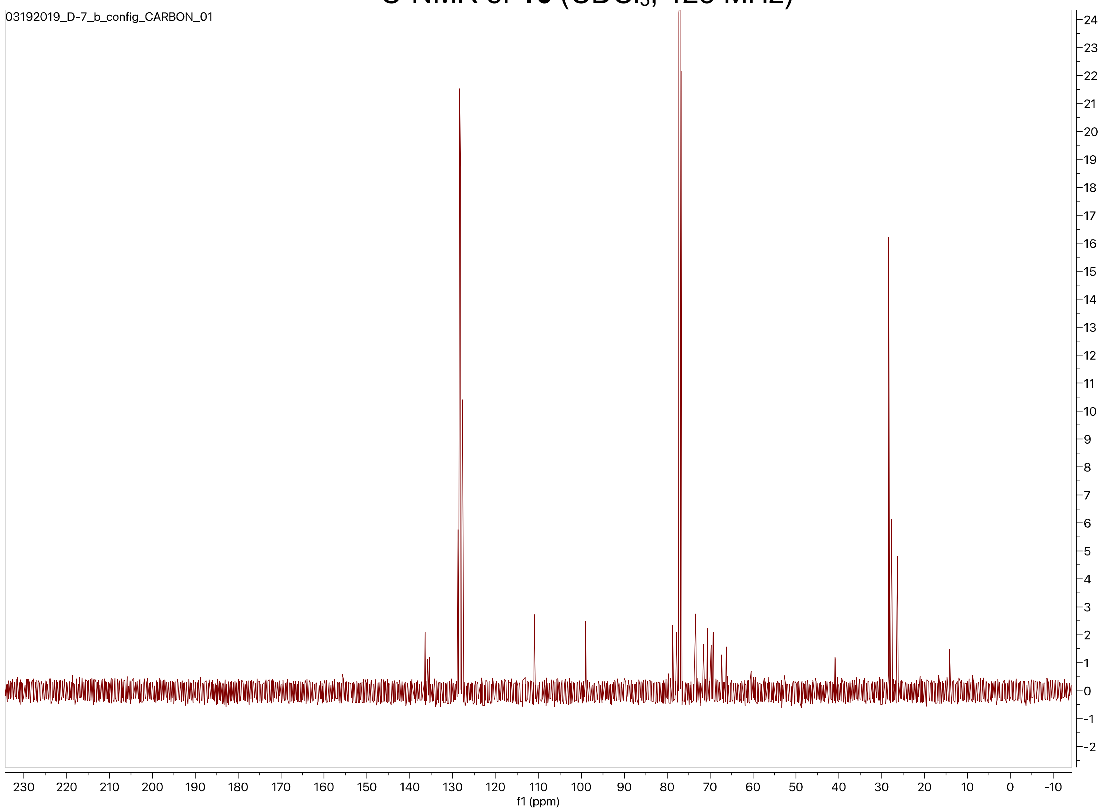

^{1}H-^{1}H gCOSY of **16** ($CDCl_3$, 500 MHz)

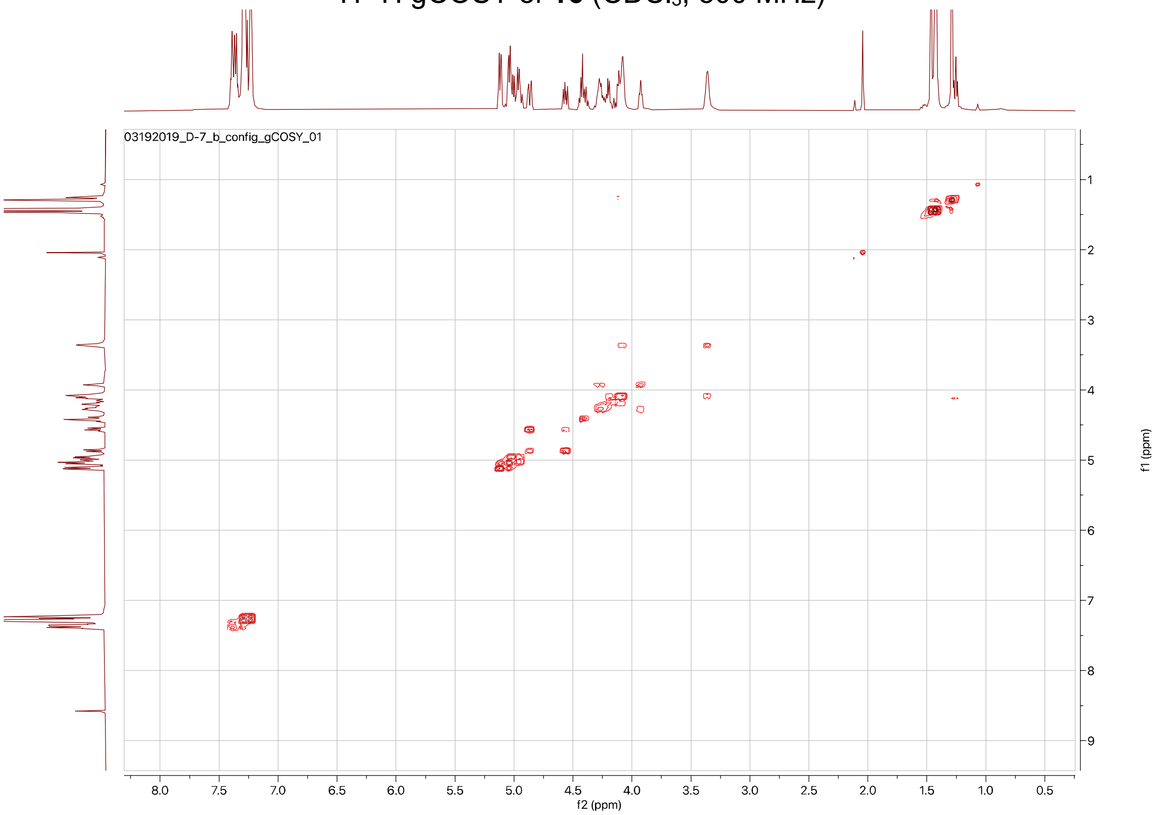

^{1}H-^{13}C gHSQCAD of **16** ($CDCl_3$, 500 MHz)

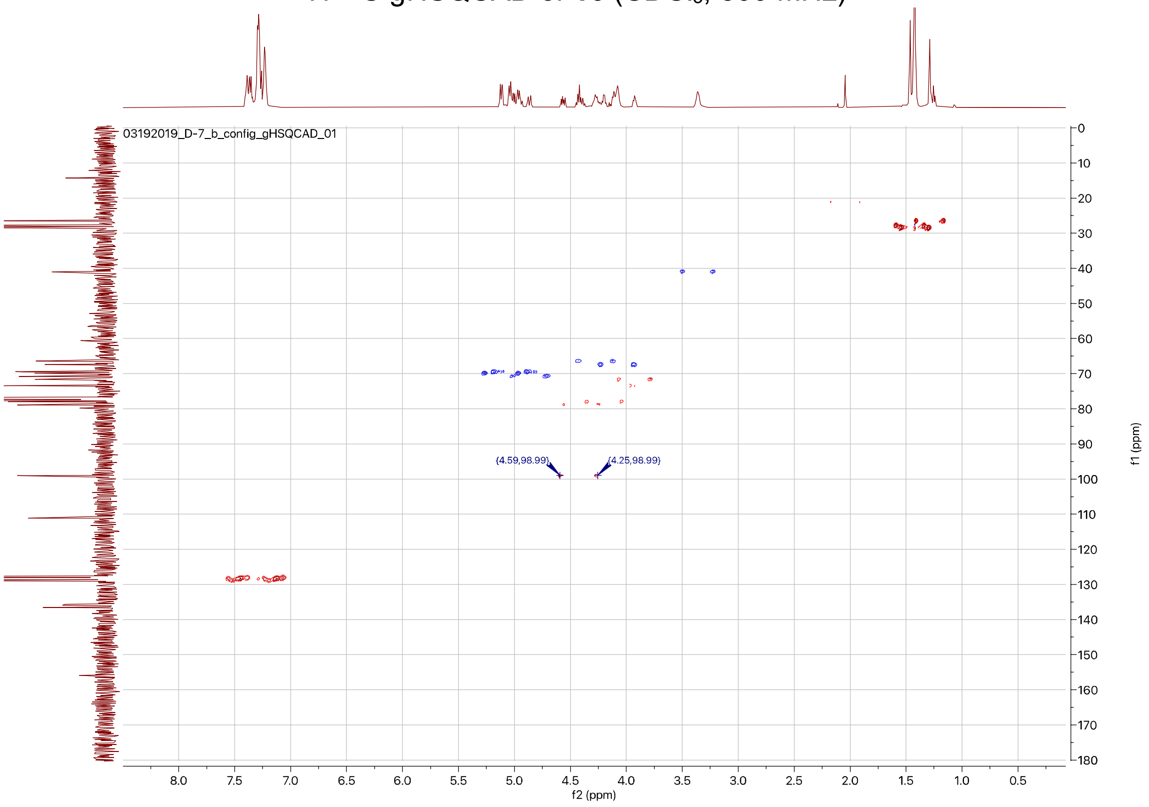

^{1}H-NMR of **17-1** ($CDCl_3$, 500 MHz)

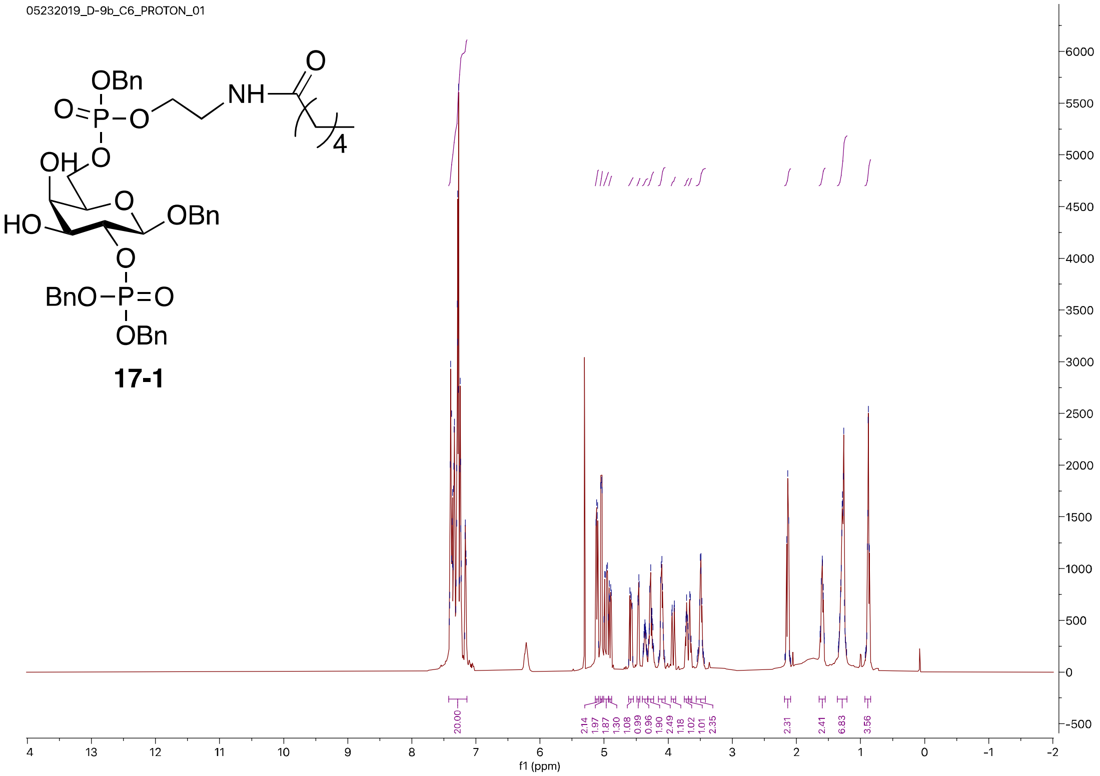

^{13}C-NMR of **17-1** ($CDCl_3$, 125 MHz)

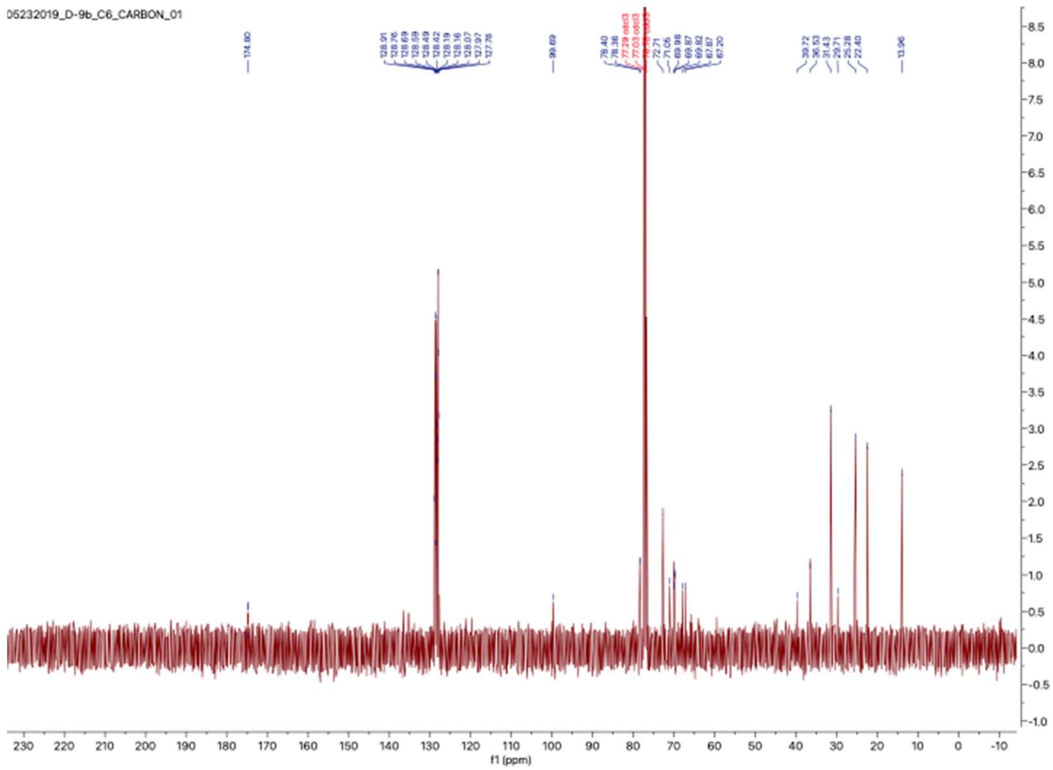

^{1}H-^{1}H gCOSY of **17-1** ($CDCl_3$, 500 MHz)

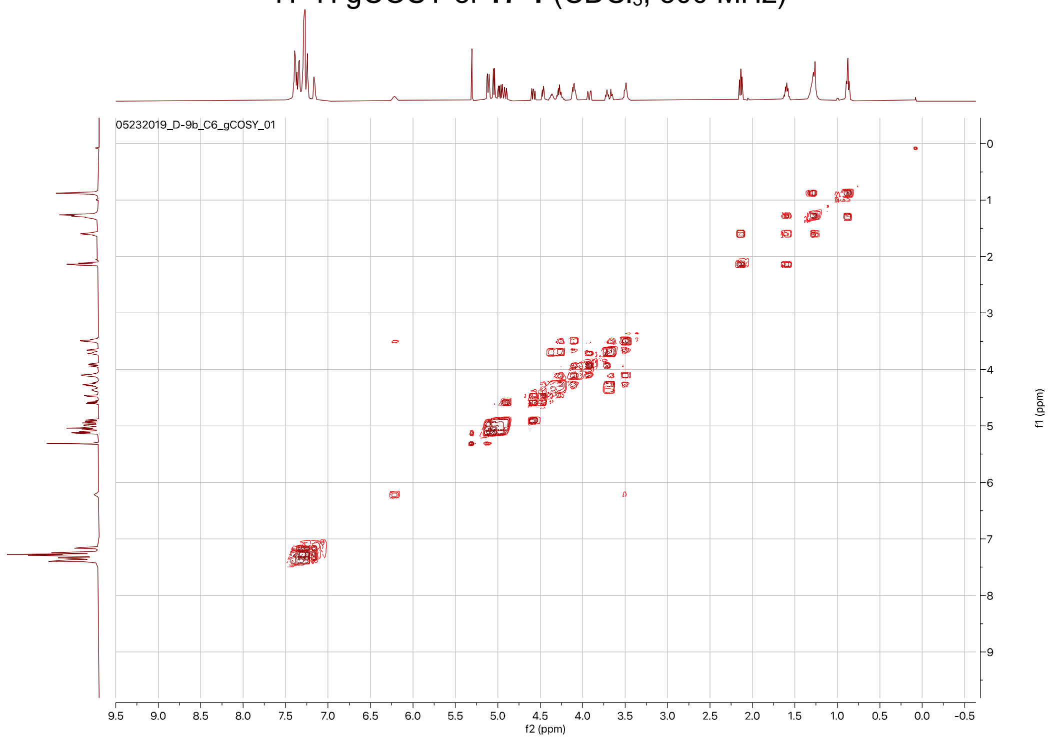

^{1}H-^{13}C gHSQCAD of **17-1** ($CDCl_3$, 500 MHz)

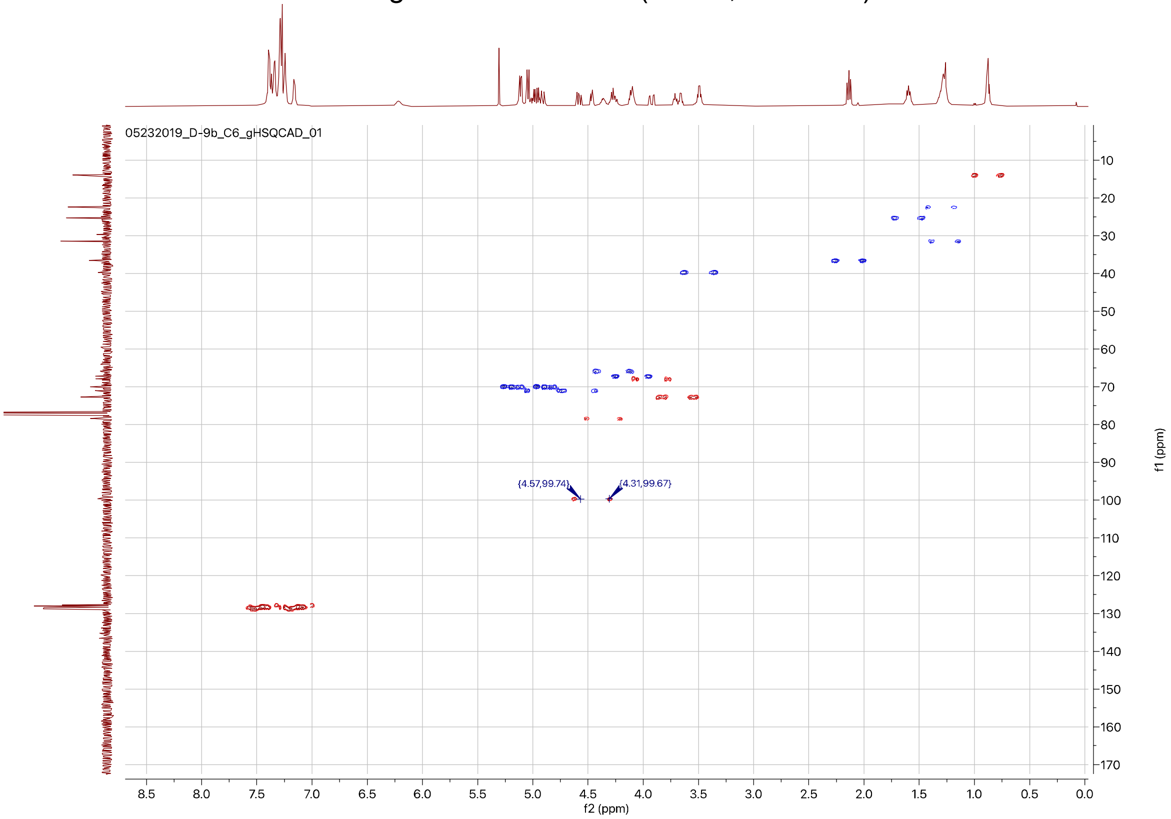

^{1}H-NMR of **17-2** ($CDCl_3$, 500 MHz)

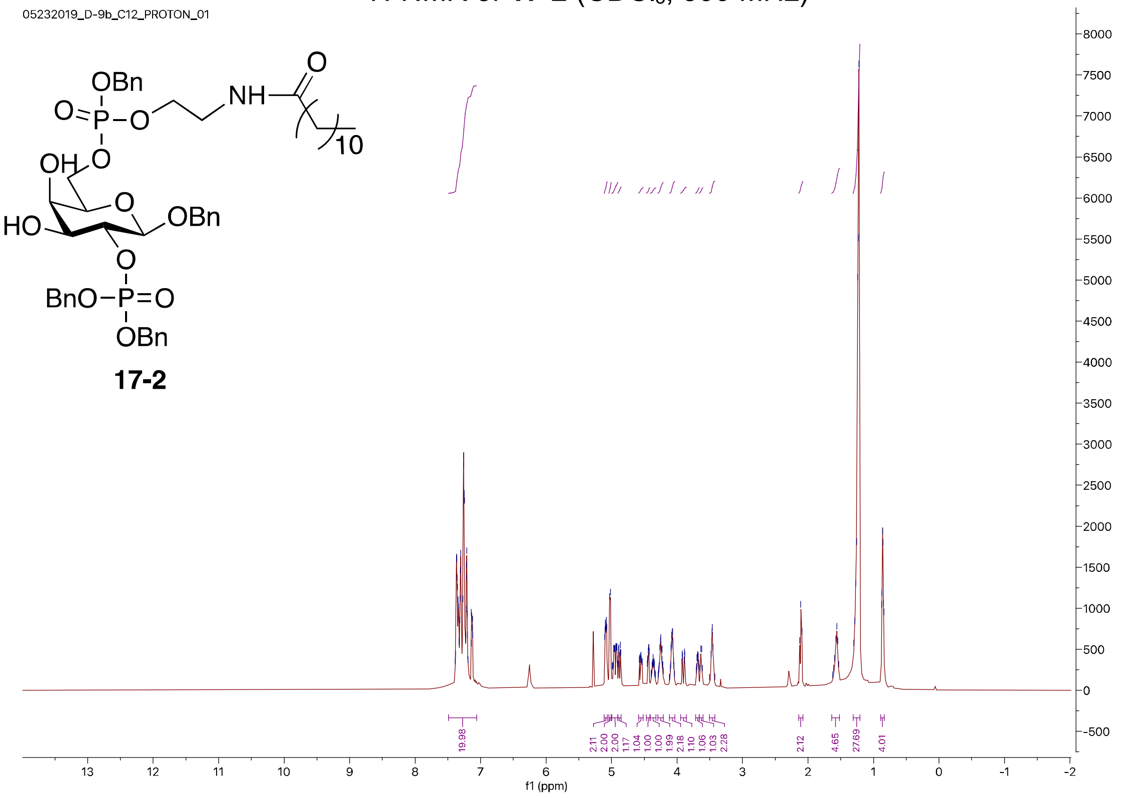

^{13}C-NMR of **17-2** ($CDCl_3$, 125 MHz)

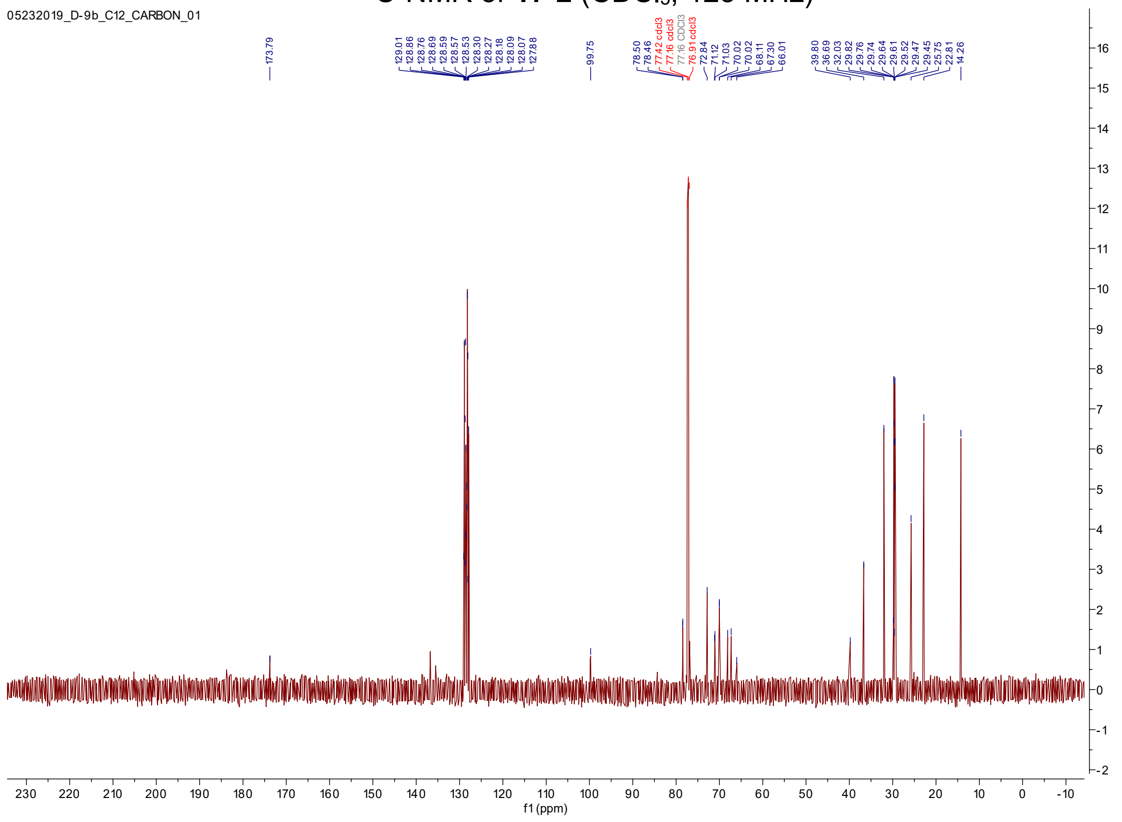

^{1}H-^{1}H gCOSY of **17-2** ($CDCl_3$, 500 MHz)

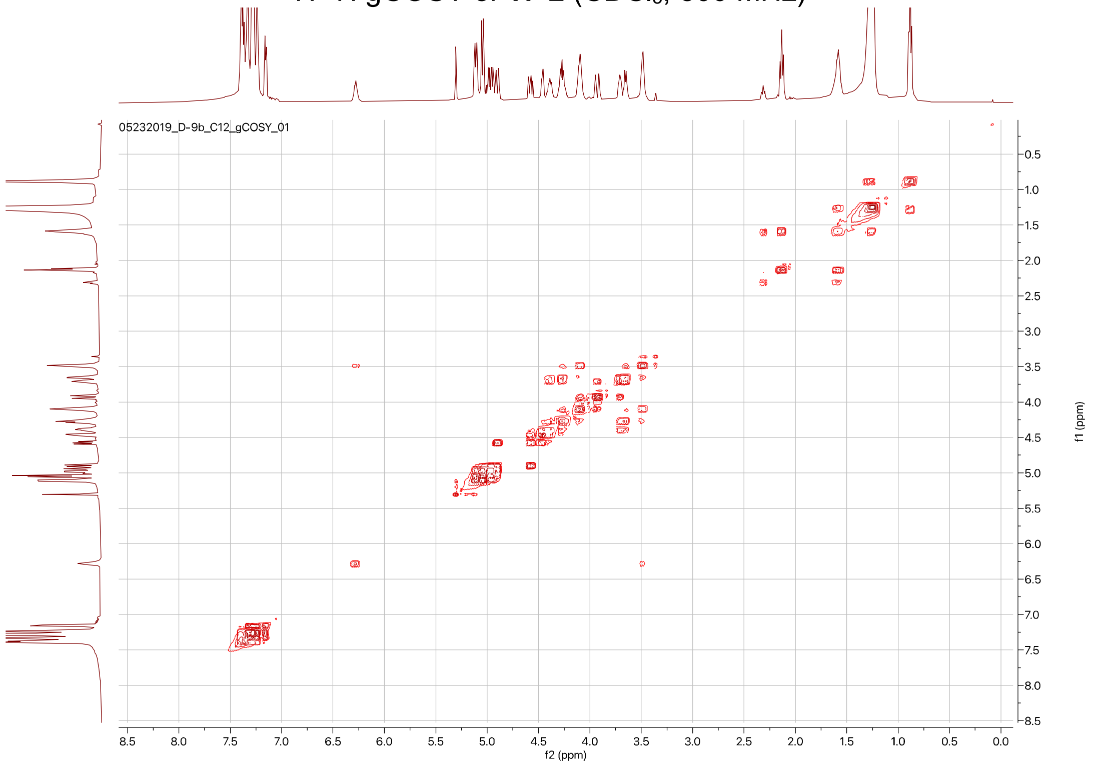

^{1}H-^{13}C gHSQCAD of **17-2** ($CDCl_3$, 500 MHz)

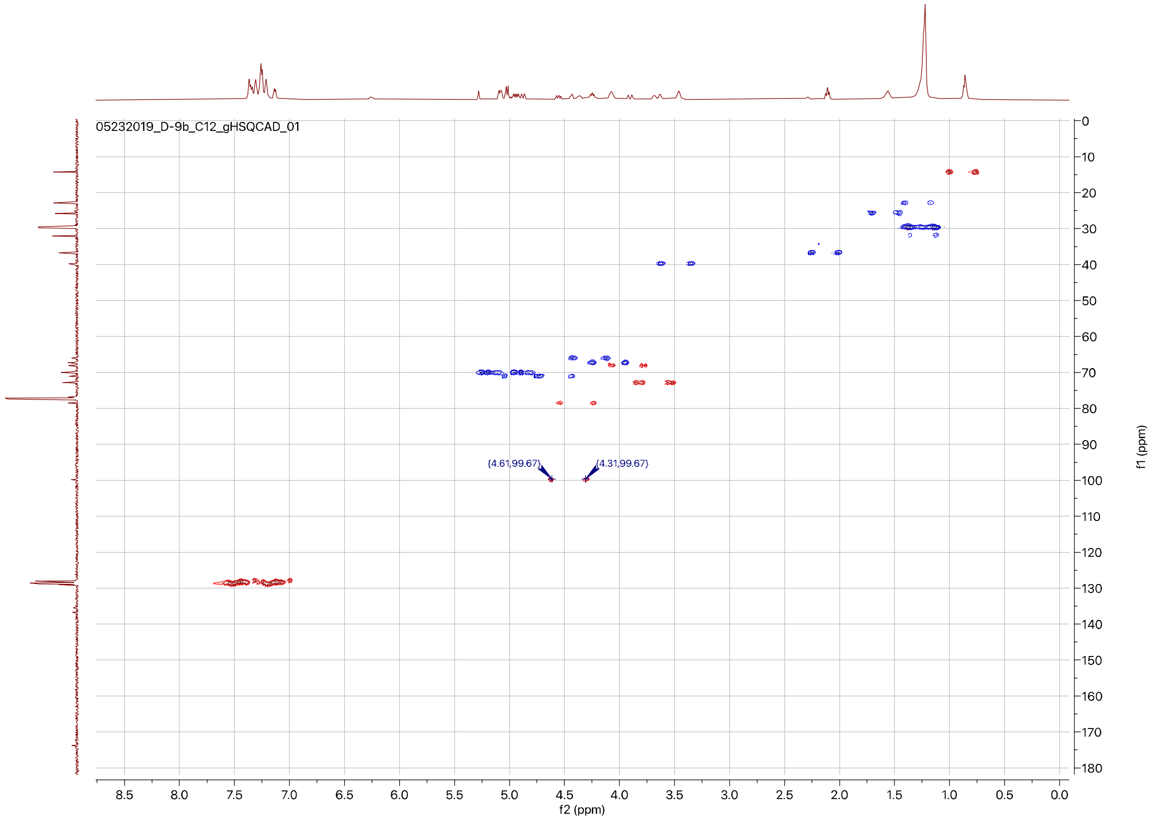

^{1}H-NMR of **17-3** ($CDCl_3$, 500 MHz)

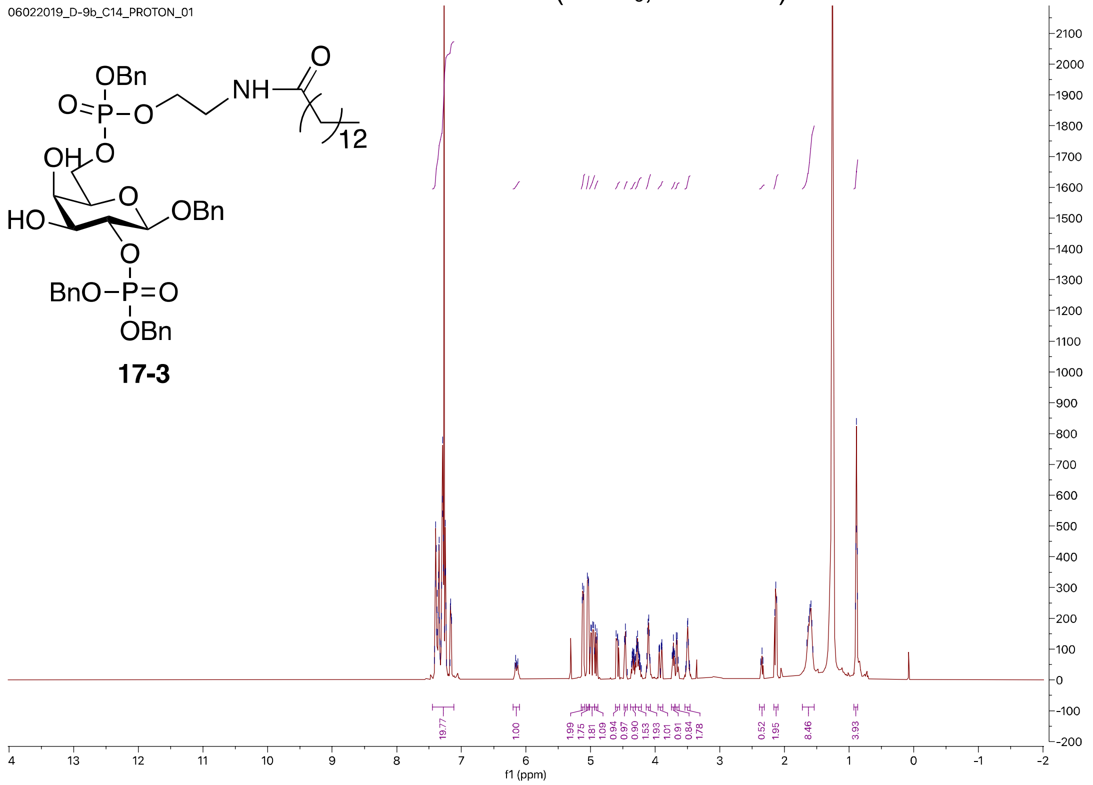

^{13}C-NMR of **17-3** ($CDCl_3$, 125 MHz)

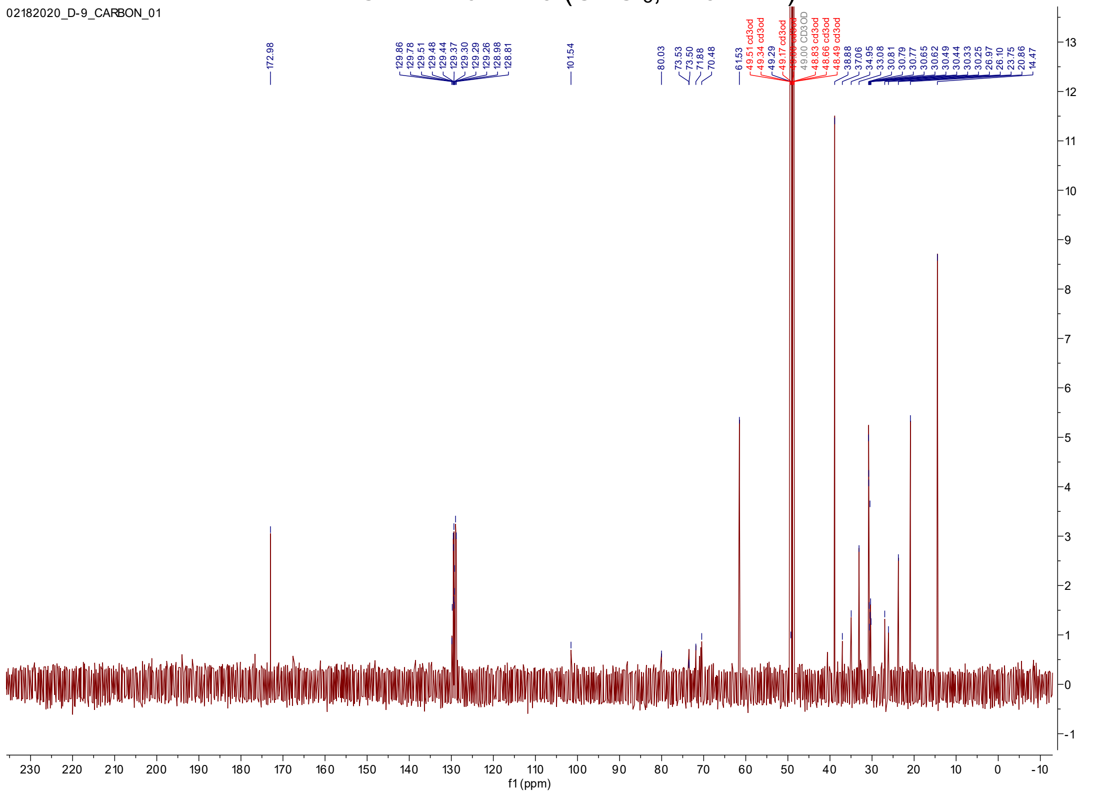

^{1}H-^{1}H gCOSY of **17-3** ($CDCl_3$, 500 MHz)

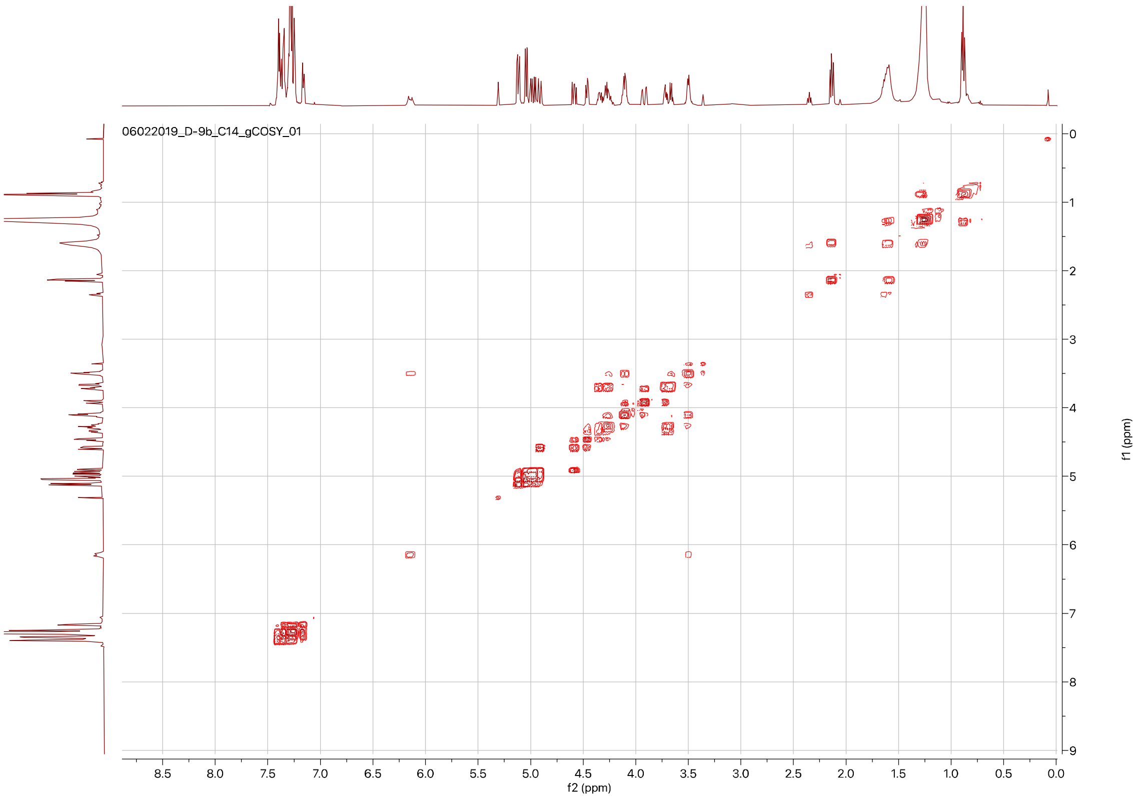

^{1}H-^{13}C gHSQCAD of **17-3** ($CDCl_3$, 500 MHz)

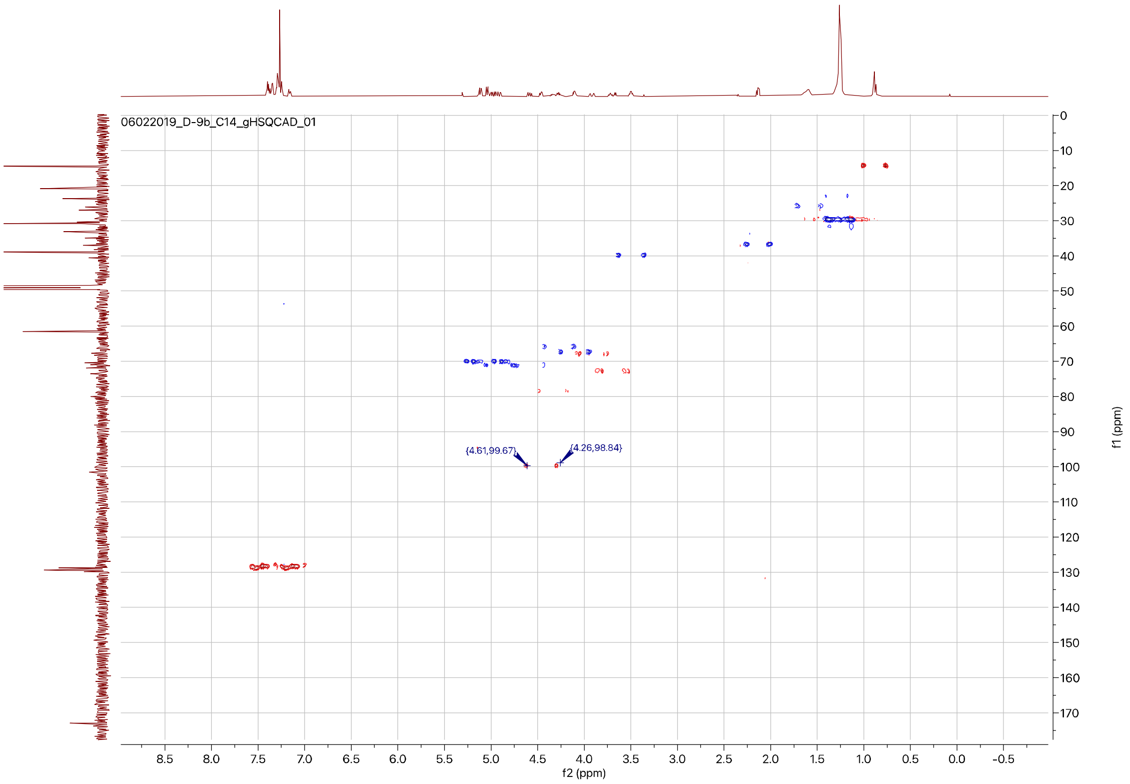

^{1}H-NMR of **17-4** ($CDCl_3$, 500 MHz)

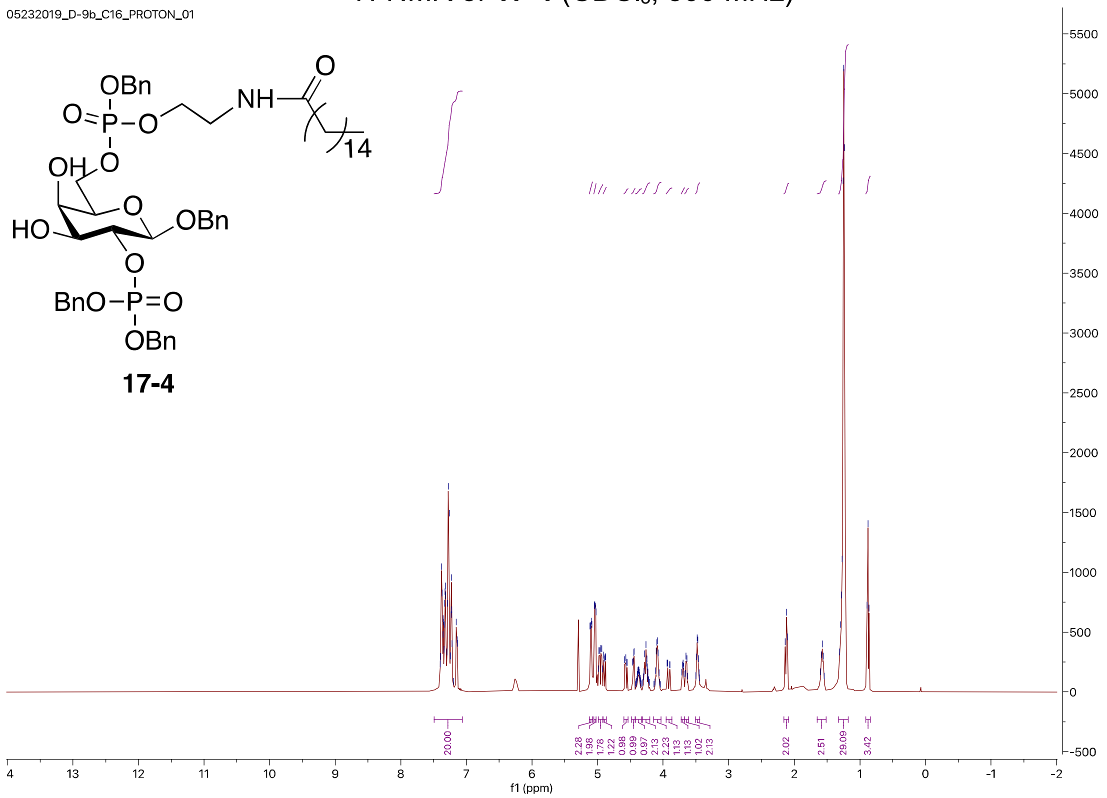

^{13}C-NMR of **17-4** ($CDCl_3$, 125 MHz)

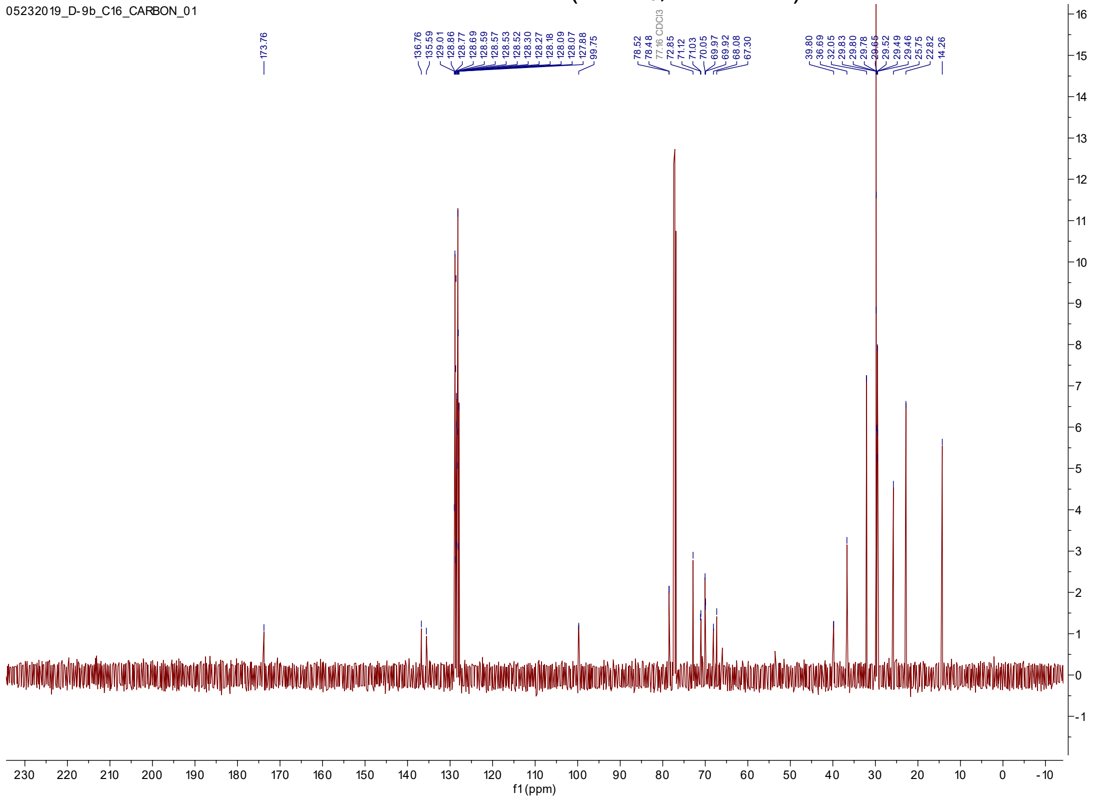

^{1}H-^{1}H gCOSY of **17-4** ($CDCl_3$, 500 MHz)

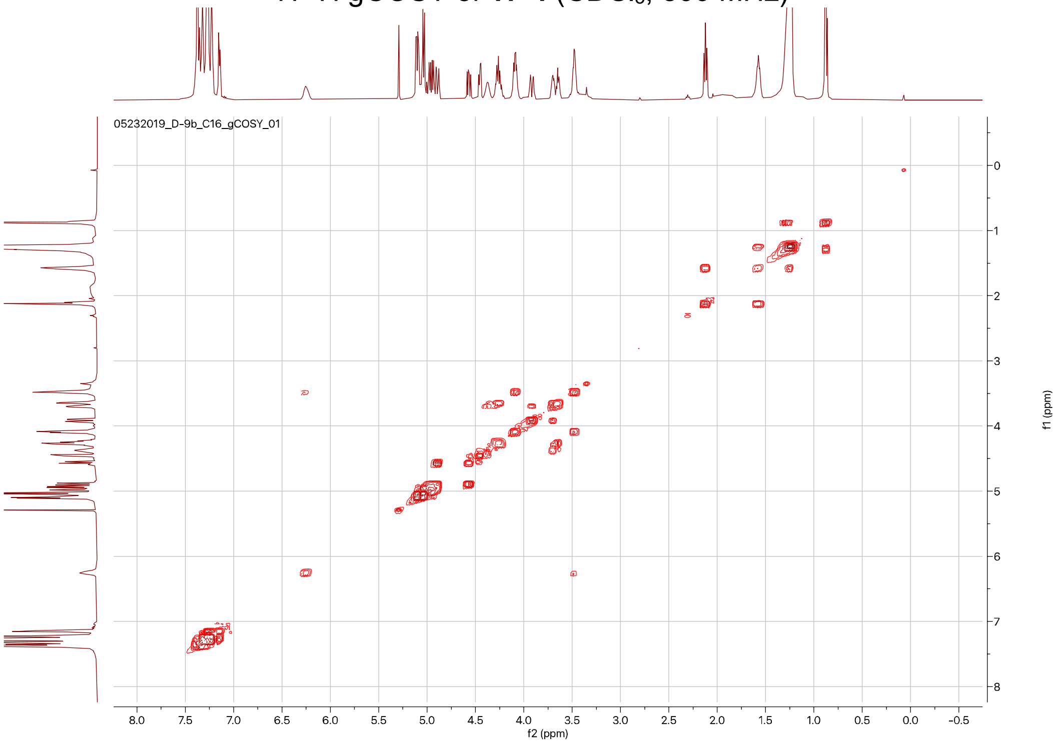

^{1}H-^{13}C gHSQCAD of **17-4** ($CDCl_3$, 500 MHz)

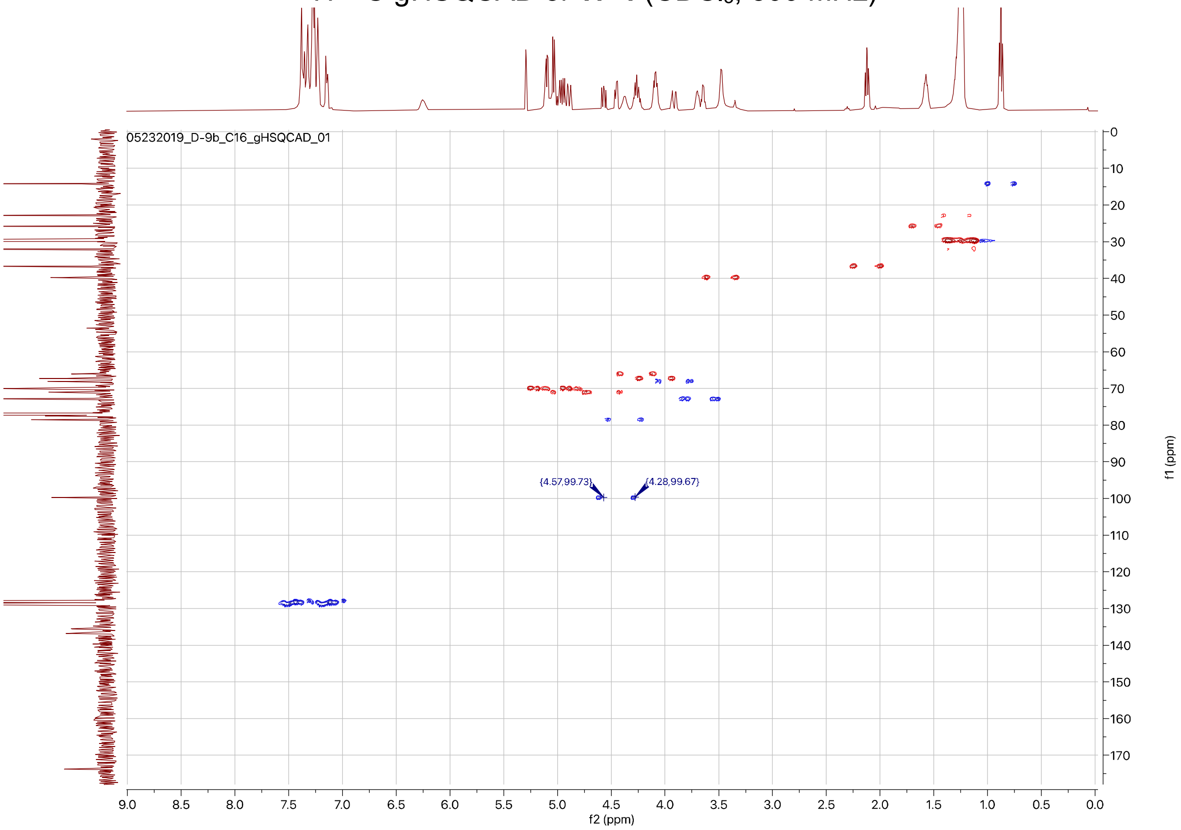

^{1}H-NMR of **1** ($CDCl_3$: CD_3OD : D_2O 1 : 2 : 0.5, 500 MHz)

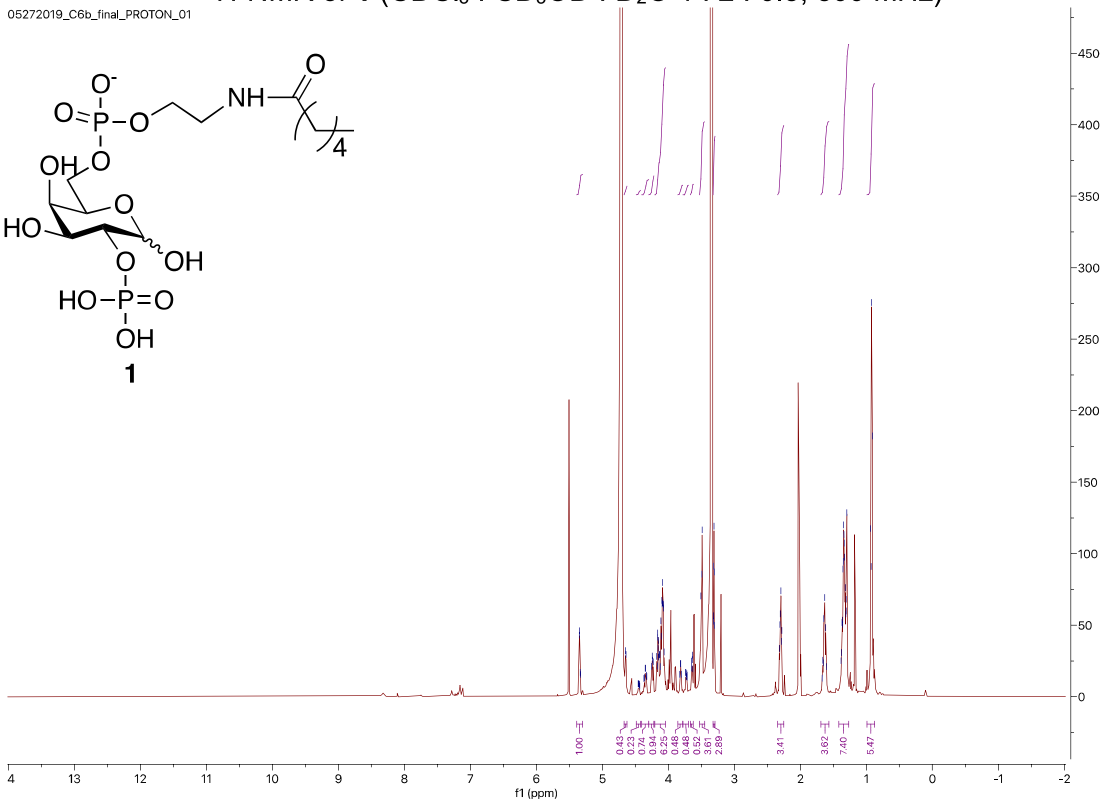

^{13}C-NMR of **1** ($CDCl_3$: CD_3OD : D_2O 1 : 2 : 0.5, 125 MHz)

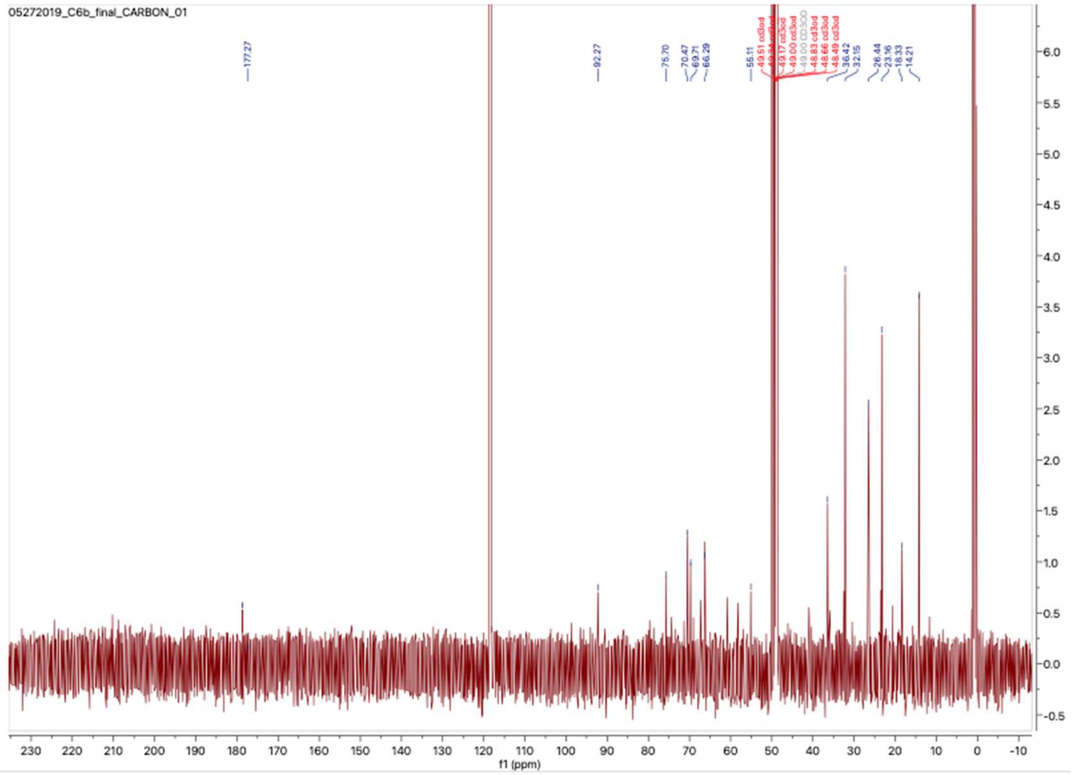

^{1}H-NMR of **2** ($CDCl_3$: CD_3OD : D_2O 1 : 2 : 0.5, 500 MHz)

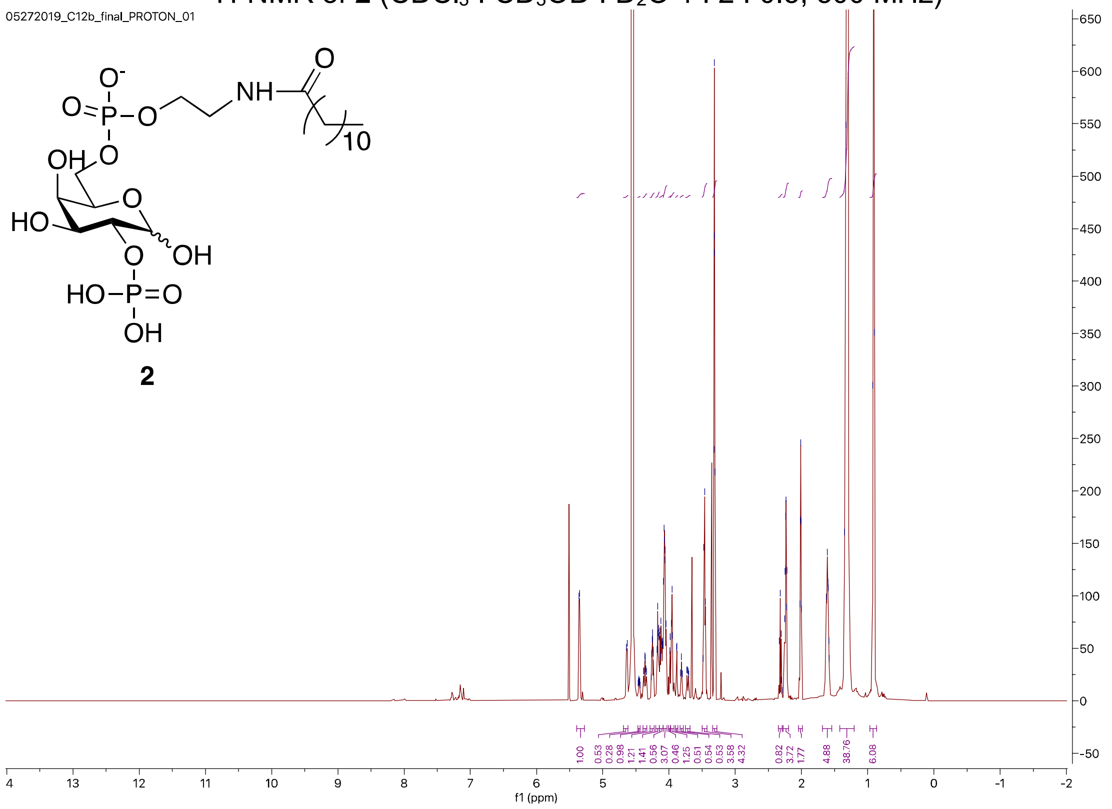

^{13}C-NMR of **2** ($CDCl_3$: CD_3OD : D_2O 1 : 2 : 0.5, 125 MHz)

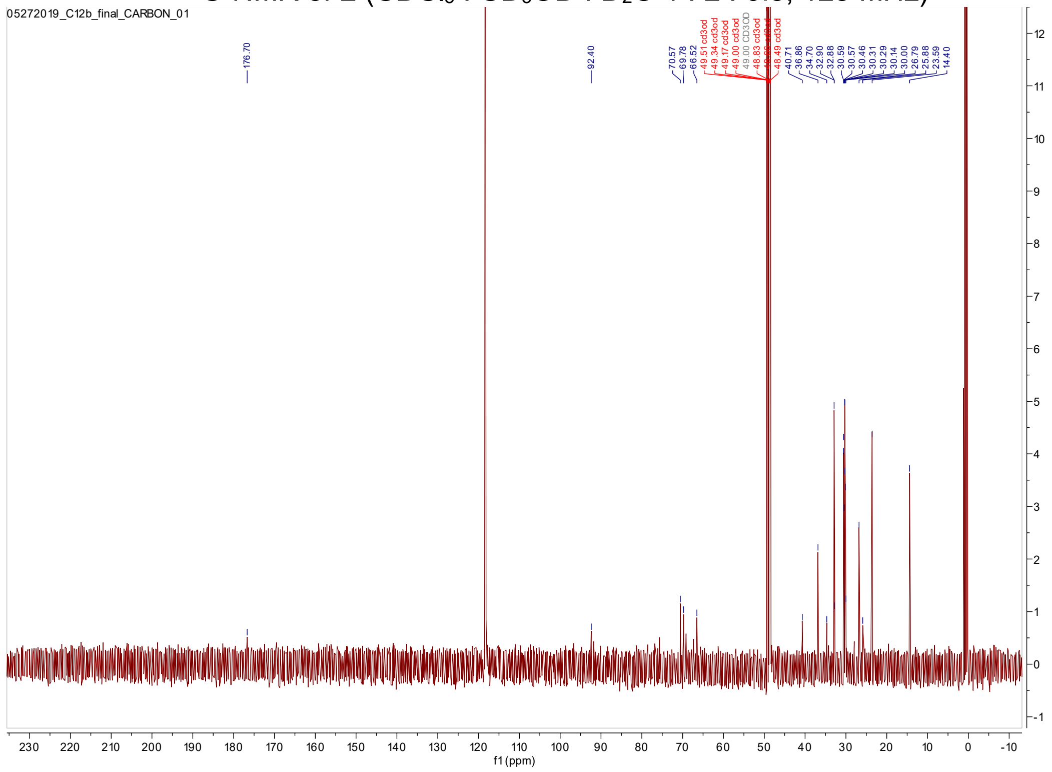

^{1}H-NMR of **3** ($CDCl_3$: CD_3OD : D_2O 1 : 2 : 0.5, 500 MHz)

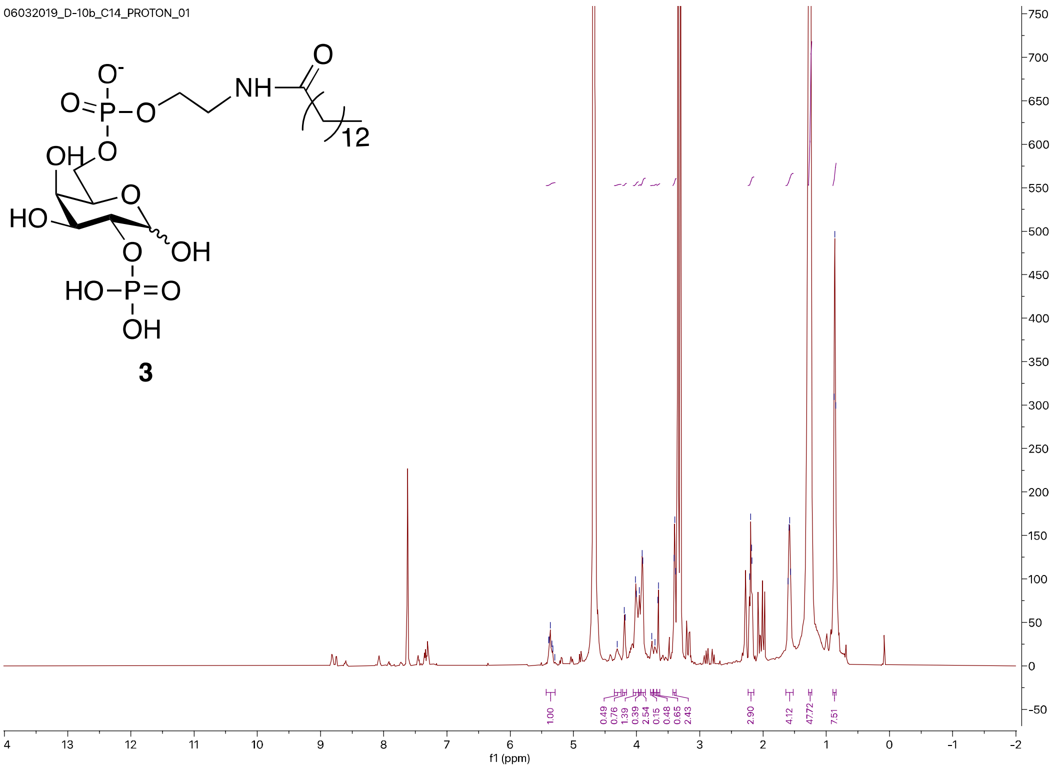

^{13}C-NMR of **3** ($CDCl_3$: CD_3OD : D_2O 1 : 2 : 0.5, 125 MHz)

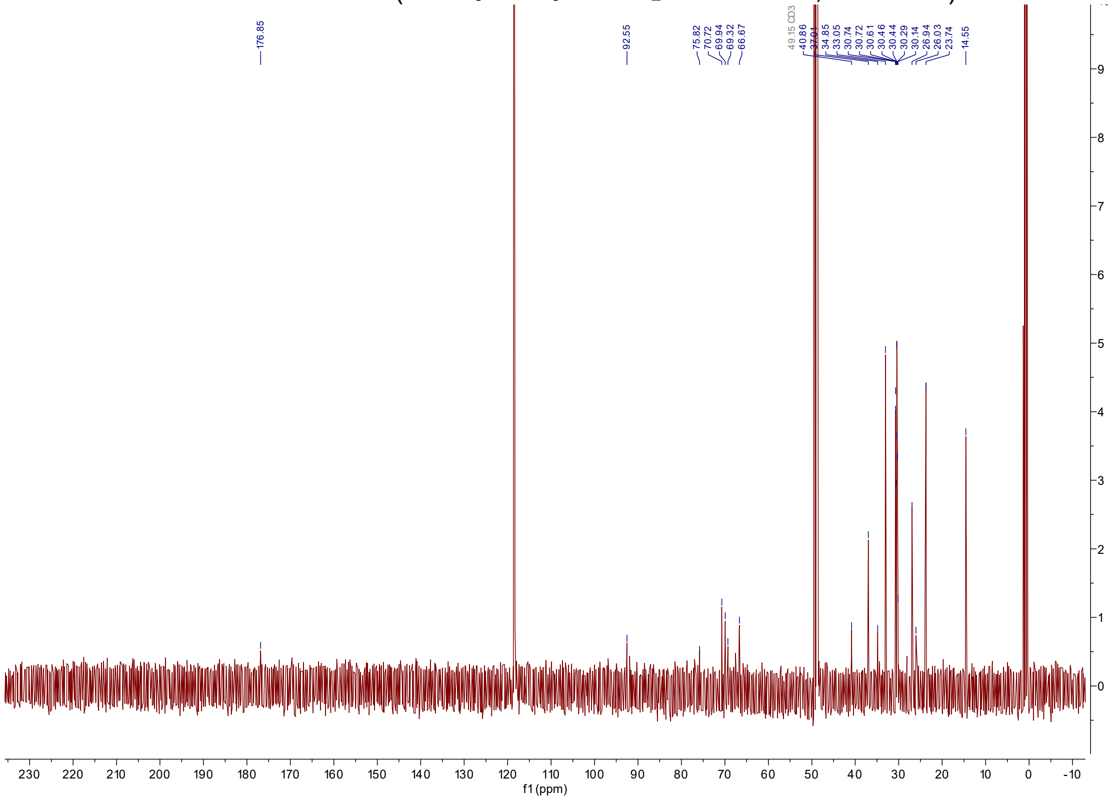

^{1}H-NMR of **4** ($CDCl_3$: CD_3OD : D_2O 1 : 2 : 0.5, 500 MHz)

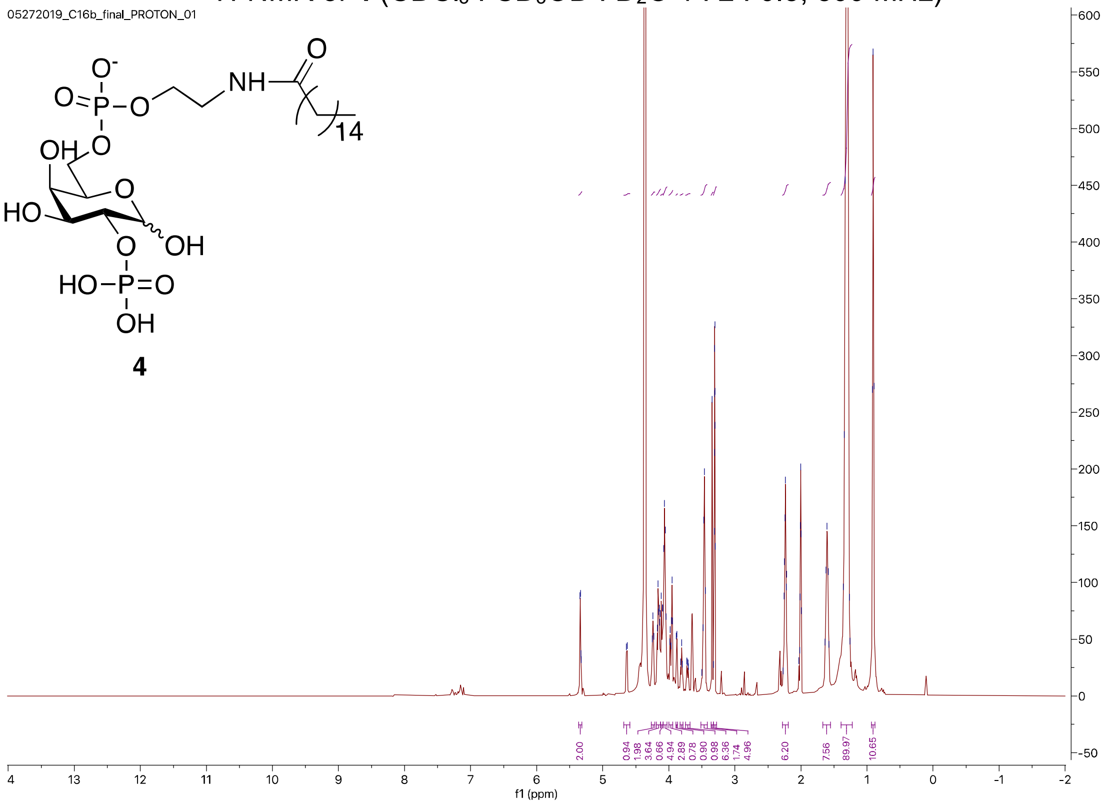

^{13}C-NMR of **4** ($CDCl_3$: CD_3OD : D_2O 1 : 2 : 0.5, 125 MHz)

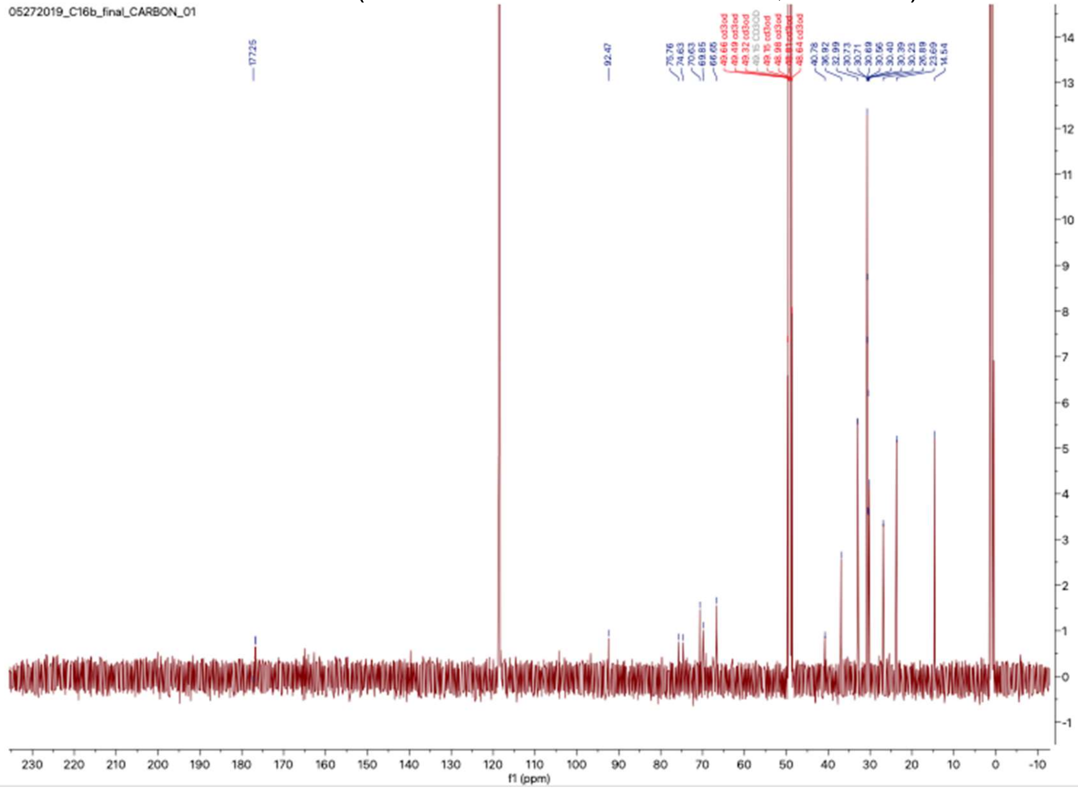

^{1}H-NMR of **25** ($CDCl_3$, 500 MHz)

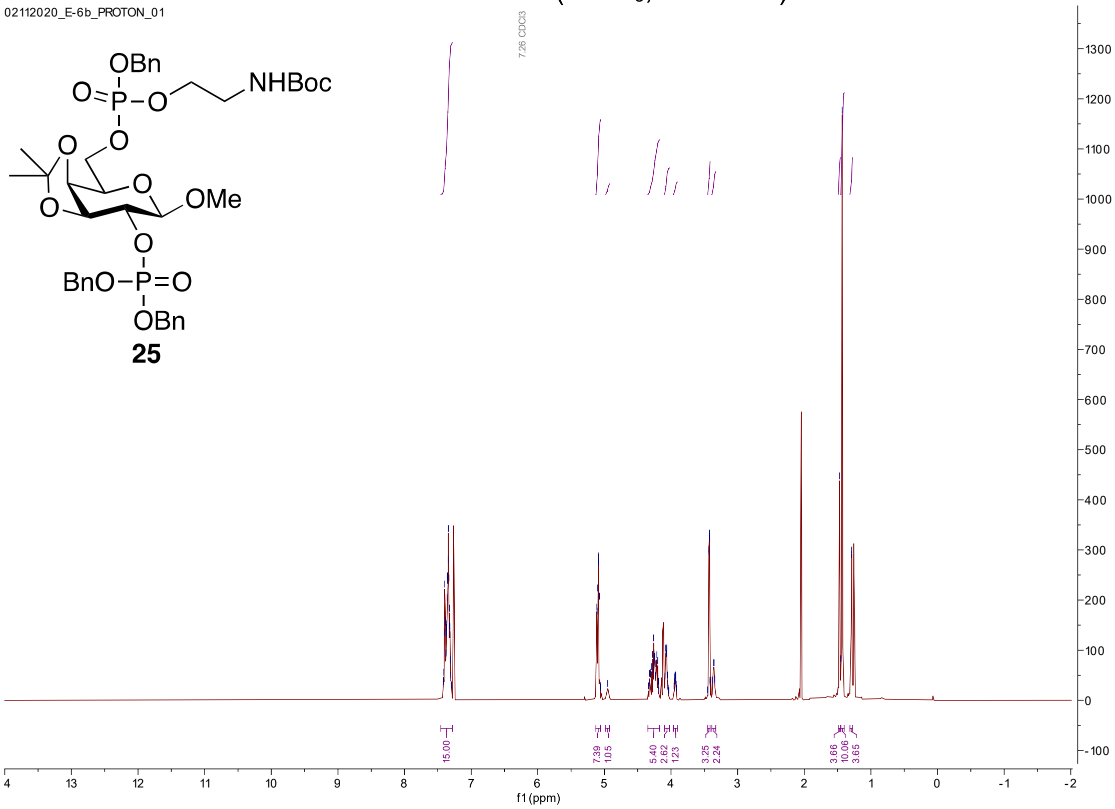

^{13}C-NMR of **25** ($CDCl_3$, 125 MHz)

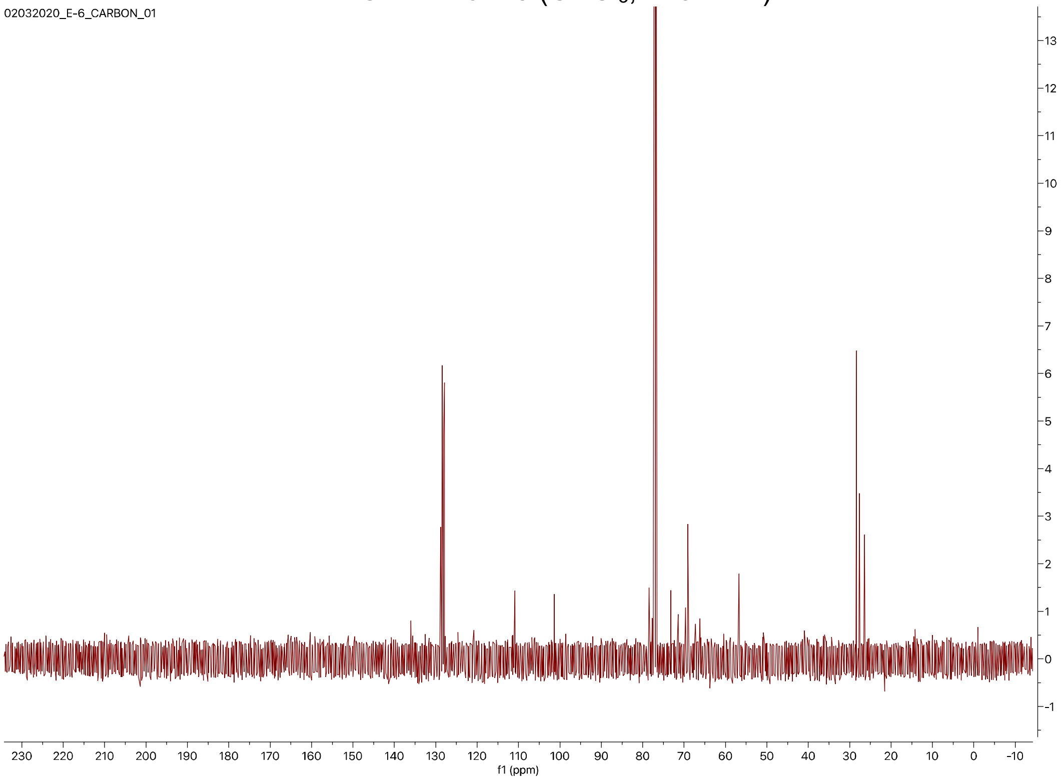

^{1}H-^{1}H gCOSY of **25** ($CDCl_3$, 500 MHz)

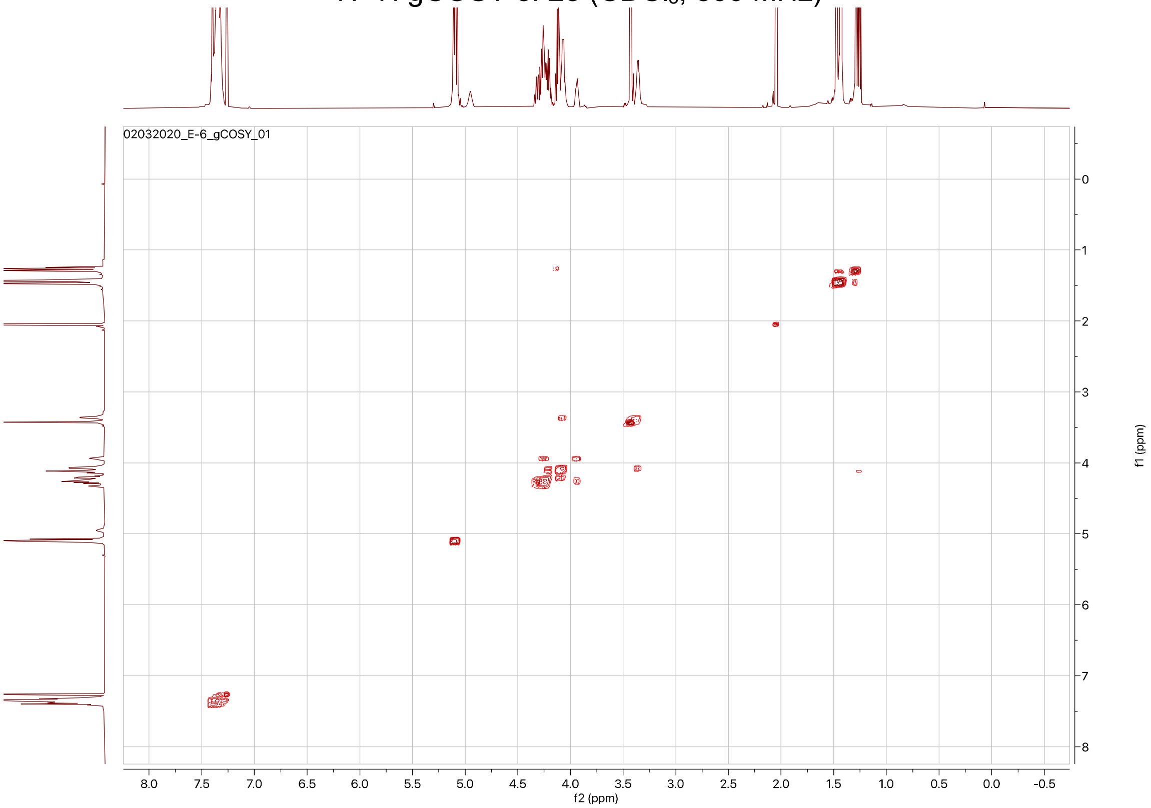

^{1}H-^{13}C gHSQCAD of **25** ($CDCl_3$, 500 MHz)

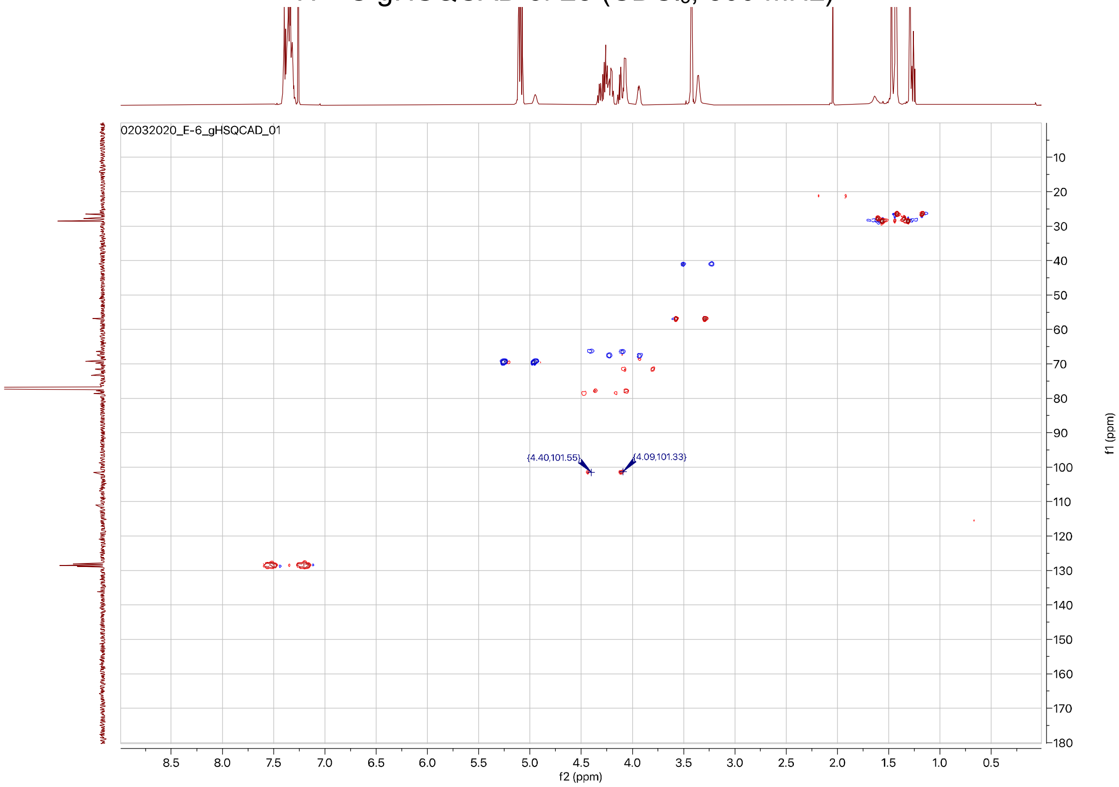

^{1}H-NMR of **5** ($CDCl_3$: CD_3OD : D_2O 1 : 2 : 0.5, 500 MHz)

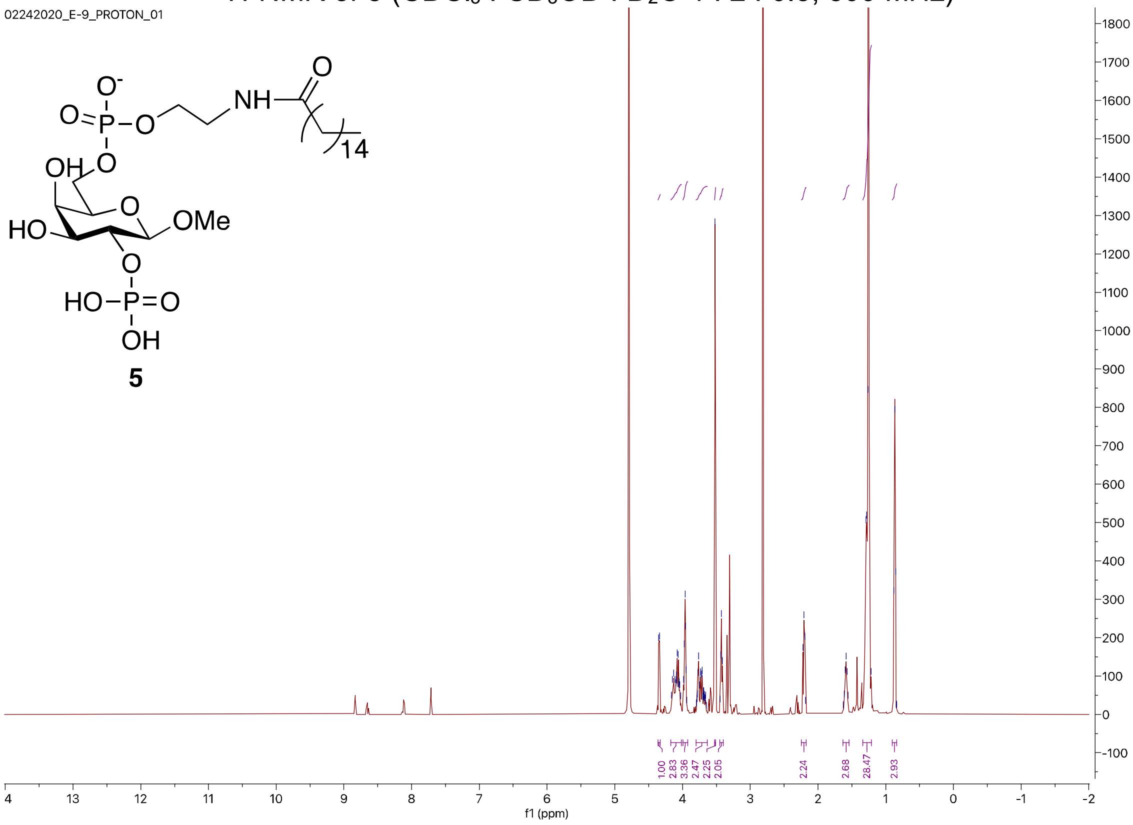

^{13}C-NMR of **5** ($CDCl_3$: CD_3OD : D_2O 1 : 2 : 0.5, 125 MHz)

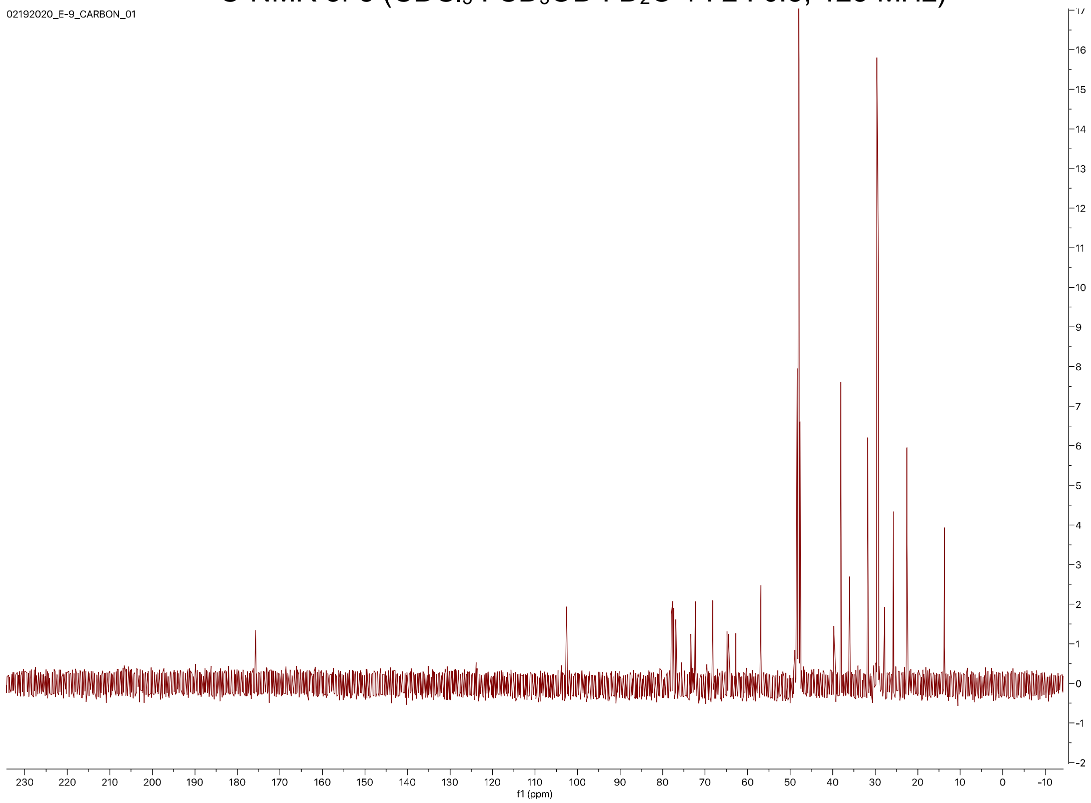

^{1}H-NMR of **6** ($CDCl_3$: CD_3OD : D_2O 1 : 2 : 0.5, 500 MHz)

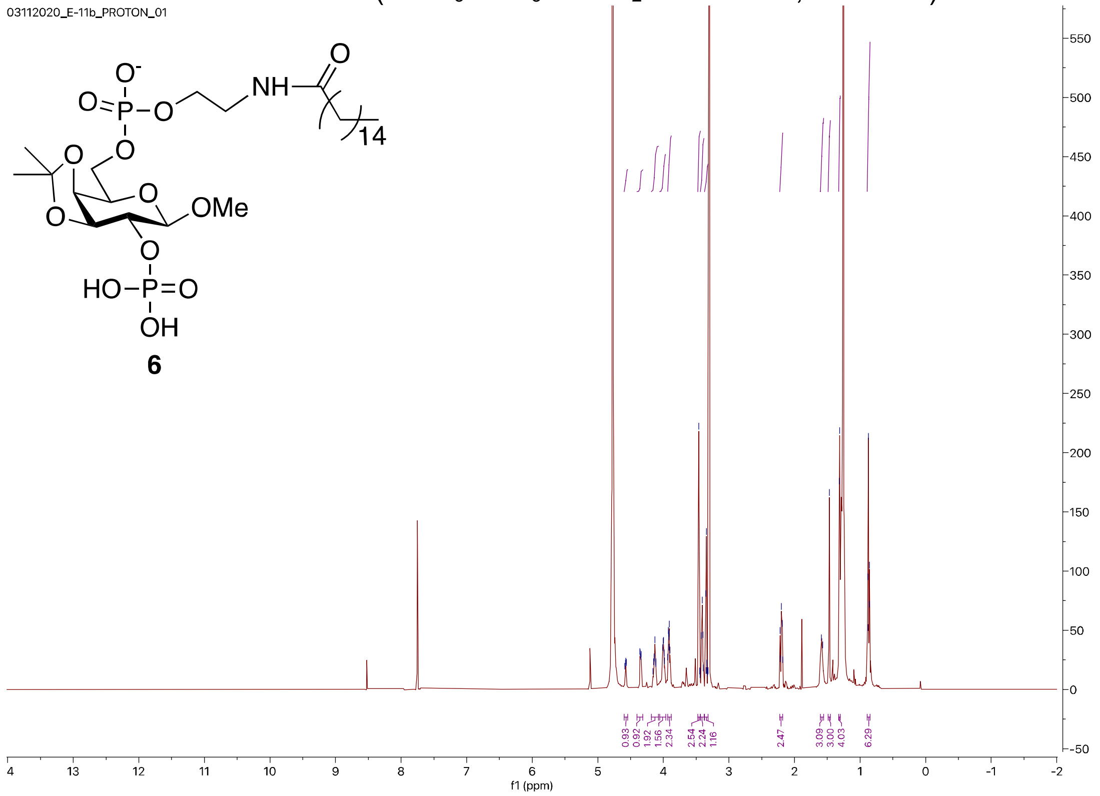

^{13}C-NMR of **6** ($CDCl_3$: CD_3OD : D_2O 1 : 2 : 0.5, 125 MHz)

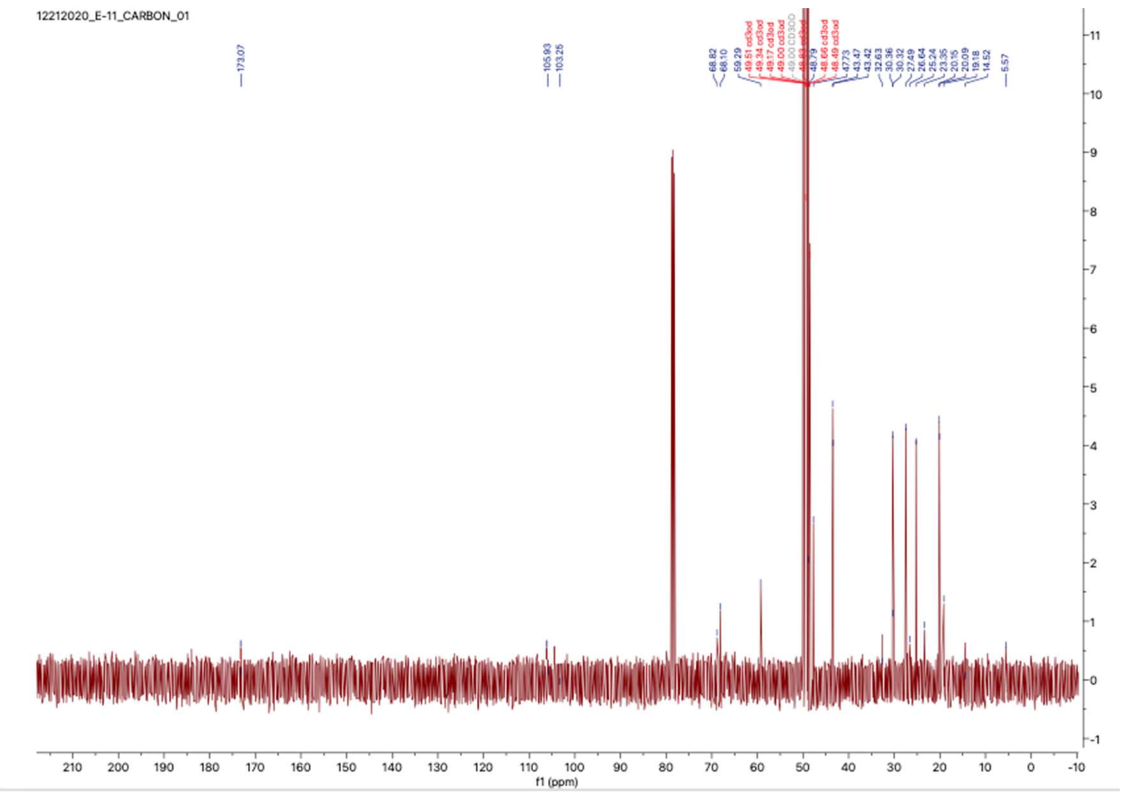
12212020_E-11_CARBON_01
f1 (ppm)
